# Engineered Metal Nanoparticles: A Possible Small Solution to Big Problems Associated with Toxigenic Fungi and Mycotoxins

**DOI:** 10.3390/toxins17080378

**Published:** 2025-07-30

**Authors:** Eva María Mateo, Fernando Mateo, Andrea Tarazona, Misericordia Jiménez

**Affiliations:** 1 Department of Microbiology and Ecology, Faculty of Medicine and Odontology, University of Valencia, 46010 Valencia, Valencia, Spain; 2 Department of Electronic Engineering, ETSE, University of Valencia, 46100 Burjassot, Valencia, Spain; fernando.mateo@uv.es; 3 Department of Microbiology and Ecology, Faculty of Biology, Burjassot, University of Valencia, 46100 Valencia, Valencia, Spain; andrea.tarazona@uv.es

**Keywords:** metal nanoparticles, toxigenic fungi, mycotoxins, prevention, control

## Abstract

Mycotoxins are secondary metabolites produced primarily by certain species of the genera *Aspergillus*, *Fusarium*, *Penicillium*, *Alternaria*, and *Claviceps*. Toxigenic fungi and mycotoxins are prevalent in staple foods, resulting in significant economic losses and detrimental impacts on public health and food safety. These fungi demonstrate remarkable adaptation to water and heat stress conditions associated with climate change, and the use of synthetic antifungals can lead to the selection of resistant strains. In this context, the development of novel strategies for their prevention and control of food is a priority objective. This review synthesizes the extant knowledge concerning the antifungal and anti-mycotoxin potential of the primary metal nanoparticles (silver, copper) and metal oxide nanoparticles (copper oxide and zinc oxide) studied in the literature. It also considers synthesis methods and the lack of consensus on technical definitions and regulations. Despite methodological gaps and the scarcity of publications analyzing the effect of these NPs on fungal growth and mycotoxin production simultaneously, it can be concluded that these NPs present high reactivity, stability, and the ability to combat these food risks. However, aspects related to their biosafety and consumer acceptance remain major challenges that must be addressed for their implementation in the food industry.

## 1. Introduction

The extent of fungal diversity remains to be elucidated. It is estimated that the number of species ranges from 2.2 to 3.8 million [1], and up to 5.1 million [2]. Fungal diseases are responsible for more than 1.5 million deaths per year, which is more than three times the number of deaths caused by malaria and comparable to the number of deaths caused by tuberculosis [3] and breast cancer [4]. Human fungal pathogens are regarded as the “hidden killers” of the medical community, given their ability to cause a multitude of infections and complications in human health [3,4,5,6,7]. The accelerated proliferation of fungal infections is often associated with climate change, the virulence of the pathogens, and the increasing prevalence of immunocompromised patients worldwide [8,9]. Therefore, the timely recognition and treatment of such infections are of significant importance and necessitate the availability of comprehensive information in this regard.

Within the domain of food security and safety, toxigenic fungi emerge as the most significant pathogens of global concern [10]. These fungi, belonging to the genera *Aspergillus*, *Fusarium*, *Penicillium*, *Alternaria*, and *Claviceps* [11], can, under certain conditions, produce mycotoxins that accumulate in grains, fruits, and other foodstuffs. Mycotoxins are highly toxic compounds affecting both humans and animals. The mechanisms of action and health effects of mycotoxins on human and animal health have been the subject of study for many years. Most mycotoxins are known to exert harmful effects on animals and/or humans, such as immunotoxic, genotoxic, cytotoxic, neurotoxic, teratogenic, or carcinogenic outcomes [12,13,14,15,16,17,18]. Toxigenic fungi are generally phytopathogens, and their mycotoxins commonly contaminate staple foods in both human and animal diets [19,20,21]. These fungi and their toxins substantially impact the economy and public health [22,23,24,25], causing significant damage to crops [26]. Losses from contamination of agricultural commodities by fungi and mycotoxins pose serious threats to food safety [12,27,28]. Mycotoxin contamination is particularly alarming in regions heavily reliant on staple foods such as grains, cereals, and nuts. Safeguarding a sustainable food supply depends on preventing mycotoxin contamination.

Within the domain of crop mycobiota, there is a close relationship between the prevailing fungal populations, regional climatology, agricultural practices, and the types and levels of mycotoxins present. Cereals and cereal products such as bread, pasta, breakfast cereals, cake, snacks, beer, infant food, complete feed, and pet foods harbor the greatest diversity of mycotoxin-producing species simultaneously, and consequently, the widest array of mycotoxins [29,30,31,32,33,34,35,36]. However, other commonly consumed foods, beverages, or food additives also represent important sources of mycotoxins. These include nuts and dried fruits [37,38,39,40,41,42,43,44], coffee [45,46,47,48,49,50], cocoa [51], spices [52,53,54,55], milk and derivatives [56,57,58,59], grapes and grape-derived items [60,61,62,63], meat and meat products [64,65,66,67,68], among others.

Several hundred mycotoxins are known; however, only a subset is regulated in the European Union (EU) for specific foods and beverages considered most susceptible to contamination, as they consist of raw materials or their derivatives that commonly host the producing fungi [19,69,70,71]. The mycotoxins regulated in the EU and their principal fungal sources are as follows: aflatoxin B_1_ (AFB_1_), the sum of aflatoxins B_1_, B_2_, G_1_, and G_2_ (AFB_1_, AFB_2_, AFG_1_, and AFG_2_), and aflatoxin M_1_ (AFM_1_) produced by *Aspergillus flavus*, *A. parasiticus*, and *A. nonius*; ochratoxin A (OTA) produced by *A. niger*, *A. carbonarius*, *A. welwitschiae*, *A. steynii*, *A. ochraceus*, *A. westerdijkiae*, *Penicillium verrucosum*, and *P. nordicum*; the sum of fumonisins B_1_ and B_2_ (FB_1_ and FB_2_) produced by *Fusarium verticillioides*, *F. proliferatum* and *A. niger*; patulin (PAT) produced by *P. expansum*; deoxynivalenol (DON) and zearalenone (ZEA) formed by *F. graminearum* and *F. culmorum*; citrinin (CIT) produced by *Penicillium citrinum*, *Monascus purpureus*, and *M. ruber*; the sum of T-2 and HT-2 toxins (T-2 and HT-2) produced by *F. sporotrichioides* and *F. langsethiae*; and ergot alkaloids (EA) associated with *Claviceps* spp. These regulated mycotoxins represent those most frequently detected in susceptible foodstuffs and are under EU control due to their established toxicity profiles and prevalence. In addition, the content of ergot sclerotia in unprocessed cereal grains is also regulated in the EU [72,73,74]. Other unregulated mycotoxins are attracting growing attention from the scientific community. These include the *Fusarium* mycotoxins nivalenol (NIV), diacetoxyscirpenol (DAS), enniatins (ENs), beauvericin (BEA), moniliformin, and fusaproliferin; the *Aspergillus* toxins sterigmatocystin and emodin; and the *Alternaria* metabolites alternariol and tenuazonic acid [75,76].

Toxigenic fungi are highly competitive species that adapt well to water and heat stress conditions associated with climate change [76,77,78]. For example, aflatoxin-producing strains of *Aspergillus flavus* show optimal growth temperatures and aflatoxin production at temperatures between 33 °C and 37 °C, under low water activity (a_w_) conditions [79,80,81]. Moreover, the simultaneous presence of multiple mycotoxins within an agricultural commodity leads to interactions (antagonistic, additive, or synergistic) that can amplify toxicity beyond what is observed from individual toxins alone [82,83,84,85,86].

**Figure 1:**
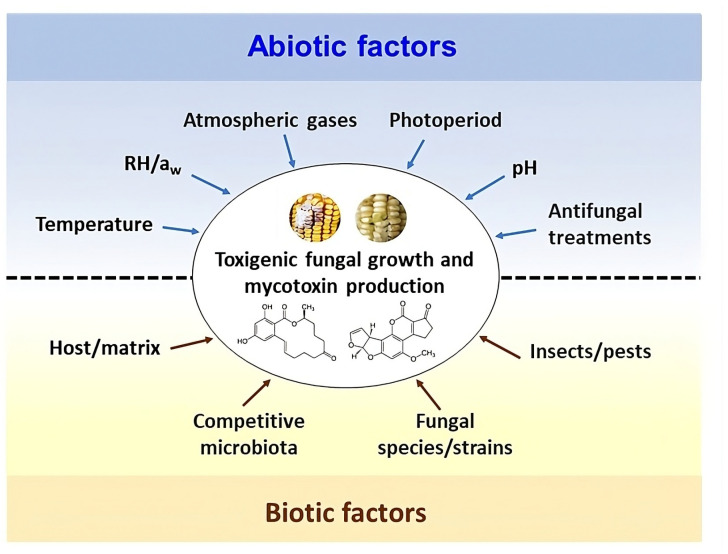
Main factors affecting the growth of toxigenic fungi and mycotoxin production in foods.

Preventing and controlling fungal growth and mycotoxin production and accumulation in food is not an easy task because many biotic and abiotic factors and mutual interactions are involved [87,88] (Figure 1).

The elimination of mycotoxins from foodstuffs is a multifaceted process, given their remarkable resistance to various food-processing techniques, including cooking, boiling, baking, frying, roasting, pasteurization, and extrusion [89]. Consequently, in recent years, machine learning (ML) methods have been investigated to predict fungal growth and mycotoxin production by relevant toxigenic fungi under different environmental conditions [90,91,92,93,94,95,96,97,98].

Despite extensive scientific efforts to elucidate the complex interactions between fungi and the biotic-abiotic factors of their environment (Figure 1), the risks posed by toxigenic fungi and mycotoxins in foods have not been eliminated; in some cases, they have increased and are expected to continue rising in the medium term under new climate change scenarios [76,77,78]. The latest Intergovernmental Panel on Climate Change (IPCC) Sixth Assessment report confirms that global warming is unequivocal, accompanied by unprecedented climatic changes [99]. These environmental shifts can significantly influence the life cycles of toxigenic fungi, altering host resistance and host–pathogen interactions [100,101]. As a result, the resilience of diverse toxigenic species and their capacity for mycotoxin synthesis could be profoundly affected.

Undoubtedly, the primary strategy for mitigating the risks associated with toxigenic fungal colonization of food and mycotoxin accumulation is to prevent fungal growth at all stages of the food chain, including pre-harvest and post-harvest processes such as drying, transportation, packaging, storage, and marketing. A diverse array of physical, chemical, and biological methods has been employed to control mycotoxin contamination, including innovative and emerging technologies such as ionizing and non-ionizing radiation, cold plasma, pulsed light, ultrasound, pulsed electric fields, high-pressure processing, and essential oils [102,103]. Among these methods, the use of antifungal compounds remains the most effective strategy.

Conventional synthetic antifungals have several drawbacks: they produce toxic residues that can harm the environment, beneficial microbes, and both humans and animals [104]. Additionally, their use can lead to the development of resistant strains. Furthermore, low doses of certain fungicides may sometimes promote mycotoxin production by specific fungal species [105,106].

Despite the existence of a variety of commercial synthetic antifungal agents, their utilization is constrained by factors such as their toxicity and the emergence of multidrug-resistant strains [27,104,107,108,109,110]. The rate of antifungal resistance development has been characterized as “unprecedented.” This phenomenon can be attributed, at least in part, to the considerable medical advancements that have transpired over the past few decades. Examples of such advancements include the discovery of antibiotics, significant progress in cancer treatment, and the development of surgical transplants. The HIV epidemic and the global pandemic of the novel coronavirus (SARS-CoV-2) have led to a notable increase in the number of individuals with compromised immune systems. This rise has consequently resulted in a shift in the epidemiology of fungal infections, which have transitioned from being sporadic etiological agents of disease to becoming a significant contributor to human morbidity and mortality on a global scale. Individuals with compromised immune systems are predisposed to a greater incidence of fungal infections in comparison with those who are healthy [108,111]. Currently, there are approximately 80 types of antifungal drugs available for clinical use. These compounds can be categorized into five classes, including polyenes, allylamines, azoles, pyrimidine analogues, and echinocandins. In comparison with antibiotics, the number of antifungal agents is limited. Azoles are utilized for a variety of purposes, including the protection of human, animal, and crop health, as well as the formulation of antifouling coatings and wood preservatives [112]. The extensive utilization of azoles has accelerated the emergence of azole-resistant fungi, which has substantial ramifications for human health and food security [27,112]. Fungi have developed several resistance mechanisms. They include alterations of drug targets and cellular pathways (e.g., sterol biosynthesis), reductions of intercellular concentrations of target enzymes, overexpression of the antifungal drug target, activation of stress response signaling, and overexpression of efflux pump proteins. Furthermore, fungi possess intrinsic mechanisms of resistance to antifungal drugs. These mechanisms include biofilm formation, variations in cellular permeability, and many processes that overlap with those involved in acquired resistance. These include target incompatibility, stress response signaling, and the expression of efflux pump proteins [27,113,114]. A pre-harvest strategy to mitigate mycotoxin presence in plant-derived foods involves resistance to fungal infection by toxigenic fungi. Genetic modifications have led to the development of plant varieties that exhibit full or partial resistance to Fusarium spp. infection. For example, overexpression of the antifungal gene HvNEP-1 in the endosperm renders barley less susceptible to Fusarium head blight, resulting in lower mycotoxin levels in the grain [115]. Highly resistant wheat cultivars can convert DON into deoxynivalenol-3-glucoside, a compound that is less toxic than DON [116]. A significant reduction in total mycotoxin content has been observed in transgenic maize cultivars compared to their non-transgenic counterparts. These findings suggest that the consumption of transgenic maize may pose a reduced risk of mycotoxin contamination [117].

The lack of efficacious antifungal drugs, combined with mounting resistance, has created an urgent need for novel treatments. Consequently, alternative eco-friendly and effective agents are under continuous investigation. Developing antifungal strategies that are economically viable, sustainable, and safe presents a significant challenge. In this context, nanotechnology holds considerable potential to advance these strategies. Combining the use of resistant cultivars with approaches that reduce mycotoxin accumulation and biosynthesis can lower mycotoxin levels in cereals [118].

The field of nanotechnology is defined as the scientific and engineering discipline focused on the design, fabrication, and utilization of structures, devices, and systems through the manipulation of atoms and molecules at the nanoscale level. The synthesis, management, and application of nanomaterials fall under the interdisciplinary umbrella of nanotechnology. In the 21st century, this field has experienced rapid development, leading to significant advancements across various scientific disciplines. Nanomaterials have garnered considerable attention due to their unique properties and extensive applications in multiple sectors. They are utilized in food processing and food preservation/packaging, food fortification, functional food additives, and sensors [119,120,121,122]; in agriculture, including insecticides, herbicides, fungicides, fertilizers, and plant growth regulators, as well as in food safety, water purification, and biosensors for agronomic troubleshooting [123,124,125,126,127,128,129,130,131,132,133,134,135,136,137,138,139,140,141,142,143]; in medicine, encompassing drug delivery, diagnostics, tissue engineering, antimicrobials, and gene delivery [144,145,146,147,148,149]; in environmental applications, including bioremediation and catalysts in environment [129,150,151,152,153,154,155], and various other industries, such as electronics, automotive, defense, and cosmetics [156].

Currently, nanoparticles (NPs) with antifungal properties are highly valued for their diverse applications. However, their use in the agri-food sector remains largely unexplored [157]. Specifically, there is a need to review the existing knowledge regarding their effects on mycotoxin-producing species and the production of mycotoxins. The fields of nanotechnology and toxigenic fungi intersect in various ways, particularly in the context of addressing mycotoxins. Research indicates that NPs represent a promising strategy for controlling toxigenic fungi and mycotoxins in food [158]. This approach not only enhances the efficacy of antifungal compounds but also reduces toxicity, improves stability, and facilitates targeted delivery.

In light of the contemporary challenges posed by toxigenic fungi and mycotoxins in food products, addressing this complexity necessitates the development of innovative management strategies. This review aims to provide a comprehensive overview of the potential of metallic nanoparticles (MNPs) as effective agents for the prevention and control of toxigenic fungi and mycotoxins in food products. Specifically, this review focuses on silver nanoparticles (AgNPs), copper nanoparticles (CuNPs), copper oxide nanoparticles (CuONPs), and zinc oxide nanoparticles (ZnONPs), as these have demonstrated the highest efficacy in existing studies. The chemical and thermal stability of inorganic NPs is superior to that of organic NPs, making them more suitable for storage, transportation, and use in harsh environments.

## 2. Engineered Nanomaterials Against Toxigenic Fungi and Mycotoxin Production

### 2.1. Definition of Nanoparticle

What exactly constitutes a nanoparticle (NP) remains a topic of considerable debate. The International Organization for Standardization (ISO) classifies nanoparticles as nano-objects, which are materials whose external dimensions fall within the nanoscale. If these dimensions exceed the nanoscale by a factor of three or more, they are more accurately referred to as “nanofibers” or “nanoplates” rather than nanoparticles [159]. Similarly, the Scientific Committee on Consumer Products (SCCP) defines NPs as particles with at least one dimension ranging from 1 to 100 nm [160]. Nevertheless, there is still no universal agreement on the definition of “nanoparticle,” which complicates related processes [161].

Nanomaterials can originate from natural sources, arise unintentionally through human activities, or be deliberately engineered to exhibit novel properties, such as enhanced strength, chemical reactivity, or conductivity, due to their nanoscale structure. The European Union has enacted legislation to clearly define these materials and assess the potential health and environmental risks associated with their applications. Additionally, the risk assessment bodies and agencies of the European Union have developed guidance for evaluating nanomaterials. The Commission Recommendation C/2022/3689 [162] provides a comprehensive definition of nanomaterials that revises and replaces the previous nanomaterial definition established in Commission Recommendation 2011/696/EU [163]. According to this recommendation, ’Nanomaterial’ refers to a “natural, incidental or manufactured material consisting of solid particles that are present, either on their own or as identifiable constituent particles in aggregates or agglomerates, and where 50% or more of these particles in the number-based size distribution fulfill at least one of the following conditions: (a) one or more external dimensions of the particle are in the size range of 1 nm to 100 nm; (b) the particle has an elongated shape, such as a rod, fiber or tube, where two external dimensions are smaller than 1 nm and the other dimension is larger than 100 nm; (c) the particle has a plate-like shape, where one external dimension is smaller than 1 nm and the other dimensions are larger than 100 nm”.

Specifically regarding food, Commission Regulation (EU) 2015/2283 [164] defines “engineered nanomaterial” as any intentionally produced material with one or more dimensions on the order of 100 nm or less, or composed of discrete functional parts, internally or at the surface, many of which have one or more dimensions on the order of 100 nm or less, including structures, agglomerates, or aggregates that may exceed 100 nm in size but retain properties characteristic of the nanoscale. This aligns closely with Commission Recommendation C(2022)3689 [159], but key distinctions include the following: (1) its specific reference to “engineered” nanomaterials, and (2) the absence of a minimum percentage threshold (e.g., ≥50%) for classification. To support implementation, some measures have been recommended: (1) reviewing modern methodologies; (2) advancing analytical method development and validation for the detection of nanomaterials in food; and (3) providing Member States with training and analytical support. Furthermore, different manufacturing methods for engineered nanoparticles can yield variations in loading capacity, delivery efficiency, and shelf life.

In the academic literature, nanomaterials are commonly categorized by morphology, size, and chemical composition [165]. Based on chemical composition, NPs are typically grouped into three categories: inorganic, organic, and carbon-based.

Inorganic NPs are defined by the absence of carbon-based compounds. Examples include metal, ceramic, and semiconductor NPs. MNPs, whether single-metal, bimetallic, or polymetallic, are composed of metal atoms, whereas metal oxide nanoparticles (MONPs) consist of metal atoms bonded to oxygen [166,167]. These materials exhibit unique optical, electrical, thermal, magnetic, and biological properties, making them highly valuable across disciplines such as physics, chemistry, biology, biomedical engineering, and pharmaceutical sciences [165,168].

Organic NPs are derived from biological or synthetic substances such as proteins, carbohydrates, lipids, and polymers (e.g., chitosan, cellulose, and proteins). Common types include liposomes, micelles, dendrimers, and protein-based complexes like ferritin. Generally biodegradable and non-toxic, many of these NPs feature hollow cores, such as in liposomes, allowing for encapsulation of active compounds. While they are sensitive to environmental factors like heat and light, their surface functionality, physicochemical stability, and loading capacity make them ideal candidates for biomedical applications, including targeted drug delivery and cancer therapy [165,169].

Carbon-based NPs are composed entirely of carbon and encompass structures such as fullerenes, graphene, carbon nanotubes, carbon nanofibers, carbon black, and occasionally nanoscale activated carbon [170]. These materials are valued for their electrical conductivity, mechanical strength, electron affinity, and sorption capacity. They find application in diverse fields such as drug delivery, energy storage, bioimaging, photovoltaics, environmental sensing, and microbial detection. Emerging carbon nanostructures, including nanodiamonds and carbon nano-onions, demonstrate low toxicity and high biocompatibility, which supports their expanding role in drug delivery and tissue engineering [165]. Globally, over 232 nanotechnology-enabled products, developed by 75 companies in 26 countries, have been identified in the food and agriculture sectors [171].

### 2.2. Engineered Nanoparticles as Antifungal Systems

Toxigenic fungi and mycotoxin contamination of food impact multiple sectors: agriculture (crop losses due to fungal infections and mycotoxin accumulation), the economy, the food industry (contamination of stored grains, nuts, dairy products, etc.), and medicine (mycotoxicosis and opportunistic fungal infections often resistant to standard antifungal treatments). While nanomaterials offer promise as antifungal delivery systems to control toxigenic fungi and mycotoxins in food [169], their application must remain simple, effective, sustainable, and devoid of health hazards. Prior research has primarily centered on engineered MNPs that exhibit intrinsic antifungal properties [134,135,172].

Other nanomaterials hold promise as antifungal systems in food, albeit with less extensive study than MNPs. Examples include the following: polymeric NPs (e.g., biodegradable polymers like chitosan for controlled release that protect antifungal compounds) [173]; nanoemulsions [174]; solid lipid nanoparticles (lipid-based carriers that improve drug stability and bioavailability) [175]; nanogels (hydrophilic networks that can encapsulate both hydrophilic and hydrophobic antifungal agents) [176]; liposomes (phospholipid vesicles that encapsulate antifungals for targeted delivery); biocompatible lipid vesicles that increase drug solubility and reduce toxicity [177]; cyclodextrin inclusion complexes (that heighten solubility and slow release) [178]; and dendrimers (branched nanostructures offering high drug loading capability and targeted delivery) [179].

The use of MNPs enhances antifungal activity and reduces mycotoxin contamination through several mechanisms: (1) increasing the solubility and bioavailability of antifungal agents; (2) allowing for the sustained release of compounds; (3) protecting molecules from degradation; (4) effectively targeting fungi; and (5) reducing dosages and side effects, thereby minimizing toxicity and environmental impact [88,158,169,172,180,181,182,183]. MNPs have garnered significant attention as novel antimicrobial agents. Researchers are currently investigating their effectiveness against toxigenic fungi and mycotoxins. This research aims to evaluate their potential as a strategy for addressing these challenges [120,172,184,185,186].

Engineered metal- and metal oxide-based NPs (MNPs and MONPs, respectively) are primarily inorganic NPs that can help combat antibiotic resistance [187]. MNPs are synthesized using both “top-down” and “bottom-up” approaches. The most commonly used metals include silver (Ag), copper (Cu), zinc (Zn), gold (Au), aluminum (Al), lead (Pb), cadmium (Cd), cobalt (Co), and iron (Fe). MONPs modify the properties of MNPs to enhance their reactivity and efficacy. Common metal oxides include copper oxide (CuO), zinc oxide (ZnO), iron oxide (Fe_2_O_3_), aluminum oxide (Al_2_O_3_), cerium oxide (CeO_2_), magnetite (Fe_3_O_4_), titanium dioxide (TiO_2_), and silicon dioxide (SiO_2_).

NPs may exhibit superior properties compared to their metal counterparts [126,170]. In addition to the formulation process, the resulting size and shape of NPs are crucial to their activity. NPs possess unique characteristics, including increased surface area, pore size, and charge density on their surfaces, all of which contribute to their distinctive properties. Moreover, NPs can exhibit a variety of shapes, colors, crystalline and amorphous structures, and sensitivity to environmental conditions [168,188]. Size is often one of the most critical factors to consider. Studies have demonstrated that smaller NPs tend to exhibit stronger antifungal properties compared to larger NPs, which can be attributed to their higher surface-area-to-volume ratio. This enhanced ratio improves binding at various target sites, facilitates diffusion, and reduces the aggregation propensity [126,185,189,190]. Currently, the prevention and control of fungal contamination using nanotechnology is a focal point of numerous studies. Despite significant advancements in this area of research, the development of antifungal nanoadditives for food-related systems remains in its early stages. In the agri-food sector, MNPs and MONPs have emerged as the most prevalent types of nanoparticles. The mechanisms of action of these agents differ significantly from those of traditional antibiotics, as they target multiple biomolecules, thereby hindering the development of resistant strains. Additionally, they exhibit activity against fungi that have already developed resistance [191]. In the field of agriculture, the extensive use of nanomaterials, particularly Ag, Cu, and Zn-based nanoformulations, has been adopted to enhance crop productivity and health. These nanomaterials are utilized as nanofertilizers and protect against toxigenic fungi and other harmful organisms [140,141,192,193,194]. However, the absence of science-based regulatory frameworks hinders the effective regulation of their use [142,195].

## 3. Synthesis of MNPs

**Figure 2:**
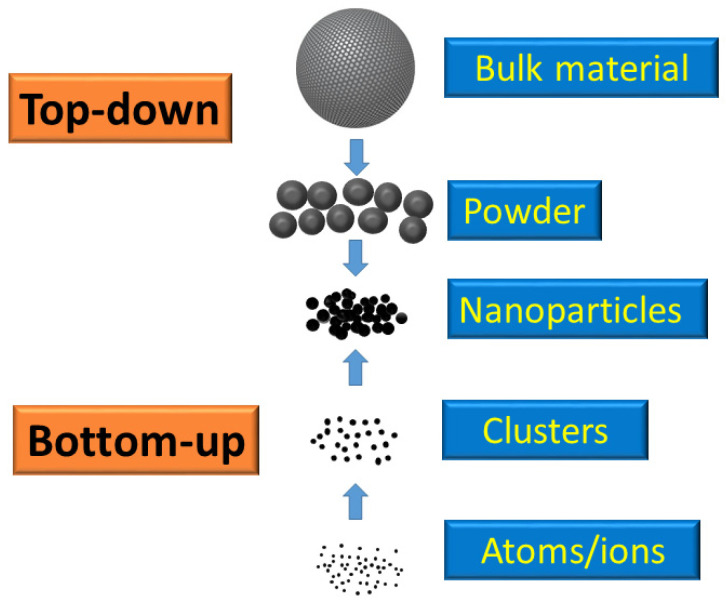
Schema of the two general approaches for NP synthesis (top-down and bottom-up).

As said before, there are two approaches, named ‘top-down’ and ‘bottom-up’ methods, that are primarily utilized for the synthesis of MNPs (Figure 2).

### 3.1. Top-Down Methods

Top-down methods commence with bulk quantities of materials that are subsequently reduced in size and combined with clusters of atoms or ions. Some top-down approaches utilize physical technologies that employ thermal energy, high-energy radiation, and mechanical pressure to facilitate processes such as material condensation, evaporation, abrasion, or melting. These techniques present several advantages over chemical methods, particularly in terms of minimizing solvent contamination in thin films and enhancing the uniformity of NP distribution. Below are some of the more commonly used physical methods.

#### 3.1.1. Mechanical Ball Milling

**Figure 3:**
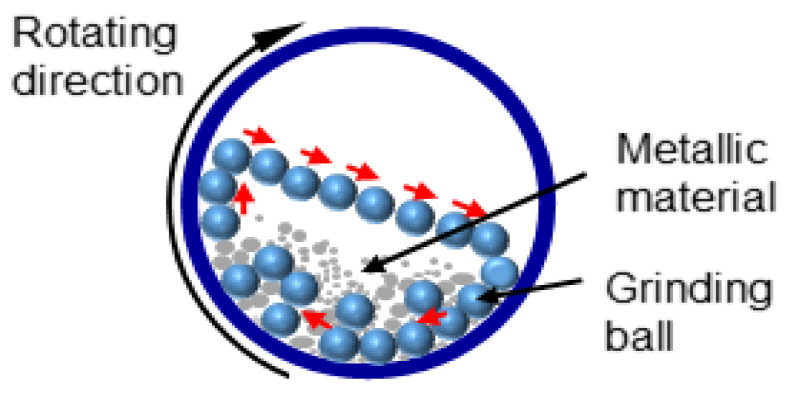
Schematic representation of a ball mill. The red arrows indicate the direction of the grinding balls during operation.

High-energy ball milling is a mechanical technique that reduces bulk metal powders into NPs through intense collisions within a ball mill. This cost-effective and scalable method is widely employed for producing nanocomposites and metal alloys. However, challenges such as controlling particle size and preventing contamination persist. The process involves transferring kinetic energy from the grinding media, typically steel or tungsten carbide balls, to the material. These collisions, along with friction between the balls, the material, and the walls of the mill, generate significant energy, elevating both temperature and pressure within the mill. This energy facilitates the formation of fine powders, with the balls continuously interacting with the evolving particles. Exothermic reactions may occur during milling, producing additional heat and promoting NP formation. Milling devices include planetary, attrition, horizontal, vibrating, low-energy tumbling, and high-energy ball mills [188,196]. Figure 3 illustrates a schematic of a ball mill.

The energy transferred to the powder is influenced by several factors, including the type of mill used, the characteristics of the powder, milling speed, the size and size distribution of the balls, whether the milling is conducted in dry or wet conditions, milling temperature, and milling duration [197]. This method is particularly favored for the synthesis of intermetallic NPs [188]. It has been successfully applied to produce uniform ZnONPs with sizes ranging from 10 to 30 nm. The process operates at ambient temperature and is a straightforward, cost-effective, and solvent-free technique for generating ZnONPs under dry conditions [198]. Moreover, ball milling has proven to be an effective method for synthesizing biochar Ag/MnO nanocomposites [199].

#### 3.1.2. Laser Ablation

**Figure 4:**
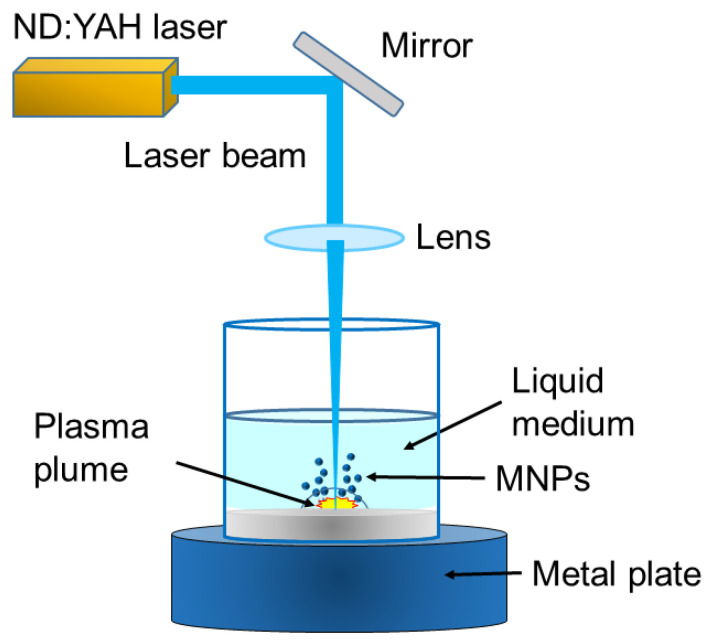
Schema of a laser ablation device.

This technique synthesizes MNPs by vaporizing a solid metal using high-energy laser pulses. It is most effective when conducted in a liquid medium, a process known as Laser Ablation Synthesis in Solution (LASiS). This method is considered “green” because it does not utilize toxic chemical precursors. In LASiS, a pulsed laser beam is focused on a metal target immersed in a liquid. The temperature at the irradiated spot rapidly increases, vaporizing the material and producing a laser-induced plasma plume composed of atoms, ions, electrons, and clusters that expand into the surrounding liquid. The plasma cools and condenses quickly, leading to the formation of clusters and NPs (Figure 4).

Laser ablation can be used to produce various types of NPs. The rapid quenching of vapor facilitates the generation of high-purity NPs within the quantum size range (<10 nm) [200]. The neodymium-doped yttrium aluminum garnet (Nd:YAG) laser operates at different wavelengths (1064, 532, 355, and 266 nm), which can influence the size and distribution of the NPs. Specifically, the 1064 nm wavelength results in larger NPs, while the shorter ultraviolet (UV) wavelengths yield smaller NPs with a narrower size distribution [201].

LASiS can be performed in different liquid media (water, acids, alkalis, organic solvents), targeting materials such as metals, metal alloys, and metal oxides. Water is the most commonly used solvent. The size and morphology of the NPs are significantly influenced by the temperature of the liquid medium. This method produces highly pure materials quickly, is free from contaminants, and is environmentally friendly, as it typically employs mild surfactants in the solvent [202]. However, LASiS can be expensive and energy-intensive, has a low production rate, and may encounter challenges in achieving precise control over NP size. The sizes of NPs can vary based on the type of NP and the experimental conditions, including both the laser source and the liquid medium.

The diversity of NPs generated through laser ablation is greater than that produced by other methods [196]. Laser ablation is a rapid and efficient technique for the production of AgNPs and can be synergistically combined with ball milling methods to enhance AgNP synthesis, while also creating opportunities for the development of novel nanocomposites and functional materials [203].

#### 3.1.3. Sputtering

**Figure 5:**
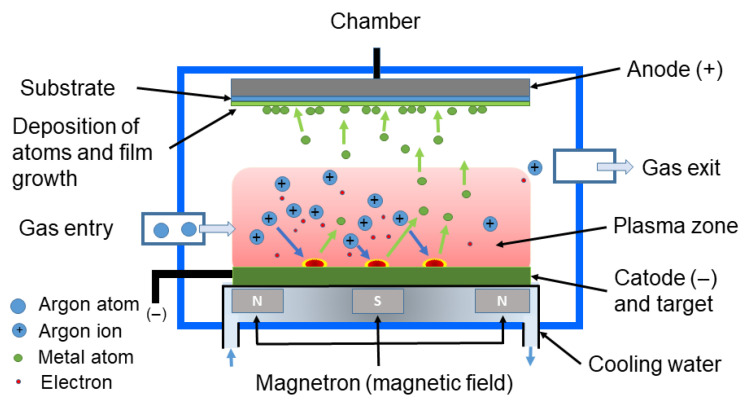
Schema of a magnetron sputtering device. Colored arrows indicate the direction of movement in the chamber (contoured ligh blue for gas flow, dark blue for argon ions, green for metal atoms).

Sputtering is a type of physical vapor deposition (PVD) method used for the growth of thin films. In this process, a high electric field is generated within a chamber, causing high-energy ions to bombard a metal target, which serves as the cathode in a plasma environment. This bombardment results in the ejection of atoms from the cathode, which subsequently deposit onto a substrate (the anode), forming NPs, primarily MNPs and MONPs [204,205]. A noble gas, typically argon (Ar), is introduced into the chamber at low pressure. When the electric field is applied, electrons are accelerated and collide with the Ar atoms, generating argon ions (Ar^+^) and releasing additional electrons. This process produces a plasma, and the Ar^+^ ions within the plasma are attracted to the cathode (the target) due to the electric field (Figure 5).

There are various types of sputtering techniques, including direct current (DC) sputtering, radio frequency (RF) sputtering, magnetron sputtering, and reactive sputtering. The first method employs a constant DC voltage to generate plasma, making it suitable for the deposition of conductive materials such as metals. In magnetron sputtering, a magnetic field confines electrons near the target surface, thereby enhancing ionization and deposition efficiency. The second method utilizes an alternating current (AC) at radio frequencies (RFs) to sustain the plasma, enabling the deposition of both conductive and insulating materials, albeit at lower rates compared to the DC method.

DC sputtering can be enhanced through magnetron technology to improve deposition efficiency and film quality. The selection of this method depends on the specific material requirements and the desired properties of the film [206]. The magnetron sputtering deposition technique utilizes magnetic fields beneath the target to confine electrons in the plasma near the target. This confinement increases the likelihood of ionizing collisions, thereby enhancing plasma density and sputtering effectiveness. Cooling water is employed to prevent excessive heating of the target. In the reactive sputtering process, additional gases, such as O_2_, are introduced into the chamber to facilitate the formation of oxides. Traditional methods use solid substrates, but emerging approaches involve sputtering onto liquids, where the substrate is replaced by a low-vapor-pressure liquid (such as silicone oil, ionic liquids, or polymers) [205]. The inherent properties of ionic liquids or polymers render them ideal for stabilizing the resulting NPs. This method allows for enhanced control over size, morphology, and composition. The liquid medium facilitates nucleation and growth, ultimately yielding stable colloidal solutions. This technique has been successfully applied to the preparation of AgNPs and CuNPs. Strong capping agents and functionalized liquids have enabled the production of NPs approximately 2 nm in diameter [204,205,206,207,208]. Sputtering onto liquids offers a green and versatile approach, enabling the synthesis of pure NPs with precise size and shape control.

#### 3.1.4. Spray Pyrolysis

**Figure 6:**
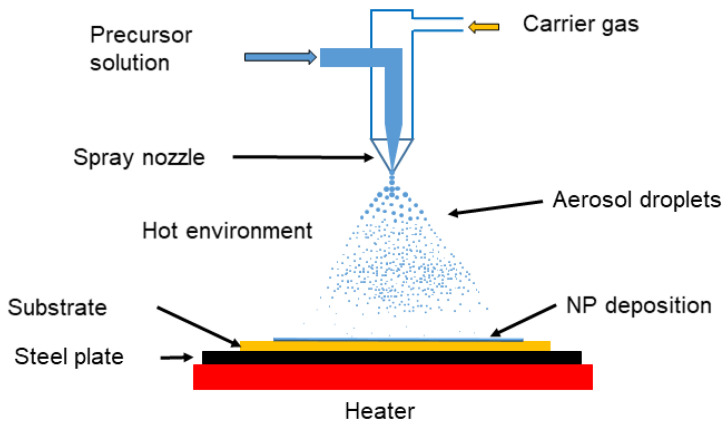
Schema of a spray pyrolysis in a hot chamber.

Spray pyrolysis (SP) entails atomizing a precursor metal-salt solution or suspension into micron-size aerosol droplets, which are transported by a carrier gas into a high-temperature furnace or heated environment, such as a flame, where solvent evaporation, solute precipitation, drying, and thermal decomposition occur. This sequence results in the formation of MNPs or MONPs, which are subsequently collected on a substrate (Figure 6).

Sometimes, additional heat treatment is required to optimize the crystallinity or morphology of the NPs [209]. The precursor solutions can be aqueous or organic and typically contain inorganic or metal–organic salts (e.g., chlorides, nitrates, carbonates), selected according to the desired final product [208,210,211]. Organic additives are often incorporated into the precursor solution to modify its properties or to influence the process, facilitating the formation of various nanostructures [209,212]. SP is considered a promising method for producing NPs with tailored characteristics. It is simple, cost-effective, and readily adaptable for industrial-scale production. Moreover, SP enables the synthesis of a broad range of nanomaterials, including metals, in a single step [213].

In ultrasonic SP, ultrasonic waves are applied to the precursor solution to generate micron-sized droplets. This technique is more efficient in terms of cost and operational stability compared to pneumatic and electrostatic nebulizers. However, it offers relatively low yield and is not suitable for high-viscosity liquids [209]. SP has been used to synthesize ZnONPs [214,215,216]. In another study, researchers used a pneumatic high-performance nebulizer with a methanolic solution of Zn(NO_3_)_2_ and a heating chamber maintained at 600–1000 °C to produce ZnO particles. The particle sizes obtained were 200 nm at 600 °C, 320 nm at 800 °C, and 400 nm at 1000 °C. The corresponding agglomerate sizes were 1.0 μm, 1.7 μm, and 2.3 μm, respectively [217].

#### 3.1.5. Electrospray

**Figure 7:**
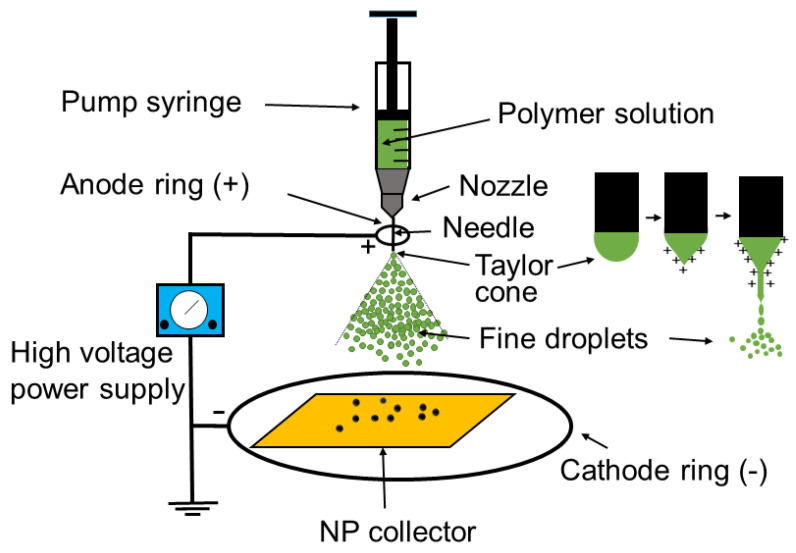
Schema of an electrospray device.

Electrospray entails applying a high voltage to a polymeric solution containing metal precursors, which is delivered by a syringe pump through a nozzle. A positively charged jet forms at the needle tip, where surface tension maintains a hemispherical droplet shape while Coulombic repulsion promotes droplet detachment. As charge builds, the droplet deforms into the characteristic Taylor cone, from which an aerosol of charged droplets is emitted and directed toward a grounded collector near the cathode. During travel, solvent evaporation occurs, resulting in NP deposition on the collector [218,219] (Figure 7).

This technique enables the fabrication of monodisperse NPs and offers small particle size, efficient drug encapsulation, high drug loading, controlled surface properties, multi-layer NP formation in a single step, rapid production, high purity, and broad material versatility. The design of the metal collector significantly influences the deposition pattern and alignment of the particles, thereby shaping their final morphology. Diverse NP architectures, including spherical, core–shell, hollow, cup-shaped, and porous morphologies, can be produced [220].

Physical synthesis methods offer advantages such as rapid processing, use of radiation (e.g., ionizing or microwave) as a reducing agent, and the absence of hazardous chemicals. However, these methods generally suffer from low yield, high energy consumption, and non-uniform particle distribution [221].

### 3.2. Bottom-Up Methods

In the bottom-up approach, nanostructures form via the sequential assembly of atoms or particles: supersaturation induces nucleation, followed by cluster growth and eventual NP formation [222]. This strategy encompasses a range of methodologies, including physical, chemical/electrochemical, and biological processes. A critical evaluation of the available methods reveals that each possesses a distinct set of advantages and drawbacks, with each offering its advantages and drawbacks.

#### 3.2.1. Physical Methods

##### Physical Vapor Deposition (PVD)

PVD is a broad class of vacuum deposition techniques used to produce thin films and NPs by physically transferring material from a source to a substrate in vapor form. Material is deposited onto a substrate, resulting in thin films or nanostructures. It includes several sub-techniques (e.g., thermal evaporation, electron- or ion-beam evaporation, sputtering, pulsed laser deposition, and arc vapor deposition). Depending on how NP synthesis is carried out, sputtering can be classified as either a bottom-up or a top-down process.

Evaporation/condensation of metal vapor is a subclass of PVD, also known as inert gas condensation. This method involves heating bulk pure metal to evaporate it into atoms, creating an aerosol within an inert gas atmosphere (He or Ar), followed by condensing the vapor into MNPs on a collector. This approach is considered a hybrid of top-down and bottom-up methodologies.

The PVD technique was applied in the preparation of CuNPs using an arc furnace at 5000 K to perform first the melting of the solid material and then the evaporation in an inert atmosphere made of a current of Ar as a carrier gas [223]. The CuNPs were in a size range from about 4 to 50 nm. The process has been applied to ZnNP synthesis [224]. The ZnNPs had a high purity (99.9%). However, the average particle size was found to be high (349 nm), which can be attributed to the evaporation temperature (approximately 900 °C). The synthesis of AgNPs in the size range of 9 to 32 nm has been accomplished [225]. This method uses pure metals, avoiding the need for chemical precursors and the presence of contaminants. The process parameters, evaporation temperature, and the inert gas flow significantly affect the particle mean size, size distribution, and shape.

#### 3.2.2. Chemical Methods

Chemical methods include those where chemical reactions among substances are involved to produce NPs. Some chemical methods include the reduction of metal ions from salt solutions in the presence of reducing agents and capping substances. But there are other processes.

##### Sol–Gel Method

The sol–gel method involves the transition of a molecular precursor-based system from a liquid sol (typically colloidal) into a solid gel phase. It proceeds via chemical reactions such as hydrolysis or alcoholysis, using heating and stirring of molecular precursors (e.g., metal alkoxides) dissolved in water, alcohol, other organic solvents, or mixtures thereof, followed by condensation to form a colloidal suspension (sol), which then evolves into a three-dimensional gel network [226]. Subsequent drying and thermal treatment yield NPs. The properties of the dried gel depend critically on the drying technique: thermal drying, supercritical drying, or freeze-drying produce xerogels, aerogels, or cryogels, respectively. This method has been demonstrated to be capable of synthesizing NPs exhibiting a range of structural and compositional features. It is effective for producing MONPs with high purity (>99.9%) and uniform sizes at relatively low temperatures (70–320 °C). This method is capable of producing two or more types of NPs simultaneously, meaning that alloy products are synthesized in one step by mixing two or more precursors of metal or metal oxide in certain ratios [226]. Metal oxide NPs, such as ZnONPs, have been synthesized by this process. These NPs can be useful in different areas [227].

##### Chemical Vapor Deposition (CVD)

In chemical vapor deposition (CVD), gaseous precursors undergo chemical reactions on a heated substrate, resulting in the deposition of a solid material, including MNPs. CVD may be viewed as a hybrid approach blending bottom-up and top-down features. Among the various CVD types, the most used are metal–organic CVD (MOCVD), microwave CVD (MWCVD) [228], and hot-wire CVD (HWCVD) [229].

**Figure 8:**
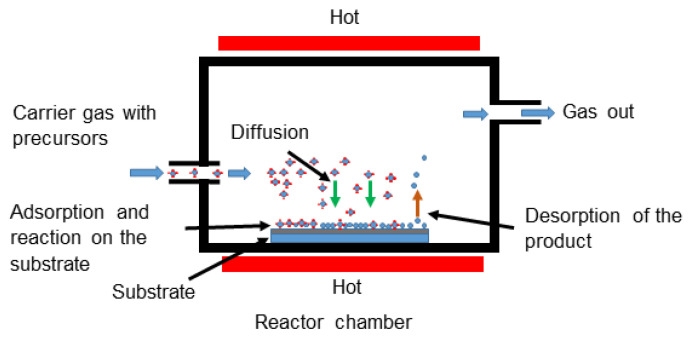
Schema of a chemical vapor deposition (CVD) device. Color arrows indicate the direction of movement (blue for the carrier gas, green: for precursors, and red for desorbed atoms).

The MOCVD process involves depositing a solid material following three consecutive steps: (a) introduction of the volatile precursor by the carrier gas into a heated reactor chamber; (b) diffusion and adsorption of precursor vapors on the heated substrate surface and the formation of intermediate products; and (c) decomposition of these products on the heated substrate followed by nucleation and growth of the solid layer/grains, the formation of volatile by-products, and their removal by the carrier gas [230] (Figure 8). The adsorbed atoms form a solid phase that is deposited onto the substrate. They can also grow and nucleate, producing NPs. MOCVD has been utilized in the fabrication of AgNPs.

MWCVD uses microwaves to heat the chamber. The primary factors influencing the metal deposition process include the temperature and the pressure within the chamber, in addition to the nature of the precursors [231].

##### Atomic Layer Deposition (ALD)

This technique bears a resemblance to MOCVD, yet it also exhibits notable distinctions. ALD is a cyclic process that utilizes alternating pulses of two or more gaseous precursors. These precursors are chemisorbed onto the substrate surface, thereby forming a film. This process also results in the removal of undesirable reaction by-products. The precursor pulses are separated by inert gas purges, which facilitate the removal of reaction byproducts and the suppression of unwanted gas-phase or pre-reactions. A diverse array of materials has been deposited using ALD, including nitrides, oxides, and certain metals. AgNP films have been deposited using direct liquid injection ALD with hexafluoroacetylacetone, silver(I), (1,5-cyclooctadiene), and propan-1-ol at a temperature between 123 and 128 °C [232].

Other chemical bottom-up approaches for NP synthesis include polyol methods, hydrothermal methods, co-precipitation methods, and microemulsion techniques [227].

##### Electrochemical Reduction

This method encompasses the use of an electrochemical cell to perform the reduction of metal ions to atoms. The apparatus typically includes an anode made of the metal target, a cathode (which can be made of glassy carbon or platinum), and an electrolyte solution containing the metal ions. Several factors influence the size and morphology of the MNPs, including the nature of the anode and cathode, the electrolyte, the temperature, and the voltage and current employed in the process [233]. Capping agents may be added to help aggregation control. This process has been used to synthesize AgNPs [233,234] and CuNPs [235] among others.

##### Chemical Reduction

Reduction may occur from the metal ions in a liquid solution when they are mixed with a suitable reagent that acts as a reducing agent. Usually, other substances that act as capping agents for the MNPs are also added. This method makes it possible to obtain MNPs both on the substrate surface and in the form of dispersed particles in colloidal systems, glasses, or polymers [230].

In the case of AgNPs, the salt commonly used is AgNO_3_, which readily dissolves in water. The reducing substances in question may be chemical compounds such as sodium borohydride (NaBH_4_) alone [236,237], which functions as both a reducing and a stabilizing agent. However, NaBH_4_ has been utilized in conjunction with other reagents in aqueous or organic solvents, including sodium dodecyl sulfate, chitosan, polyvinylpyrrolidone, trisodium citrate, dimethylformamide, and Tween 20 [238,239,240], among others. These reagents are typically dissolved in either an aqueous medium or an organic solvent. The reduction process transforms metal ions into metal atoms, and the citrate molecules cap the AgNPs, thereby preventing aggregation and promoting stability. The pH level is modulated through the incorporation of appropriate bases, such as ammonia, into the solution. In certain instances, the application of heat and agitation has been employed as a means to enhance the reduction of metal ions. A dark brown colloidal suspension of AgNPs is obtained. The suspension is then subjected to a process of centrifugation and water rinsing, a technique employed to eliminate the superfluous reagents and substances that are byproducts of the reaction. The NPs are then stored until use. In alternative processes, the reaction mixture is maintained at a low temperature in an ice bath to reduce the reaction rate and enhance the reproducibility of the process [240].

#### 3.2.3. Biological or “Green” Synthesis

**Figure 9:**
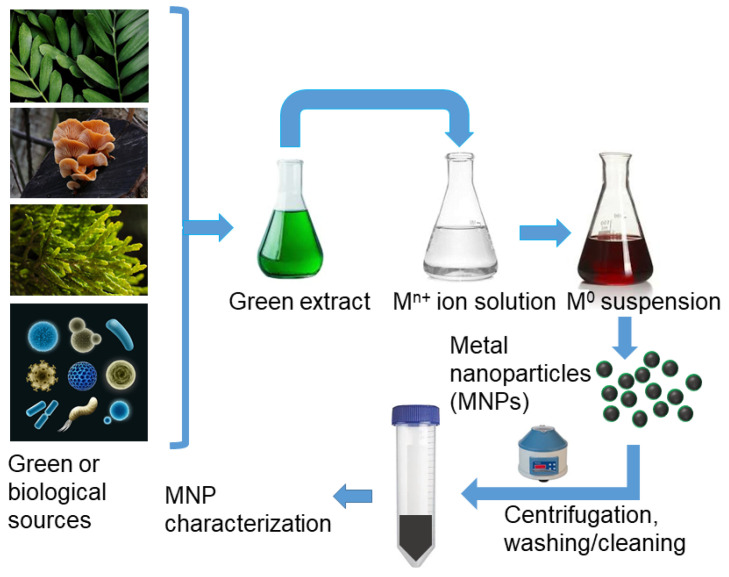
Schema of a generalized procedure for green or biological synthesis of metal nanoparticles.

Biological synthesis is the process of reducing metal ions by utilizing natural specimens, such as extracts from terrestrial plant parts (e.g., leaves, fruits, roots, rhizomes, whole plants), bacteria, fungi, yeasts, algae, or honey. These extracts are also employed to cap and modify the surface of the synthesized NPs [241]. These procedures are the most cost-effective and environmentally friendly methods, known as “green synthesis” or biosynthesis, and are the subject of most publications on MNPs [223,242,243,244,245,246]. Many types of MNPs and MONPs have been synthesized; however, in the context of the present review, we are mainly interested in those made of Ag, Cu, Fe, CuO, Fe_3_O_4_, MgO, and ZnO [247]. A schematic of the generalized procedures to prepare MNPs through green synthesis is shown in Figure 9.

The organic material from biological species acts as a capping and stabilizing agent for the produced MNPs or MONPs. Among the fungi used to make extracts to reduce the solutions of metal salts, it is noteworthy to mention species of *Fusarium*, *Penicillium*, *Aspergillus*, or *Talaromyces.*

The pH and temperature affect the size and texture of the NPs produced using green technology. Therefore, regulating the pH and temperature of the solution media can control the NP size. Higher temperatures result in faster synthesis. NP production increases with size, which is also temperature-regulated [248]. Green synthesis has certain advantages compared to chemical and physical methods: it is claimed to be non-toxic, pollution-free, environmentally friendly, economical, and more sustainable [249].

Thus, green-synthesized NPs are attractive for many localized or specific applications such as eco-friendly antimicrobials, certain medical uses, or applications in developing countries. However, their general usage on a global industrial scale is limited by batch-to-batch variability (inconsistent size, shape, and purity), unpredictable surface chemistry, and a lack of standard protocols. They are often application-specific rather than universally applicable.

## 4. Antifungal Mechanisms of Metal Nanoparticles

**Figure 10:**
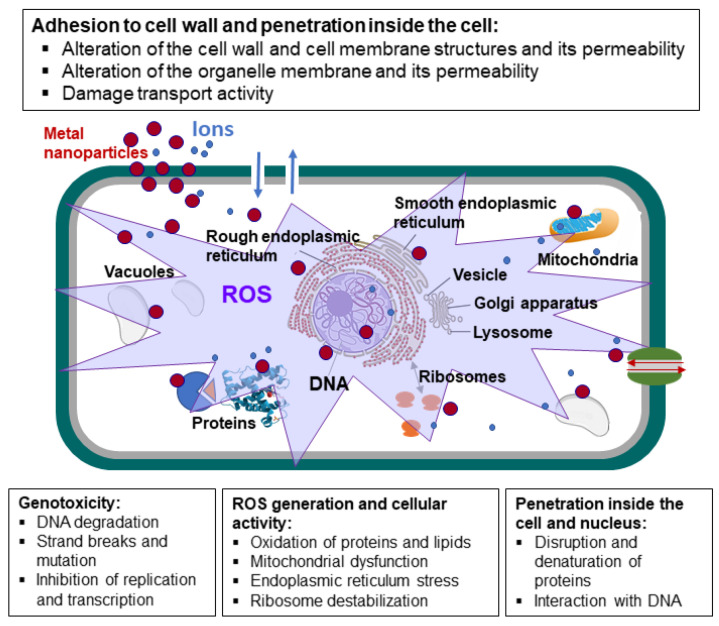
Activity of metal NPs against the fungal cell.

A significant number of studies have investigated this domain of nanotechnology; however, the mechanisms of action involved in MNPs and MONPs used as antifungal agents are not fully elucidated. The ideal nano-fungicide would demonstrate equivalent or superior activity compared to that of the bulk metal at relatively lower concentrations. As illustrated in Figure 10, at the cellular level, MNPs have been observed to show a variety of effects. The antifungal activity of MNPs can be attributed to the following events:

(a) The fungal cell wall undergoes changes and damage, including surface shrinkage, cell aggregation, pit and pore formation, and general deformation. Internalization of the NPs into fungal cells occurs through three principal mechanisms: (i) direct penetration of NPs through the cell wall, (ii) specific receptor-mediated adsorption followed by internalization, and (iii) uptake through ion transport proteins. During adsorption, NPs can embed within fungal cell walls, which induces morphological and functional changes [250]. Additionally, the NPs can release metal ions from the extracellular space. These ions can enter the fungal cell, thereby disrupting its biological processes [191].
(b) The metal ions will contribute to the formation of NPs intracellularly through reduction processes by cellular organic compounds [251]. The fungal cell wall plays a critical role in various processes, including fungal growth and defense, morphogenesis, and biofilm formation. The primary functions of the cell include buffering fluctuations in osmotic pressure, sensing external stimuli, and protecting against detrimental conditions such as dryness, heat, and toxic molecules. The cell wall plays a pivotal role in the pathogenicity and virulence of pathogenic fungi, aiding in their invasion while protecting the fungus from host defense mechanisms [191]. The cell wall, a structural component of cells, appears to be a rigid structure; nevertheless, it is dynamic and is subject to constant remodeling due to several factors. These include fungal growth, which encompasses processes such as expansion, sporulation, and branching, as well as environmental challenges. The process of binary fission, also known as the expansion of hyphae, is contingent upon the concurrent activity of anabolic and catabolic enzymes. Therefore, based on the composition of the fungal cell wall, it can be concluded that the structure provides an optimal target for antifungal MNPs [191].
(c) The disruption of the cell membrane is a consequence of the interaction between MNPs and the fungal cell membrane, leading to structural damage. As Slavin’s hypothesis states [191], positively charged metal-based NPs establish a robust bond with cell membranes, thereby increasing membrane permeability. This process facilitates the diffusion of essential ions and molecules, ultimately resulting in the demise of fungal cells.
(d) The internal membranes are distorted, and there is an alteration in the organelle disposition. This phenomenon is evidenced by an increase in the intracellular vesicle and vacuole count and a decrease in cytoplasmic content. The loss of intracellular structure results in the accumulation of cytoplasm within the cell, accompanied by an apparent absence of organelles. This complicates the process of distinguishing between the cytoplasm, plasma membrane, and cell wall boundaries after exposure to NPs.
(e) The underlying mechanism of this complication is the alteration of these structures by NPs, thereby obscuring the boundaries [185,190,251,252,253]. The generation of reactive oxygen species (ROS) is an inherent process within the human body. In the cell, the presence of metal ions or NPs has been observed to trigger the generation of ROS, which includes superoxide radicals and hydrogen peroxide. It has been demonstrated that ROS play a critical role in the antifungal activity mechanism of NPs. These substances have been demonstrated to induce oxidative stress in fungal cells. The oxidative stress can suppress the antioxidant defense mechanism of the fungus against ROS. Subsequently, these metal ions have been demonstrated to interact with cellular structures, thereby inducing damage to cellular components such as proteins, lipids, and DNA, which ultimately results in cell death [254,255].
(f) Interaction with the fungal DNA is indicated herein. MNPs have been observed to penetrate fungal cells and interact with the DNA. The NPs can bind to the genetic material (which is negatively charged), resulting in structural damage, DNA fragmentation, or hindrance to DNA replication and transcription. This, in turn, disrupts the ability of the fungi to proliferate [191]. In addition, the NPs can induce mitochondrial DNA fragmentation, ribosome depolymerization, cellular dysfunction, and apoptosis [191]. The inhibition of enzyme activity is a consequence of the presence of MNPs within the fungal cell, thereby interfering with the function of the enzymes contained within. MNPs can bind to sulfhydryl groups on enzymes, thereby inhibiting their normal function and resulting in metabolic disruptions [118,256]. Metal ions have been observed to form strong coordination bonds with N, O, or S atoms. These atoms are found in abundance in organic compounds and biomolecules. Given the non-specific nature of the bond between metal ions and biomolecules, metal-based NPs typically demonstrate a broad spectrum of activities. It has been demonstrated that AgNPs exhibit reduced chemical reactivity in comparison to Ag^+^ ions. The interaction of Ag^+^ ions with a diverse array of biomolecules within the cell has been well documented, including nucleic acids, components of the cell wall, sulfhydryl groups of metabolic enzymes, and sulfur-containing cell components [257].
(g) Synergistic effects. MNPs may exhibit enhanced antifungal activity when utilized in conjunction with other antifungal agents, suggesting a potential for synergistic interactions that can amplify the antifungal effect [258,259].

## 5. Main Metal and Metal Oxide NPs Tested Against Toxigenic Fungi and Mycotoxin Production

### 5.1. Silver Nanoparticles

In soils, silver is predominantly found in the form of sulfides, often associated with iron, lead, or tellurides. It has been observed that this phenomenon is also connected with gold. The Ag^+^ ion is a prevalent constituent of surface waters, where it is present as sulfide, bicarbonate, or sulfate salts, or through adsorption onto organic or inorganic materials. Furthermore, the presence of Ag has been identified in conjunction with more complex ions, particularly in association with chlorides and sulfates. A considerable proportion of these forms demonstrate limited or sparingly soluble characteristics, thereby constraining their availability to biological organisms. Silver has gained a reputation for its biocidal properties [260]. It exhibits bactericidal, fungicidal, and virucidal properties irrespective of its form, including Ag^+^ ions, silver complexes, metallic silver (Ag^0^), and AgNPs. Due to their elevated biological activity, silver compounds are extensively utilized across a broad array of disciplines, particularly within the fields of biology and medicine. The elevated surface-to-volume ratio of AgNPs contributes to their enhanced biological efficacy in comparison to Ag^0^. Nonetheless, the reactivity of AgNPs is lower than that of Ag^+^ ions. These ions interact with a variety of biomolecules within a cell (e.g., nucleic acids, cell wall components, and sulfhydryl groups of metabolic enzymes) [261]. Consequently, the toxicity of the Ag^+^ ions is found to be higher than that of the AgNPs. It is noteworthy that AgNPs function as a source of silver ions. AgNPs exhibit a propensity for oxidative dissolution, a process that instigates the continuous release of Ag^+^ ions. The rate of Ag^+^ ion leaching from AgNPs is contingent upon external conditions and the physicochemical properties of the AgNPs [262].

Due to their proven efficacy in medical applications [263], the impact of AgNPs on various biological systems is currently under extensive research. This knowledge can contribute to the development of new methodologies in other scientific disciplines, including the control of fungal and mycotoxin contamination in food production. Some properties inherent to AgNPs must be taken into consideration when assessing their potential application in the domain of food technology. It has been established that smaller AgNPs exhibit heightened toxicity in comparison to their larger counterparts [186]. This phenomenon can be attributed primarily to the enhanced permeability of smaller AgNPs, which allows for easier penetration into cellular structures. In addition, smaller AgNPs demonstrate increased sensitivity to oxidative dissolution, leading to accelerated silver ion generation over larger AgNPs within shorter time frames [264]. The morphology of AgNP is a critical factor in regulating its biocidal characteristics. In many cases, molecules that regulate the morphology of the particles are deposited on the surface of the AgNPs. These molecules have also been demonstrated to exhibit biological activity. Consequently, the surface chemistry of AgNPs emerges as a pivotal factor in determining the biological activity of the entire system [265].

Therefore, in addition to size and shape, it is imperative to examine the role of chemicals utilized as reducing and stabilizing agents of AgNPs, as they significantly in-fluence the modeling of biological activity [266]. As demonstrated by Oćwieja and Barbasz [267], the type of reducing and stabilizing agents on AgNPs can amplify or re-duce the Ag+ ion release, or intensify or reduce the AgNP penetration through biological membranes. Therefore, the use of AgNP-stabilizing agents that possess biocidal proper-ties can cause synergistic effects, thus amplifying the silver toxicity [265]. Typically, AgNPs stabilized by inorganic anions exhibit a negative charge. Conversely, AgNPs en-veloped by organic compounds possessing moieties capable of protonation and depro-tonation can assume a negative or positive charge [267].

The surface charge of AgNPs has been demonstrated to influence the formation of a protein corona around NPs [268] and their subsequent interactions with the cell membrane [265,269]. It has been observed that the efficacy of different types of AgNPs, obtained by the reduction of silver ions with NaBH_4_ in the presence of trisodium citrate or cysteamine hydrochloride, varies when tested against *Fusarium avenaceum* and *Fusarium equiseti* [270]. The morphology of the two types of AgNPs was quasi-spherical. The citrate-stabilized AgNPs exhibited an average size of 15 ± 4 nm and were found to be negatively charged. In the context of this study, smaller cysteamine-capped AgNPs (12 ± 4 nm) were found to exhibit a positive surface charge and a higher silver ion release profile. It was observed that cysteamine-capped AgNPs caused damage to the conidia membranes and penetrated the cells of both fungal species, while citrate-stabilized AgNPs were deposited on their surface. A consensus emerged from the research that the cysteamine-capped AgNPs exhibited superior performance in comparison to the citrate-capped AgNPs. Consequently, ascertaining the precise chemical composition of the NP is imperative for its utilization in the domain of food technology. Despite the apparent popularity of plant extracts, bacteria, and fungi as reducing and stabilizing agents in the biosynthesis of AgNPs, this practice introduces uncertainty about the final composition of the NPs. This characteristic presents a notable disadvantage when considering their application in the food industry. Consequently, the employment of defined chemical compounds in the chemical synthesis of NPs may prove to be more advantageous than the utilization of complex reducing agents. The selective action of AgNPs against toxigenic fungi and mycotoxin production is highly desired; therefore, the design of AgNPs with selective antifungal activity and an acceptable size, shape, and charge to preserve the health of consumers and ecosystems in general is a priority objective for its possible implementation in food technology.

It has been documented that AgNPs exhibit a wide range of antimicrobial activity against various microorganisms [271]. The antimicrobial action of AgNPs has been reported on a variety of pathogens, including bacteria [240,272], fungi [191,270,273], and viruses [274]. Despite the paucity of research on the antifungal activity of MNPs on toxigenic fungi affecting crops in pre- and post-harvest, and their effect on mycotoxin biosynthesis, the existing literature on this subject is predominantly focused on AgNPs [172,238,275].

The impact of engineered AgNPs, produced through biological, chemical, and physical synthesis, as well as commercial formulations, has been examined concerning various fungal species classified as mycotoxin producers. However, the majority of these studies have focused exclusively on the impact of these NPs on the regulation of fungal growth.

Table 1 presents a compendium of recent publications that, to varying extents and with varying degrees of experimental rigor, examine the antifungal properties of AgNPs against species designated as mycotoxin producers. It is important to note that the utilization of these strains in the aforementioned studies does not inherently imply that they possess the characteristic of producing mycotoxins.

Table 2 enumerates the works in Table 1 where, in addition to the effect of AgNPs on fungal growth, the effects on mycotoxin production are also studied. Consequently, in such cases, these fungi were classified as mycotoxin-producing strains. These are the following studies [238,275,276,277,278,279,280,281,282,283,284,285].

As shown in Table 1 and Table 2, the majority of the examined AgNPs exhibit a spherical morphology, which may be attributed to the relative ease with which this shape can be synthesized. In terms of size, the focus of the majority of studies has been on suspensions of polydisperse AgNPs. This approach precludes the possibility of conducting a thorough investigation into the relationship between the size of the NPs and their antifungal activity. The studies illustrated in Table 1 and Table 2 exhibit a consistent pattern. The findings indicate that as the concentration of AgNPs increases, there is a concomitant decline in fungal growth, the number of viable spores, and the production of mycotoxins. This phenomenon occurs irrespective of other variables, including the synthesis method, the size and shape of the NPs, and the specific fungal species utilized in the testing. Furthermore, the emergence of resistant strains following the treatment regimen was not observed.

**Table 1:** Antifungal effect of silver nanoparticles on toxigenic fungi.

Nanoparticle Properties			Antifungal Properties			
Synthesis Method	Size (nm)	Shape	Fungal Species	Methodology	Growth Reduction/(%)/Treatment	Ref.
Chemical	14–100 (30)	Spherical	*F. graminearum*, *F. culmorum*, *F. sporotrichioides*, *F. langsethiae*, *F. poae*, *F. oxysporum*, *F. proliferatum*, *F. verticillioides*	Medium: Maize-based medium. Inoculum: From a spore suspension (1 × 10^5^ spores/mL) previously treated with AgNPs for 2, 10, 20, and 30 h. AgNP concentration: 2, 5, 10, 15, 30, and 45 ppm. Incubation: 28–25 °C, 10 days. Fungal growth record: Spore viability (sv) and colony diameter (cd).	100% (sv, cd)/2.0 ppm (30 h), 100% (sv, cd)/15.0 ppm (30 h), 100% (sv, cd)/10.0 ppm (30 h), 100% (sv, cd)/2.0 ppm (30 h), 100% (sv, cd)/2.0 ppm (30 h), 100% (sv, cd)/45.0 ppm (30 h), 100% (sv, cd)/30.0 ppm (30 h), 100% (sv, cd)/30.0 ppm (30 h). For each species, respectively	[275]
Chemical	14–100 (30)	Spherical	*A. flavus*, *A. parasiticus*, *A. carbonarius*, *A. niger*, *A. ochraceus*, *A. steynii*, *A. westerdijkiae*, *P. verrucosum*	Medium: Maize-based medium. Inoculum: From a spore suspension (1 × 10^5^ spores/mL) previously treated with AgNPs for 2, 10, 20, and 30 h. AgNP concentration: 2, 5, 10, 15, 30, and 45 ppm. Incubation: 20–37 °C, 10 days. Fungal growth record: Spore viability (sv) and colony diameter (cd).	100% (sv, cd)/15.0 ppm (30 h), 100% (sv, cd)/30.0 ppm (30 h), 100% (sv, cd)/10.0 ppm (30 h), 100% (sv, cd)/15.0 ppm (30 h), 100% (sv, cd)/5.0 ppm (30 h), 100% (sv, cd)/5.0 ppm (30 h), 100% (sv, cd)/5.0 ppm (30 h), 100% (sv, cd)/15.0 ppm (30 h). For each species, respectively	[238]
Chemical	20	Spherical	*A. parasiticus*	Medium: Potato Dextrose Agar (PDA), Czapeck Dox Agar (CZA), Potato Dextrose Broth (PDB), and Czapeck Dox Broth (CZB). Inoculum: From a spore suspension (1 × 10^5^ spores/mL) previously treated with AgNPs for 2, 10, 20, and 30 h. AgNP concentration: 25, 50, 100, and 200 ppm. Incubation: 28 °C, 7 days. Fungal growth record: Colony diameter (cd) and mycelium weight (mw).	34% and 41% (cd)/200 ppm (in PDA and CZA, respectively) 92% (mw)/250 ppm (in both PDB and CZB)	[276]
Chemical	∼7.5	Spherical	*Gibberella* *fujikuroi*	Medium: PDA. Inoculum: From a spore suspension (7 × 10^4^ spores/mL) previously treated with AgNPs for 1, 10, and 20 min. AgNP concentration: 0.00015, 0.0015, 0.015, 0.15, 1.5, 15, and 150 ppm. Incubation: 25 °C, 3 days. Fungal growth record: CFU. Medium: PDA. Inoculum: Rice seeds previously dipped in a spore suspension (5 × 10^5^ spores/mL). AgNP concentration: Solution of 150 ppm (rice seeds previously infected with fungal spores are immersed in this solution for 1/6, 1/3, 1/2, 1, 3, 6, or 24 h). Incubation: 25 °C, 3 days. Fungal growth record: CFU (on seed surface).	50% CFU/0.15–1.5 ppm (1 min) 100% CFU/≥ 1.5 ppm (1 min) 96.2% CFU/150 ppm (≥10 min)	[286]
Chemical	25 ± 2.6	Spherical	*P. digitatum*, *P. italicum*	Medium: PDA. Inoculum: From a spore suspension (1 × 10^6^ spores/mL) previously treated with AgNPs for 24 h. AgNP concentration: 1–10 ppm. Incubation: 22 ± 1 °C, 4 days. Fungal growth record: CFU. Medium: Lemons. Inoculum: From a spore suspension (1 × 10^6^ spores/mL) previously treated with AgNPs for 24 h. Concentration of AgNPs: 1–10 ppm. Incubation: 20 °C, 95% RH, 5–14 days. Fungal growth record: Lemon disease incidence.	100% CFU/10 ppm (24 h) 100%/10 ppm/24 h)	[287]
Chemical	17 ± 1.5	Spherical	*F. verticillioides*	Medium: Nutrient Broth (according to CLSI for filamentous fungi). Inoculum: From a spore suspension (2 × 10^4^ spores/mL). AgNP concentration: 5–200 ppm. Incubation: 25 °C, 48 h. Fungal growth record: MIC.	100% (MIC)/75 ppm	[285]
Chemical	12 ± 4		*F. avenaceum*, *F. equiseti*	Medium: PDA. Inoculum: From a spore suspension (—) previously treated with AgNPs for 24–240 h. AgNP concentration: 2.5, 5, and 10 ppm. Incubation: 21 °C, 168 h. Fungal growth record: Colony diameter.	∼25%/10 ppm (168 h) ∼43%/10 ppm (168 h) For each species, respectively	[270]
Biological (*Penicillium chrysogenum*, *Fusarium chlamydosporum*)	9–17.5 6–26	Spherical Spherical	*A. flavus*, *A. ochraceus*	Medium: CZA. Inoculum: From a spore suspension (2 × 10^6^ spores/mL). AgNP concentration: 10–50 ppm. Incubation: 30 °C, 16 h. Fungal growth record: Spore germination.	100%/45–48 ppm 100%/47–51 ppm For each species, respectively	[284]
Biological (*Aspergillus terreus*)	5–30	Spherical	*A. flavus* (5 strains)	Medium: PDA. Inoculum: Agar plugs (6 mm) from a fungal culture. AgNP concentration: 50, 100, and 150 ppm. Incubation: 25 ± 2 °C, 7 days. Fungal growth record: Colony diameter.	8.9–21.1%/50 ppm 43.3–54.8%/100 ppm 71.1–86.3%/150 ppm	[277]
Biological (*Fusarium oxysporum*)	93 ± 11	Spherical	*A. flavus*, *A. melleus*, *A. nomius*, *A. ochraceus*, *A. parasiticus*	Medium: Nutrient Broth and Sabouraud Dextrose Agar (SDA). Inoculum: From a spore suspension (1 × 10^5^ spores/mL). AgNP concentration: 0.26–135 ppm. Incubation: 30 °C, 72 h. Fungal growth record: MIC and MFC (<3 colonies/plate).	MIC and MFC/8 and 64 ppm, MIC and MFC/4 and 16 ppm, MIC and MFC/8 and 32 ppm, MIC and MFC/4 and 16 ppm, MIC and MFC/8 and 32 ppm For each species, respectively	[288]
Biological (*Malva parviflora* L.)	50.6	Spherical	*F. solani*, *F. oxysporum*, *A. alternata*	Medium: PDA. Inoculum: Agar plugs (6 mm) from a fungal culture. AgNP concentration: 50,000 ppm. Incubation: 25 ± 2 °C, 7–9 days. Fungal growth record: Colony diameter.	81.1%/50,000 ppm, 80.7%/50,000 ppm, 83.0%/50,000 ppm, For each species, respectively	[289]
Biological (Honeybee)	—	Cubic	*F. avenaceum*, *F. culmorum*, *F. graminearum*, *F. oxysporum*, *F. poae*, *F. proliferatum*, *F. pseudograminearum*, *F. sambucinum*, *F. semitectum*, *F. sporotrichioides*, *F. verticillioides*	Medium: PDA. Inoculum: From a spore suspension (2.5 × 10^3^ spores/mL) (well diffusion method). AgNP concentration: 5, 25, 50, 75, and 100 ppm. Incubation: 25 ± 2 °C, 7 days. Fungal growth record: Growth reduction (%).	3.3 ± 0.0–31.2 ± 0.0%/5–100 ppm, 2.2 ± 0.0–36.8 ± 0.3%/5–100 ppm, 2.2 ± 0.0–25.8 ± 0.1%/5–100 ppm, 1.1 ± 0.0–16.8 ± 0.2%/5–100 ppm, 2.2 ± 0.1–26.8 ± 0.2%/5–100 ppm, 2.2 ± 0.1–23.4 ± 0.2%/5–100 ppm, 4.4 ± 0.2–27.8 ± 0.1%/5–100 ppm, 4.4 ± 0.2–31.2 ± 0.2%/5–100 ppm, 3.3 ± 0.1–25.6 ± 0.2%/5–100 ppm, 2.2 ± 0.1–18.9 ± 0.3%/5–100 ppm, 2.2 ± 0.0–28.9 ±0.4%/5–100 ppm, For each species, respectively	[278]
Biological (*Chaetomium globosum*)	11–14	Spherical	*F. oxysporum*	Medium: PDA, Corn Meal Agar (CMA), and Malt Extract Agar (MEA). Inoculum: —. AgNP concentration: 50, 100, and 500 ppm. Incubation: 28–30 °C, 7 days. Fungal growth record: Colony diameter and CFU. Medium: Tomato seeds. Inoculum: —. AgNP concentration: 500 ppm. Incubation: 28–30 °C, 4 h. Fungal growth record: Seedlings wilt.	88% (cd)/500 ppm 100% (CFU)/500 ppm Complete inhibition of seedlings wilt/500 ppm.	[290]
Biological (*Epifocal nigrum*)	1–22	Spherical	*A. flavus*	Medium: Nutrient Broth (documents M27-A, M38-A). Inoculum: From a spore suspension (2.5–5 ×10^3^ spores/mL). AgNP concentration: 0.125–64 ppm. Incubation: 25 °C, 48 h. Fungal growth record: MIC (reduction of fungal growth by 80%).	80%/5 ppm	[291]
Biological (*Tropaeolum majus*)	—	Spherical	*A. niger*	Medium: Nutrient Broth + resazurin indicator solution. Inoculum: From a spore suspension (5 × 10^6^ spores/mL). AgNP concentration: 1–500 ppm. Incubation: —. Fungal growth record: At a glance.	100%/≥ 31.2 ppm	[292]
Biological (*Trichoderma harzianum*)	—	—	*F. moniliforme*	Medium: PDA, CZA, Yeast Dextrose Agar (YES). Water Agar (WA). Inoculum: Agar plugs (5 mm) from a fungal culture. Concentrations of AgNPs: 0–800 ppm. Incubation: 25 ± 2 °C, 5 days. Fungal growth record: Colony diameter.	0–60.04%/0–800 ppm	[293]
Biological (*Geranium* leaves)	38.5 ± 18.5	Spherical	*F. oxysporum* f. sp. *lycopersici*	Medium: PDA. Inoculum: Agar plugs (6 mm) from a fungal culture. AgNP concentration: 10, 20, 40, 75, and 150 ppm. Incubation: 27 ± 2 °C, 7 days. Fungal growth record: Colony diameter. Medium: PDB. Inoculum: From a spore suspension (1 × 10^4^ spores/mL). AgNP concentration: 10, 20, 40, 75, and 150 ppm. Incubation: 27 ± 2 °C, 72 h. Fungal growth record: MIC. Medium: Tomatoes. Inoculum: From a spore suspension (1.5 × 10^5^ spores/mL). AgNP concentration: 10, 20, 40, 75, and 150 ppm. Incubation: 27 ± 2 °C, 6 days. Fungal growth record: Tomato disease incidence.	94.6%/150 ppm 100%/75 ppm 100%/10–100 ppm	[294]
Biological (*Althaea officinalis*, *Thymus vulgaris*, *Mentha pulegium*)	50 50 50	Spherical Spherical Spherical	*A. flavus*, *P. chrysogenum*	Medium: PDA. Inoculum: From a spore suspension (1 × 10^5^ spores/mL, disk diffusion method). AgNP concentration: suspension 1 mM. Incubation: 26–27 °C, 60 h. Fungal growth record: Diameter inhibition zone.	35–36 mm/1 mM 36–37 mm/1 mM For each species, respectively	[295]
Biological (*Arthroderma fulvum*)	15.5 ± 2.5	Spherical	*A. flavus*, *A. terreus*, *F. solani*, *F. moniliforme*, *F. oxysporum*	Medium: Nutrient broth (document M38-A2). Inoculum: From a spore suspension (1.0–2.0 × 10^4^ spores/mL). AgNP concentration: 0.125–64 ppm. Incubation: 28 °C, 48 h. Fungal growth record: MIC (reduction of fungal growth by 80%).	80%/2.00 ppm, 80%/1.00 ppm, 80%/2.00 ppm, 80%/4.00 ppm, 80%/2.00 ppm, For each species, respectively	[296]
Biological (*Amaranthus retroflexus*)	10–32	Spherical	*A. alternata*, *F. oxysporum*	Medium: PDA. Inoculum: Agar plugs (5 mm) from a fungal culture. AgNP concentration: 50, 100, 200, and 400 ppm. Incubation: 25 °C, 5 days. Fungal growth record: Colony diameter.	50%/337.09 ± 19.72 ppm, 50%/328.05 ± 13.29 ppm, For each species, respectively	[297]
Biological (*Phyllanthus urinaria*, *Pouzolzia zeylanica*, *Scoparia dulcis*)	28.3 26.7 <26.7	Spherical Spherical, hexagonal, triangle, etc. Spherical	*A. niger*, *A. flavus*, *F. oxysporum*	Medium: PDA. Inoculum: Colonies from fungal cultures. AgNP concentration: 15, 30, and 45 ppm. Incubation: Room temperature, 4 days. Fungal growth record: Colony diameter.	40–60%/45 ppm Depending on the fungal species	[298]
Biological (*Aspergillus terreus*)	15–29	Spherical	*F. solani*, *A. alternata*, *A. flavus*, *A. ochraceus*	Medium: CZA. Inoculum: –. AgNP concentration: 1 mM, 2 mM, 5 mM, 10 mM, and 20 mM (wells, 7 mm). Incubation: 28 °C, 3–5 days. Fungal growth record: Diameter inhibition zone.	3–13 mm/0.84–1.68 ppm	[299]
Biological (*Trichoderma longibrachiatum*)	5–30 (10)	Spherical	*A. alternata*, *F. verticillioides*, *F. moniliforme*, *A. flavus*, *P. brevicompactum*	Medium: PDA. Inoculum: From a spore suspension treated with 0.5 mM AgNPs. AgNP concentration: 0.5 mM. Incubation: 28 °C, 24 h. Fungal growth record: CFU.	93.0%/0.5 mM, 96.4%/0.5 mM, 93.6%/0.5 mM, 86.7%/0.5 mM, 92.9%/0.5 mM, For each species, respectively	[300]
Biological (*Momordica charantia*, *Psidium guajava*)	5–29 (17) 5–53 (25.7)	Spherical Spherical	*A. niger*, *A. flavus*, *F. oxysporum*	Medium: PDA. Inoculum: –. AgNP concentration: 20 and 40 ppm. Incubation: 30 °C, 24–96 h. Fungal growth record: Colony diameter.	<50%/40 ppm (96 h)	[301]
Biological (*Cryptococcus laurentii*, *Rhodotorula glutinis*)	15–400	Spherical	*P. expansum*, *A. niger*, *Alternaria* sp.	Medium: PDA. Inoculum: From a spore suspension (2.0 × 10^6^ spores/mL) (wells of 3 mm). AgNP concentration: 3 ppm. Incubation: 28 ± 4 °C, 7 days. Fungal growth record: Diameter inhibition zone.	11.1 ± 1.4 mm/3 ppm, 14.8 ± 2.2 mm/3 ppm, 11.3 ± 1.6 mm/3 ppm, For each species, respectively	[302]
Biological (*Pseudomonas poae*)	19.8–44.9	Spherical	*F. graminearum*	Medium: PDA and PDB. Inoculum: Agar plugs (5 mm) from a fungal culture. AgNP concentration: 5, 10, 15, and 20 ppm. Incubation: 28 °C, 5 days. Fungal growth record: Colony diameter in PDA and mycelium growth in PDB.	PDA: 45.56%/5 ppm 62.22%/10 ppm 72.78%/15 ppm 80.56%/20 ppm PDB: 48.56%/5 ppm 65.11%/10 ppm 75.50%/15 ppm 85.78%/20 ppm	[279]
Biological (*Alternaria* sp.)	3–10	Spherical	*F. oxysporum*, *F. moniliforme*, *F. tricinctum*, *Alternaria* sp.	Medium: PDA. AgNP concentration: 1000 ppm (25, 50, and 100 µL (wells 8 mm). Inoculum: —. Incubation: 28 ± 1 °C, 5 days. Fungal growth record: Diameter inhibition zone.	14.7–21.3 mm/1000 ppm, 9–21.6 mm/1000 ppm, 11–21.2 mm/1000 ppm, 17.1–21.6 mm/1000 ppm, For each species, respectively	[303]
Biological (*Penicillium verrucosum*)	10–12	Spherical	*A. flavus*, *F. chlamydosporum*	Medium: PDA. Inoculum: Agar plugs (3 mm) from a fungal culture. AgNP concentration: 50, 100, 150, and 200 ppm. Incubation: 27 ± 2 °C, 7 days. Fungal growth record: Colony diameter.	59.13%/200 ppm, 56.67%/200 ppm, For each species, respectively	[304]
Biological (*Rhizoctonia solani*, *Cladosporium cladosporioides*)	80–100	Spherical	*A. flavus*, *P. citrinum*, *F. oxysporum*	Medium: Sabouraud Dextrose Agar (SDA). Inoculum: —. AgNP concentration: 5000, 10,000, 15,000 ppm (wells 5 mm). Incubation: —. Fungal growth record: Diameter inhibition zone.	15–21 mm/5000–15,000 ppm, 8–17 mm/5000–15,000 ppm, 8.33–13.33/5000–15,000 ppm, For each species, respectively	[305]
Biological (*Trigonella* *foenum-graecum*)	20–25	Spherical and cubic	*A. alternata*	Medium: PDA. Concentration of AgNPs: 100 ppm. Inoculum: —. Incubation: —. Fungal growth record: Colony diameter.	40–50%/100 ppm	[306]
Biological (*Nigrospora oryzae*)	3–13	Spherical	*F. sporotrichioides*, *F. oxysporum*, *F. moniliforme*, *F. solani*, *F. anthophilium*	Medium: CZA and PDA. Inoculum: Agar plugs (4 mm) from a fungal culture. AgNP concentration: 50, 100, 150, and 200 ppm. Incubation. 28 ± 2 °C, 5 days. Fungal growth record: Colony diameter.	50–70%/200 ppm Depending on the species	[307]
Biological (*Allium cepa*, *Zingiber officinale*, *Allium sativum*)	1–9 1–6 2–10	Spherical	*F. graminearum*, *F. avenaceum*, *F. culmorum*	Medium: PDB. Inoculum: —. AgNP concentration: 10, 30, 50, 70, 90, 110, 130, and 150 ppm. Incubation: 28 °C, 2 days. Fungal growth record: MIC.	100%/90–110 ppm, 100%/90–110 ppm, 100%/110 ppm, For each species, respectively	[308]
Biological (Honey)	9.9	Spherical	*A. parasiticus*, *A. ochraceus*	Medium: PDA. Inoculum: From a spore suspension (1 × 10^6^ spores/mL). AgNP concentration: 10, 20, 30, and 40 µg/well (well diffusion technique). Incubation: 28 °C, 3 days. Fungal growth record: Diameter inhibition zone.	24.2 ± 0.77 mm/40 µg, 28.2 ± 1.04 mm/40 µg For each species, respectively	[280]
Biological (*Penicillium expansum*, *Aspergillus terreus*)	14–25 10–18	Spherical Spherical	*A. ochraceus*, *A. parasiticus*, *A. niger*	Medium: PDA. Inoculum: From a spore suspension (1 × 10^6^ spores/mL). AgNP concentration: 3, 6, and 9 μg/well (5 mm). Incubation: 28 °C, 3 days. Fungal growth record: Diameter inhibition zone.	16.33 ± 96 mm/9 μg/well, No detected, 10.1–11.3 mm/6–9 μg/well For each species, respectively	[281]
Biological (*Viola odorata*)	18	Spherical	*F. oxysporum* f. sp. *radicis-lycopersici*	Medium PDA. Inoculum: Agar plugs (5 mm) from a fungal culture. AgNP concentration: 600 ppm. Incubation: 28 °C, 7 days. Fungal growth record: Colony diameter. Medium: Tomato plants. Inoculum: From a spore suspension (1.5 × 10^4^ spores/mL) (30 mL/plant). AgNP concentration: 60 ppm. Incubation: 24 °C ± 5 °C and photoperiod, 7 days. Fungal growth record: Percentage of plants that survive.	∼50%/600 ppm 80%/solution of 60 ppm	[309]
Commercial	—	—	*A. parasiticus*	Medium: PDB. Inoculum: From a spore suspension (1 × 10^6^ spores/mL). AgNP concentration: 60, 80, 100, 120, 140, 160, 180, and 200 ppm. Incubation: 28 °C, 96 h, 130 rpm. Fungal growth record: At a glance.	100%/180 ppm	[282]
Commercial	200– ≤0.65	Spherical	*P. verrucosum*	Medium: Malt Extract Broth. Inoculum: From a spore suspension (1 × 10^6^ spores/mL). AgNP concentration: 1–100 ppm. Incubation: 25 °C, 7 days. Fungal growth record: GNU Image Analysis Program GIMP 2.8.10.	100% spore germination/>2 ppm (AgNPs 0.65 nm) 100% fungal growth/>5 ppm (AgNPs 5 nm)	[283]
Commercial	2.0	—	*F. graminearum*	Medium: PDA. Inoculum: Agar plugs (5 mm) from a fungal culture. AgNP concentration: 1, 1.5, 2, 3, 6, 8, and 10 ppm. Incubation: 25 °C, 2–3 days. Fungal growth record: Colony diameter.	50%/1.88 ppm 90%/1.15 ppm	[186]
Commercial Argovit-1220 Argovit-1221 Argovit-C	8–80 8.5 ± 3.3 14.95 ± 10.1	Spherical, Pyramidal Spherical, Spherical	*F. oxysporum f.* sp. *cubense*	Medium: Mueller Hinton Broth + resazurin. Inoculum: From a spore suspension (1 × 10^4^ spores/mL). AgNP concentration: 0.8, 1.6, 3.1, 6.3, 12.5, 25, 50, and 100 ppm. Incubation: 28 °C, 3 days. Fungal growth record: Mycelial growth.	50%/3.1–6.3 ppm >90%/25–50 ppm Depending on commercial AgNPs	[310]
Commercial	7–25		*A. alternata*, *F. oxysporum* f. sp. *cucumerinum*, *F. oxysporum* f. sp. *lycopersici*, *F. oxysporum*, *F. solani*	Medium: PDA, MEA, and CMA (Corn Meal Agar). AgNP concentration: 10, 25, 50, and 100 ppm. Inoculum: Agar plugs (8 mm) from fungal cultures. Incubation: 28 ± 2 °C, 14 days. Fungal growth record: Colony diameter.	PDA 81.1%/100 ppm, 59.5%/100 ppm, 89.6%/100 ppm, 84.0%/100 ppm, 80.7%/100 ppm, For each strain, respectively MEA 65.35%/100 ppm, 36.5%/100 ppm, 26.5%/100 ppm, 21.2%/100 ppm, 52.9%/100 ppm, For each strain, respectively CMA 77.6%/100 ppm, 76.5%/100 ppm, 80.0%/100 ppm, 68.2%/100 ppm, 81.2%/100 ppm, For each strain, respectively	[311]
Commercial	—	—	*F. oxysporum*	Medium: PDA. Inoculum: Agar plugs (3 mm) from a fungal culture. AgNP concentration: 5, 15, 25, and 35 ppm. Incubation: 22 °C, 14 days. Fungal growth record: Colony diameter.	32.6%/35 ppm	[312]

**Table 2:** Effect of silver nanoparticles on mycotoxin production by toxigenic fungi.

Nanoparticle Properties			Anti-Mycotoxin Properties			
Synthesis Method	Size (nm)	Shape	Fungal Species (Mycotoxin Production)	Methodology	Mycotoxin/Reduction (%)/Treatment	Ref.
Chemical	14–100 (30)	Spherical	*F. graminearum* (DON, 3-AcDON, ZEA), *F. culmorum* (DON, 3-AcDON, ZEA), *F. sporotrichioides* (T-2, HT-2), *F. langsethiae* (T-2, HT-2), *F. poae* (NIV), *F. proliferatum* (FB_1_, FB_2_), *F. verticillioides* (FB_1_, FB_2_)	Medium: Maize-based medium. Inoculum: From a spore suspension (1 × 10^5^ spores/mL) previously treated with AgNPs for 2, 10, 20, and 30 h. AgNP concentration: 2, 5, 10, 15, 30, and 45 ppm. Incubation: 25–28 °C, 10 days. Mycotoxins analysis: UPLC-MS/MS.	DON/100%/2–15 ppm, 30 h, 3 AcDON/100%/2–15 ppm, 30 h, ZEA/100%/2–15 ppm. 30 h, T-2/100%/2 ppm, 30 h, HT-2/100%/2 ppm, 30 h, NIV/100%/2 ppm, 30 h, FB_1_/100%/30 ppm, 30 h, FB_2_/100%/30 ppm, 30 h, Depending on species and mycotoxin	[275]
Chemical	14–100 (30)	Spherical	*A. flavus* (AFB_1_, AFB_2_), *A. parasiticus* (AFB_1_, AFB_2_, AFG_1_, AFG_2_), *A. carbonarius* (OTA), *A. niger* (OTA), *A. ochraceus* (OTA), *A. steynii* (OTA), *A. westerdijkiae* (OTA), *P. verrucosum* (OTA)	Medium: Maize-based medium. Inoculum: From a spore suspension (1 × 10^5^ spores/mL) previously treated with AgNPs for 2, 10, 20, and 30 h. AgNP concentration: 2, 5, 10, 15, 30, and 45 ppm. Incubation: 20–37 °C, 10 days. Mycotoxins analysis: UPLC-MS/MS.	AFB_1_/100%/15–30 ppm, 30 h, AFB_2_/100%/15–30 ppm, 30 h, AFG_1_/100%/30 ppm, 30 h, AFG_2_/100%/30 ppm, 30 h, OTA/100%/5–15 ppm, 30 h, Depending on species and mycotoxin	[238]
Chemical	20	Spherical	*A. parasiticus* (AFB_1_, AFB_2_, AFG_1_, AFG_2_)	Medium: YES. Inoculum: From a spore suspension (1 × 10^5^ spores/mL). AgNP concentration: 25, 50, 100, and 200 ppm. Incubation: 28–30 °C, 14 days. Aflatoxin analysis: HPLC.	AFB_1_/6%/200 ppm, AFB_2_/57.3%/200 ppm, AFG_1_/20.4%/200 ppm, AFG_1_/95.8%/200 ppm	[276]
Chemical	17 ± 1.5	Spherical	*F. verticillioides* (FB_1_)	Medium: Nutrient Broth (according to CLSI for filamentous fungi). Inoculum: From a spore suspension (2 × 10^4^ spores/mL). AgNP concentration: 5–200 ppm. Incubation: 28 °C, 14 days. FB_1_ analysis: HPLC.	FB_1_/100%/75 ppm	[285]
Biological (*Aspergillus terreus*)	5–30	Spherical	*A. flavus* (AFB_1_) (5 strains)	Medium: SMKY broth. Inoculum: Agar plugs (6 mm) from a fungal culture. AgNP concentration: 50, 100, and 150 ppm. Incubation: 25 ± 2 °C, 20 days. Aflatoxin analysis: –.	AFB_1_/48.2–61.8%/50 ppm, AFB_1_/64.1–82.2%/100 ppm, AFB_1_/75.9–100%/150 ppm, Depending on the strain	[277]
Biological (Honeybee)	20–60	Cubic	*F. avenaceum* (DON), *F. proliferatum* (DON), *F. sambucinum* (DON), *F. verticilliodes* (DON), *F. semitectum* (DON),	Medium: SMKY broth. Inoculum: From a spore suspension (2.5 × 10^3^ spores/mL). AgNP concentration: 5, 25, 50, 75, and 100 ppm. Incubation: 25 ± 2 °C, 10 days. DON analysis: ELISA.	DON/0.03–22%/5 ppm, DON/5.61–8.46%%/25 ppm, DON/25.40–34.44%/50 ppm, DON/25.75–34.60%/75 ppm, DON/25.49–34.89%/100 ppm, For each species, respectively	[278]
Biological (*Pseudomonas poae*)	19.8–44.9	Spherical	*F. graminearum* (DON)	Medium: GYEP. Inoculum: From a spore suspension (1 × 10^6^ spores/mL). AgNP concentration: 5, 10, 15, and 20 ppm. Incubation: 28 °C, 7 days. DON analysis: ELISA.	DON/33%/5 ppm, DON/53%/10 ppm, DON/73%/15 ppm, DON/83%/20 ppm	[279]
Biological (Honey)	9.9	Spherical	*A. parasiticus* (AFB_1_, AFB_2_, AFG_1_, AFG_2_), *A. ochraceus* (OTA)	Medium: Yeast Extract Sucrose (YES). Inoculum: From a spore suspension (1 × 10^6^ spores/mL). AgNP concentration: 10, 20, and 30 ppm. Incubation: 28 °C, 14 days. Aflatoxins B_1_, B_2_, G_1_ and G_2_, and OTA. analysis: HPLC.	AFB1/58.76%/30 ppm, AFB2/66.56%/30 ppm, AFG1/77.55%/30 ppm, AFG2/62.91%/30 ppm, OTA/79.85%/30 ppm	[280]
Biological (*Penicillium expansum*, *Aspergillus terreus*)	14–25 10–18		*A. ochraceus* (OTA)	Medium: YES Broth. Inoculum: From a spore suspension (1 × 10^6^ spores/mL). AgNP concentration: 0.5, 1.1, and 2.2 ppm. Incubation: 28 °C, 14 days. OTA analysis: HPLC.	OTA/58.87–52·18%/2.2 ppm	[281]
Biological synthesis (Green and black teas)	10–20	Spherical	*A. flavus* (Aflatoxins) *A. parasiticus* (Aflatoxins)	Medium: (CZA) Czapek’s Agar. Inoculum: From a spore suspension (1 × 10^6^ spores/mL). AgNP concentration: 10, 25, 50, 100 ppm. Incubation: 25 ± 2 °C, 15 days. Aflatoxin analysis: HPLC.	Aflatoxins/100%/100 ppm	[313]
Biological synthesis (*Syzygium cumini*)	11–19	Spherical	*A. flavus* (Aflatoxins) *A. parasiticus* (Aflatoxins)	Medium: Czapek Dox Broth (CZB). Inoculum: From a spore suspension (1 × 10^6^ spores/mL). AgNP concentration: 25, 50, 100 ppm. Incubation: 25 ± 2 °C, 15 days. Aflatoxin analysis: HPLC.	Aflatoxins/100%/100 ppm	[314]
Commercial	—	—	*A. parasiticus* (AFB_1_)	Medium: PDB. Inoculum: From a spore suspension (1 × 10^6^ spores/mL). AgNP concentration: 45, 90, 100, 135, ppm. Incubation: 28 °C, 7 days, 130 rpm. AFB_1_ analysis: HPLC.	AFB_1_/50%/90 ppm AFB_1_/100%/100 ppm	[282]
Commercial	200– ≤0.65	Spherical	*P. verrucosum* (OTA, CIT)	Medium: MEA-liquid-medium. Inoculum: From a spore suspension (1 × 10^6^ spores/mL). AgNP concentration: 1–100 ppm. Incubation: 25 °C, 7 days. OTA and CIT analysis: HPLC.	DON/100%/5 ppm (AgNPs 5 nm) CIT/100%/5 ppm (AgNPs 5 nm)	[283]
Commercial	2.0	—	*F. graminearum* (DON)	Medium: Toxin biosynthesis inducing (TBI) broth medium. Inoculum: Agar plugs (5 mm) from a fungal culture. AgNP concentration: 1, 1.5, 2, 3, 6, 8, and 10 ppm. Incubation: 25 °C, 2 + 6 days. DON analysis: kit Wis008 (Wise Science, Zhenjiang, China).	DON/Increase 50%/1.15 ppm DON/Increase > 50%/1.88 ppm	[185]

The most extensive works in which the antifungal and anti-mycotoxin effect of AgNPs are studied are those of Gómez et al. [238] and Tarazona et al. [275]. As reflected in Table 1 and Table 2, the assays were executed with AgNPs exhibiting an average size of 30.9 nm (range: 14–100 nm), and obtained through chemical synthesis. The study examined the effects of doses ranging from 2 to 45 ppm of AgNPs. The treatments entailed maintaining the fungal spores in contact with the AgNPs in a liquid medium under agitation for varying durations (2 to 30 h). After the treatments, a comprehensive analysis was conducted to assess several parameters, including spore viability, the spore germination lag phase, the growth rate (GR) of colonies produced by the treated spores, and mycotoxin production. These experiments were performed on a corn-based medium. The tested species included *A. flavus*, *A. parasiticus*, *A. carbonarius*, *A. niger*, *A. ochraceus*, *A. westerdijkiae*, *A. steynii*, *Penicillium verrucosum*, *F. graminearum*, *F. culmorum*, *F. sporotrichioides*, *F. langsethiae*, *F. poae*, *F. oxysporum*, *F. proliferatum*, and *F. verticillioides.* Concurrently, the impact of these AgNPs on the production of the primary mycotoxins associated with these species was examined: aflatoxins (*A. flavus* and *A. parasiticus*), OTA (*A. carbonarius*, *A. niger*, *A. ochraceus*, *A. westerdijkiae*, *A. steynii*, *Penicillium* spp.), DON, 3-acetyldeoxynivalenol, ZEA (*F. graminearum*, *F. culmorum*), T-2 and HT-2 (*F. sporotrichioides* and *F. langsethiae*), NIV (*F. poae*), and FB_1_ and FB_2_ (*F. verticillioides* and *F. proliferatum*). The results demonstrated the significant antifungal properties of AgNPs against these species. Treatments involving extended AgNPs-spore contact times (30 h) resulted in the complete eradication of all exposed spores. In many cases, intermediate exposure times (4–20 h) yielded similar outcomes. Spores that survived the treatments exhibited a significant delay in the onset of germination and colony formation. Effective doses (EDs) to inhibit the number of viable spores to 50%, 90%, and 100% compared to untreated controls (ED_50_, ED_90_, and ED_100_) were in the range 2–45 ppm. The antifungal properties of AgNPs were influenced by various factors, including dosage, duration of exposure, the specific species of fungus, and the interaction between these factors. All these factors significantly influence spore viability, lag period, GR, and mycotoxin production. Work of this type is necessary because these toxigenic fungi and mycotoxins often coexist on the same substrate, and it is important to know the spectrum of antifungal and anti-mycotoxin action of the tested NPs to determine more precisely their possible effectiveness in practice. Other authors have described higher effective doses of NPs obtained by chemical synthesis. Thus, Sedaghati et al. [276] observed a decrease in mycelial mass of *A. parasiticus* of 92% in cultures treated with AgNP doses of 250 ppm, and Dananjaya et al. [315] observed a decrease of 81.52% in colonial growth of *F. oxysporum* using an AgNP dose of 1000 ppm.

The results described for AgNPs obtained by biological synthesis differ widely. Bocate et al. [288], using broth microdilution assays, incubated fungi at 30 °C for 48 h, found minimal inhibitory concentrations (MICs) for AgNPs of 8, 4, 8, 8, 4, and 8 ppm for *A. flavus*, *A. melleus*, *A. nomius*, *A. ochraceus*, and *A. parasiticus*, respectively, and minimal fungicidal concentrations (MFCs) of 64, 16, 32, 32, 16, and 32 ppm, respectively. However, Yassin et al. [316] studied the effect of AgNPs on *A. flavus* var. *columnaris* in potato dextrose agar (PDA) medium supplemented with AgNPs (size 3–13 nm); the cultures were incubated at 28 °C for 10 days, and colony diameters were measured daily. In these cultures, the ED_50_ and ED_95_ were 224.5 ppm and 4001.8 ppm, respectively. The effect of AgNPs on mycotoxin production was not studied in these reports.

The results reported in the literature for commercial AgNPs also vary (Table 1 and Table 2). Five ppm of AgNPs with a diameter of 5 nm and 2 ppm of AgNPs with a diameter of 0.65 nm inhibited the growth and spore germination of *P. verrucosum*, respectively [283]. It was also found that 5 ppm of AgNPs with a diameter of 5 nm inhibited OTA and CIT production. However, it has been documented that concentrations of 100 ppm of 7–25 nm AgNPs were necessary to inhibit the growth of *A. alternata*, *F. oxysporum* f. sp. *cucumerinum*, *F. oxysporum* f. sp. *lycopersici*, *F. oxysporum*, and *F. solani* in percentages ranging from 59.5% to 81.1% on PDA medium, 26.5% to 65.35% on malt extract agar (MEA) medium, and 76.5% to 81.2% on corn meal agar (CMA) medium [310]. The possible mycotoxins remaining in the medium were not determined.

The interpretation of the results reported in the literature regarding AgNP assays for the control of toxigenic fungi growth and mycotoxin production is not easy. Several factors influence this issue, primarily the discrepancies found in some reports concerning the application of microbiological and analytical chemistry methods in antifungal assays and in the determination of mycotoxins in in vitro cultures, along with their significant heterogeneity. Furthermore, the utilization of fungal strains classified as mycotoxin producers remains sporadic. Moreover, conducting a comparative analysis of the studies on the effectiveness of AgNPs in controlling the growth of toxigenic fungi and mycotoxin production is challenging. This is because each work employs different AgNPs (size, shape, type of synthesis, etc.), methodologies, culture conditions, and fungal species and strains. Standardized effective doses (EDs), such as the ED_50_, ED_90_, or ED_100_ of the AgNPs, fungal GR, etc. [238,275], which allow conclusions to be drawn, are rarely calculated. There are even different interpretations of the MIC concept [275,288,291,296].

Despite these limitations, the general conclusion that can be drawn from the trials performed so far is that AgNPs are very effective in controlling the growth of numerous species described as mycotoxin producers and do not induce resistance. This review also indicates that the antifungal and antimycotoxin effects of the AgNPs are similar; the absence or reduction of fungal growth is associated with the absence or reduction of mycotoxins in the medium. However, it has been detected that sublethal AgNP doses can stimulate DON production [186]. While this result is timely, further research is needed to understand the effects of AgNPs on the expression of genes involved in mycotoxin production under various environmental conditions. In addition, it will be essential to investigate the impact of AgNPs on fungal growth and mycotoxin production, as studies that address both of these factors simultaneously are limited.

### 5.2. Copper Nanoparticles

Copper (Cu) is one of the most essential micronutrient metals/minerals required by living organisms (humans, animals, and plants) [317]. In adults, the average human body contains between 1.4 and 2.1 mg of Cu per kg of body weight (kg/bw). According to the World Health Organization (WHO), the recommended daily upper limit of Cu for adults is 2–3 mg. However, this small amount is essential to the overall well-being of humans. This element is crucial for growth and development, as well as the maturation of the nervous, bone, and other systems. It is also an essential component of enzymes that facilitate glucose, amino acids, and cholesterol metabolism, along with various catalytic reactions. As with other trace elements, Cu must be kept in equilibrium in the body. It is not synthesized in the body and thus must be obtained through dietary sources. The primary dietary sources of copper for humans and animals include three main categories: seafood (e.g., oysters and other shellfish), organ meats (e.g., kidneys and liver), and dark leafy greens, whole grains, legumes (e.g., beans and lentils), nuts, potatoes, and dried fruits (e.g., prunes, cocoa, and black pepper). The use of Cu as a nutritional dietary supplement in animal feeds has been categorized as “generally recognized as safe” (GRAS) by both the US Food and Drug Administration (US FDA) and the European Food Safety Authority (EFSA) Panel on Additives and Products or Substances used in Animal Feed (FEEDAP Panel) [318]. For optimal plant growth and development, the concentration of Cu ranges between 0.05 and 0.5 ppm, with most tissues (e.g., vascular, dermal, and ground tissue cells) between 3 and 10 ppm [139]. Cu is involved in a variety of physiological processes, including the formation of chlorophyll and photosynthesis, as well as assisting in plant respiration and the metabolism of carbohydrates and proteins. Some of the enzymatic reactions use Cu as a cofactor, including the activation of metalloproteins and those involved in lignin synthesis [193]. The primary source of Cu for plants is fertilizers and several fungicides, which contain Cu as their active ingredient. The use of Cu is advantageous for several reasons. First, it is significantly more affordable than its counterpart, silver. Second, it is less toxic and highly available. Third, it has been reported to be effective against a variety of pathogenic and toxigenic fungi, including *A. carbonarius*, *A. fumigatus*, *A. niger*, *Alternaria solani*, and *F. expansum* [319,320].

Generally employed fungicides are not approved for use in organic agriculture. The use of Cu antifungal agents has resulted in a new option in this field. Cu is recognized as a potent antimicrobial metal with comprehensive control. Some copper is classified as a potent antimicrobial element with comprehensive control. Furthermore, some copper-based fungicides, which are divided into both inorganic and organic fungicides, are approved for organic farming practices [139,321]. Cu-based fungicides are categorized as Cu hydroxide fungicides, Cu oxychloride fungicides, and Cu oxide fungicides, and their application can be made through suspension concentrate, wettable powder, and water granules [193].

However, in both humans and animals, abnormal Cu metabolism or content has been associated with a multitude of diseases, including those associated with immune function deterioration, diabetes, coronary heart disease, and osteoporosis [322]. In agriculture, the consistent use and effectiveness of Cu fungicides pose several challenges. For instance, the accumulation of Cu in roots results in plant toxicity, which restricts root growth by burning the root tips and thereby causing excess lateral root growth. Additionally, the build-up of copper in sediments can lead to long-term soil contamination [320,323,324].

To address these challenges, there is a constant search for alternatives to optimize Cu fungicides with limited to no toxicity effects. Due to their cost-effectiveness, unique nanoscale properties, well-established antimicrobial activity, and broad-spectrum applicability, CuNPs are showing great potential in agricultural applications for pest management and disease control. Diverse commercial Cu nanoformulations are utilized in agriculture [193]. Copper-based NPs, such as CuNPs and CuONPs, are used frequently in nanoformulations and agrochemicals because of their ability to efficiently deliver and control the pesticide and fertilizer release. Other agricultural applications include the protection of plants, promotion of plant growth, the use of nanosensors, and the use of antifungal and antibacterial activities [194]. Synthesizing CuNPs presents a significant challenge due to the rapid oxidation of copper, which results in the formation of Cu_2_O, CuO, and Cu(OH)_2_. Consequently, there are various studies on the use of CuNPs and CuONPs against toxigenic fungi. The primary synthesis methods used to produce these metal NPs, as well as the assays employed to assess their efficacy against the specified mycotoxin-producing species and mycotoxin production, are outlined in Table 3, Table 4 and Table 5.

CuNPs and CuONPs have been studied less frequently than AgNPs, likely due to observations indicating their reduced effectiveness against toxigenic fungal species [285]. As with AgNPs, the literature on the antifungal and anti-mycotoxin effects of CuNPs or CuONPs contains a great deal of variation. This heterogeneity is seen in the methods for their synthesis, physicochemical characteristics, and the tests on their effectiveness against fungi and mycotoxin production (Table 3, Table 4 and Table 5). In reports, there is often a lack of information necessary for accurate reproduction of antifungal assays and comparative analysis.

As in the case of AgNPs, the CuNPs and CuONPs morphology is generally spherical, and their size ranges between 2 and 500 nm (Table 3, Table 4 and Table 5). The most commonly used antifungal activity assays are well diffusion methods, radial colony growth, and MIC determination. The size of growth inhibition halos, reduction percentage, total inhibition of the fungal colony growth, and MIC values also vary widely among reports. However, in all cases, CuNPs or CuONPs are effective as antifungal and anti-mycotoxin agents to a greater or lesser extent.

Among the studies shown in Table 3 and Table 5, the one by Pérez-de-León et al. [285] is noteworthy because it is the only one in which the effectiveness of CuNPs against fungal growth (in this case, *F. verticillioides*) and against mycotoxin production (in this case, FB_1_) is studied simultaneously. In this study, a comparative analysis of the effectiveness of CuNPs (2.5 ± 0.3 nm) and AgNPs (17 ± 1.5 nm) obtained in both cases by the same chemical reduction method and under the same conditions is also carried out. Results revealed that both these NPs exhibited significant antifungal activity against *F. verticillioides*, at 125 and 75 ppm for CuNPs and AgNPs, respectively. Regarding the control of FB_1_ production, the CuNPs completely inhibited FB_1_ production at levels ≥ 100 ppm (Table 5), while AgNPs suppressed FB_1_ production at levels ≥ 20 ppm (Table 2). The authors suggest that the activity of treatment with CuNPs and AgNPs occurred due to changes caused in the structure of the hyphae, such as the interference in mycelial growth, loss of contour and uniformity of the hyphae, and rupture of the hyphae, resulting in a significant reduction in FB_1_ biosynthesis. It is worth noting that although AgNPs perform better than CuNPs in controlling *F. verticillioides* and producing FB_1_, the major drawback of the CuNPs tested in [285] for their possible application in food preservation is that their size is excessively small, so that their toxicity to the consumer could be high. In other report [325], larger CuNPs (200–500 nm) also obtained by chemical synthesis are used against *F. oxysporum* f. sp. *lycopersici* with good results. However, as expected, the concentrations required for a significant reduction of fungal growth (80%) were 1000 ppm, while in [286], the MIC against *F. verticillioides* was 125 ppm.

As stated in another report [313], CuNPs between 26 and 40 nm, produced biologically from green and black tea extracts, were found to decrease aflatoxin (AF) production by 83.1 ± 2.9% at a concentration of 100 ppm for both *A. flavus* and *A. parasiticus*. Similarly, AgNPs, synthesized using the aforementioned method, exhibited a 100% reduction in AF production. In a similar study, extracts of Syzygium cumini leaves were used for synthesis [314]. The resulting CuNPs (28–35 nm) at a concentration of 100 ppm reduced AF production by 75.7 ± 3.2% (Table 5). Moreover, AgNPs obtained by the same method and at the same concentration inhibited AF biosynthesis (Table 2). Despite the higher antifungal and anti-mycotoxin effectiveness of AgNPs compared to CuNPs reported in these studies, CuNPs may be a more suitable choice for agricultural and food technology applications because Cu is an essential micronutrient for living organisms and promotes plant growth.

In summary, the responsible and sustainable use of Cu nanoformulations provides a noteworthy strategy to reduce the amount of Cu introduced into agroecosystems. This strategy effectively mitigates ecotoxicological risks while concurrently delivering substantial Cu for antifungal and micronutrient cofactor activities. These are vital for facilitating essential plant pathways. However, the use of Cu nanofungicides has proven to be more beneficial than conventional fungicides. Investing in understanding the ecotoxicology of the Cu nanoformulation lifecycle is crucial. The structural design of Cu nanofungicides has the capacity to influence the physicochemical, fungicidal, and fungistatic properties attributed to bioavailability and cupric (Cu^2+^) ion release as a function of antifungal activity and overall toxicity profile. To avert toxicity, it is important to consider green nanotechnologies and dosimetric calculations. This combination of green, sustainable nanotechnology enhances fungicidal activity, thereby eliminating the risk of mycotoxins. It will enhance crop and food productivity and management while avoiding ecotoxicological or phytotoxic effects in agroecosystems (crops, soil), animals, or humans.

**Table 3:** Antifungal effect of copper nanoparticles against toxigenic fungi.

Nanoparticle Properties			Antifungal Properties			
Synthesis Method	Size (nm)	Shape	Fungal Species	Methodology	Growth Reduction (%)/Treatment	Ref.
Chemical	3–10	Spherical	*A. alternata*, *F. oxysporum*	Medium: PDA. Inoculum: —. CuNP concentration: 20 μg/disc (disc diffusion method. Incubation: 28 °C, 2–3 days. Fungal growth record: Diameter inhibition zone.	18 ± 1.1 mm/20 μg/disc, 24 ± 0.5 mm/20 μg/disc, For each species, respectively	[326]
Chemical	100–500	Flower	*A. niger*, *F. moniliforme*, *F. culmorum*, *F. oxysporum*, *F. tricinctum*	Medium: PDA. Inoculum: —. CuNP concentration: 100 mM (disc diffusion method). Incubation: 28 ± 2 °C, 2–3 days. Fungal growth record: Diameter inhibition zone.	∼0–30 mm/100 mM Depending on the fungal species	[327]
Chemical	14–37	Truncated, octahedrons	*F. oxysporum*	Medium: PDA. Inoculum: From a spore suspension (1 × 10^6^ spores/mL). CuNP concentration: 100, 250, and 500 ppm. Incubation: 29 °C, 6 days. Fungal growth record: Colony diameter.	90–100%/500 ppm	[328]
Chemical	50	Spherical	*A. flavus*, *P. chrysogenum*	Medium: PDA. Inoculum: From a spore suspension (1 × 10^5^ spores/mL). CuNP concentration: 1 mM. Incubation: 26–27 °C, 60 h. Fungal growth record: Diameter inhibition zone.	24–27 mm/1 mM	[295]
Chemical	20–50	Spherical	*Fusarium* sp.	Medium: PDA (+ chloramphenicol). Inoculum: —. CuNP concentration: 300, 380, and 450 ppm. Incubation: —, 9 days. Fungal growth record: Colony diameter.	67.38%/450 ppm (3 days) 93.98%/450 ppm (9 days)	[329]
Chemical	200–500	—	*F. oxysporum* f. sp. *lycopersici*	Medium: PDA. Inoculum: From a spore suspension (1 × 10^6^ spores/mL). CuNP concentration 100, 250, 500, 750, and 1000 ppm. Incubation: 29 °C, 7 days. Fungal growth record: Colony diameter. Medium: Tomato plants. Inoculum: From a spore suspension (1 × 10^6^ spores/mL). CuNP concentration: 500 ppm. Incubation: room temperature (June-August), 60 days. Fungal growth record: Ratio of leaves with symptoms/total leaves.	>80%/1000 ppm 70%/500 ppm	[325]
Chemical	14 ± 2	Spherical	*A. niger*, *A. oryzae*	Medium: PDA. Inoculum: From a spore suspension (—). CuNP concentration: 0.2, 0.4, 0.6, and 0.8 ppm (well diffusion method). Incubation: 28 ± 4 °C, 2 days. Fungal growth record: Diameter inhibition zone.	17–24 mm/0.2–0.8 ppm. 15–20 mm/0.2–0.8 ppm. For each species, respectively	[330]
Chemical	8	Spherical	*P. chrysogenum*, *A. alternata*, *F. solani*, *A. flavus*	Medium: PDA. Inoculum: From a spore suspension (1 × 10^4^ spores/mL). CuNP concentration: 20, 40, 60, 80, and 100 ppm. Incubation: 25 °C, 6 days. Fungal growth record: MIC.	100%/40 ppm, 100%/60 ppm, 100%/60 ppm, 100%/80 ppm, For each species, respectively	[331]
Chemical	53–174	Spherical	*F. oxysporum*	Medium: PDA. Inoculum: — (well 4 mm). CuNP concentration: 5, 10, and 20 ppm. Incubation: 30 °C, 2–5 days. Fungal growth record: Diameter inhibition zone.	49–72%/20 ppm	[332]
Chemical	3–30	Spherical	*F. culmorum*, *F. oxysporum*, *F. equiseti*	Medium: PDA. Inoculum: From a spore suspension (—). CuNP concentration: —. Incubation: 28 ± 2 °C, 3 days. Fungal growth record: Diameter inhibition zone.	19 mm/—, 20 mm/—, 25 mm/—, For each species, respectively	[333]
Chemical	200–500	—	*F. solani*, *F. oxysporum*	Medium: PDA. Inoculum: From a spore suspension (1 × 10^6^ spores/mL). CuNP concentration: 100, 250, 500, 750, and 1000 ppm. Incubation: 29 °C, 6 days. Fungal growth record: Colony diameter.	95–97%/500 ppm	[334]
Chemical	2.5 ± 0.3	Spherical	*F. verticillioides*	Medium: Nutrient Broth (according to CLSI for filamentous fungi). Inoculum: From a spore suspension (2 × 10^4^ spores/mL). CuNP concentration: 5–200 ppm. Incubation: 25 °C, 48 h. Fungal growth record: MIC.	100%/125 ppm	[285]
Chemical	—	—	*A. niger*	Medium: PDA. Inoculum: —. CuNP concentration: 0.5, 1, and 1.5%. Incubation: 30 °C, 3 days. Fungal growth record: Colony diameter.	∼35–100%/0.5–1.5%	[335]
Biological (*Streptomyces capillispiralis Ca-1*)	3.6–59	Spherical	*Alternaria* spp., *A. niger*, *Fusarium* spp.	Medium: PDA. Inoculum: Agar plugs (4 mm) from a fungal culture. CuNP Concentration: 5, 10, 15, and 20 mM. Incubation: 30 °C, 7 days. Fungal growth record: Colony diameter.	57.14%/20 mM, 63.81%/20 mM, 42.61%/20 mM, For each species, respectively	[35]
Biological (*Citrus medica* Linn)	10–60 (33)	Spherical	*F. culmorum*, *F. oxysporum*, *F. graminearum*	Medium: PDA. Inoculum: From a spore suspension (—). CuNP concentration: 2.18 × 10^8^ particles/mL Incubation: 25 ± 2 °C, 52 h. Fungal growth record: Diameter inhibition zone.	∼20–33 mm/2.18 × 10^8^ particles/mL Depending on the fungal species	[336]
Biological (*Talaromyces pinophilus*)	9	Spherical	*A. niger*, *A. terreus*, *A. fumigatus*	Medium: Malt Extract Agar (MEA). Inoculum: From a spore suspension (1 × 10^7^ spores/mL). CuNP concentration: 2000 ppm (wells 7.5 mm). Incubation: 30 °C, 72 h. Fungal growth record: Diameter inhibition zone.	21.3 ± 0.58 mm/2000 ppm, 20.2 ± 1.26 mm/2000 ppm, 22.3 ± 1.53 mm/2000 ppm, For each species, respectively	[337]
Biological (*Celastrus paniculatus* leaves)	2–10	Spherical	*F. oxysporum*	Medium: PDA. Inoculum: Agar plugs (—) from a fungal culture. CuNP concentration: 0.12, 0.18, and 0.24%, *w*/*v*. Incubation: —. Fungal growth record: Colony diameter.	76.29 ± 1.52%/0.24% 73.70 ± 1.52%/0.18% 59.25 ± 0.57%/0.12%	[338]
Commercial	25	—	*A. alternata*, *F. solani*, *F. oxysporum* f. sp. *radicis lycopersici*	Medium: PDA. Inoculum: Agar plug (5 mm) from a fungal culture. CuNP concentration: 0, 1, 10, 100, 500, 1000 ppm. Incubation: 25 °C, 4 days. Fungal growth record: Colony diameter. Medium: PDA. Inoculum: From a spore suspension (1 × 10^3^ spores/mL). CuNP concentration: 0, 1, 2.5, 5, 10, 20, 50, 100 ppm. Incubation: 25 °C, 2 days. Fungal growth record: CFU.	50%/296.56 ± 8.72 ppm 50%/261.16 ± 12.54 ppm 50%/328.12 ± 20.30 ppm For each species, respectively 50%/7.69 ± 1.00 ppm, 50%/18.84 ± 2.44 ppm, 50%/29.04 ± 4.32 ppm, For each species, respectively	[339]

**Table 4:** Antifungal effect of copper oxide nanoparticles against toxigenic fungi.

Nanoparticle Properties			Antifungal Properties			
Synthesis Method	Size (nm)	Shape	Fungal Species	Methodology	Growth Reduction (%)/Treatment	Ref.
Biological (*Morinda citrifolia*)	20–50 (29)	Spherical	*A. flavus*, *A. niger*	Medium: Sabouraud Dextrose agar (SDA). Inoculum: From a spore suspension (—). CuONP concentration: — (wells 5 mm). Incubation: 37 °C, 24 h. Fungal growth record: Diameter inhibition zone.	7.6–13.1 mm/—, 9.2–14.7 mm/—, For each species, respectively	[340]
Biological (*Penicillium chrysogenum*)	10.5–59.7	Spherical	*F. solani*, *F. oxysporum*, *A. terreus*	Medium: PDA. Inoculum: —. CuONP concentration: 10,000 ppm (discs 7 mm). Incubation: 30 °C, 5 days. Fungal growth record: Diameter inhibition zone.	31.66 ± 0.88 mm/10,000 ppm, 22.66 ± 0.66 mm/10,000 ppm, 28.66 ± 1.76 mm/10,000 ppm, For each species, respectively	[341]
Biological (*Cissus quadrangularis*)	30 ± 2	Spherical	*A. niger*, *A. flavus*	Medium: PDB. Inoculum: —. CuONP concentration: 500 and 1000 ppm. Incubation: —, 7 days. Fungal growth record: Fungal biomass.	86%/1000 ppm, 85%/1000 ppm, For each species, respectively	[342]
Biological (*Bougainvillea* flower)	5–20 (12 ± 4)	Spherical	*A. niger*	Medium: PDA. Inoculum: From a spore suspension (—). CuONP concentration: 5000 ppm. Incubation: 30 °C, 1 day. Fungal growth record: Diameter inhibition zone.	80%/5000 ppm	[343]
Biological (*Eichhornia crassipes*)	28 ± 4	Spherical	*F. culmorum*, *A. niger*, *A. flavus*, *F. oxysporum*	Medium: PDA. Inoculum: —. CuONP concentration: 25, 50, 75, and 100 ppm. Incubation: Room temperature, 2 days. Fungal growth record: Diameter inhibition zone.	21.26 ± 1 mm/100 ppm, 18.33 ± 1 mm/100 ppm, 16–18 mm/100 ppm, 15–17 mm/100 ppm, For each species, respectively	[344]
Biological (*Aloe vera*)	11 ± 0.5	Spherical	*P. digitatum*, *P. italicum*	Medium: PDA. Inoculum: From a spore suspension (1 × 10^6^ spores/mL) (treated with CuONPs for 24 h). CuONP concentration: 100–1000 ppm. Incubation: 22 ± 1 °C, 4 days. Fungal growth record: CFU. Medium: Lemons. Inoculum: Steel rod previously immersed in conidial suspensions (treated with CuONPs for 24 h). CuONP concentration: 100–1000 ppm. Incubation: 20 °C, 95% RH, 5–14 days. Fungal growth record: Lemon disease incidence.	100%/1000 ppm 100%/1000 ppm	[287]
Biological (Lemon peels extract)	16.8	Rounded, elongated spherical	*A. citri*	Medium: PDA. Inoculum: From a spore suspension (—). CuONP concentration: 10–100 ppm. Incubation: 28 °C, 5 days. Fungal growth record: Diameter inhibition zone.	18.5–50 mm/10–100 ppm	[345]
Biological (*Penicillium chrysogenum*)	9.7	—	*F. oxysporum*, *A. solani*, *A. niger*, *P. citrinum*	Medium: Sabouraud Dextrose Agar. Inoculum: —. CuONP concentration: 250 ppm. Incubation: 28 °C, 5 days. Fungal growth record: Diameter inhibition zone.	37.0 mm/250 ppm, 28.0 mm/250 ppm, 26.5 mm/250 ppm, 20.7 mm/250 ppm, For each species, respectively	[346]
Commercial	46	Spherical	*F. oxysporum*, *A. solani*	Medium: PDA. Inoculum: Agar plugs (5 mm) from a fungal culture. CuONP concentration: 100, 250, 500, 700, and 1000 ppm. Incubation: 25 ± 2 °C, 7–11 days. Fungal growth record: Colony diameter.	31.48–95.57%/100–1000 ppm, 10.69–95.4%/100–1000 ppm, For each species, respectively	[347]

**Table 5:** Antifungal effect of copper nanoparticles on mycotoxin production by toxigenic fungi.

Nanoparticle Properties			Antifungal Properties			
Synthesis Method	Size (nm)	Shape	Fungal Species	Methodology	Mycotoxin/Reduction (%)/Treatment	Ref.
Chemical	2.5 ± 0.3	Spherical	*F. verticillioides* (FB_1_)	Medium: Nutrient broth (according to CLSI for filamentous fungi). Inoculum: From a spore suspension (2 × 10^4^ spores/mL). CuNP Concentration: 5–200 ppm. Incubation: 28 °C, 14 days. Fumonisin B_1_ analysis: HPLC.	FB1/100%/100 ppm	[285]
Biological (Green and black tea leaves)	26–40	Spherical	*A. flavus* (Aflatoxins) *A. parasiticus* (Aflatoxins)	Medium: Czapek Dox agar Inoculum: From a spore suspension (1 × 10^6^ spores/mL) CuNP Concentration: 10, 25, 50, and 100 ppm Incubation: 25 ± 2 °C, 15 days Aflatoxin analysis: HPLC	AFs/11.3 ± 1.2–83.1 ± 2.9/10–100 ppm	[313]
Biological (*Syzygium cumini* leaves)	28−35	Spherical	*A. flavus* (Aflatoxins) *A. parasiticus* (Aflatoxins)	Medium: Czapek Dox Liquid Inoculum: From a spore suspension (1 × 10^6^ spores/mL) CuNP Concentration: 25, 50, and 100 ppm Incubation: 25 ± 2 °C, 15 days Aflatoxin analysis: HPLC	AFs/75.7± 3.2/100 ppm 80.0 ± 2.1/100 ppm	[314]

### 5.3. Zinc Nanoparticles

Zinc (Zn) is an essential component of life; after iron, it is the second most abundant transition metal ion (Zn^2+^) in living organisms. Approximately 5–6% and 9–10% of proteins in prokaryotes and eukaryotes, respectively, are dependent on Zn for their biological functions. It is essential for cell growth and division, immune function, enzyme reactions, as well as DNA and protein synthesis. Zn has been shown to effectively reduce inflammation, boost immune health, reduce the risk of age-related diseases, and speed wound healing. Notably, more than 50% of Zn-binding proteins are enzymes. In most of these cases, the metal plays a catalytic role. Approximately 20% of them utilize Zn as a structural component, while a smaller percentage function as regulators or substrates for enzyme activity. The requirement for zinc (Zn) in such a large number of proteins illustrates its critical role in many biological processes. The essential nature of Zn for cell viability, along with its toxic nature at high levels, prompted the evolution of export and import systems among prokaryotes and eukaryotes to maintain ionic homeostasis [348,349]. Additionally, Zn plays a pivotal role as an intracellular second messenger, connecting environmental changes with the regulation of metabolic activity in root nodules [350]. Zn is a naturally occurring element that plays a significant role in the metabolism of humans, animals, and plants.

Among zinc nanomaterials, zinc oxide NPs (ZnONPs) have been the focus of extensive research due to their valuable properties, including biocompatibility, eco-friendliness, low toxicity, low cost, ease of fabrication, high photosensitivity, large excitation binding energy, high thermal conductivity, and stability under harsh environmental conditions [351,352,353]. According to Mirzaei and Darroudi [354], ZnO is widely regarded as an essential nutritional supplement. Due to these properties, ZnONPs are widely used in several fields, such as agriculture and food, with a focus on drought and salinity tolerance, antimicrobial properties, and fertilizers that enhance crop yield and quality, as well as food packaging [352,355,356,357,358]. The field of medicine also holds significant potential, particularly in the realm of biomedical applications, including anti-cancer treatments. The product has been shown to possess antimicrobial, antioxidant, anti-inflammatory, wound healing, and drug delivery properties [349,352,359]. In addition, it has been identified as an effective photocatalyst agent, offering a promising method for wastewater treatment [360,361,362,363]. The ZnONP applications also extend to other fields. Given this background, ZnONPs are currently considered the most promising antibiotic nanoscale agents due to their unique properties. ZnO has been officially recognized by the U.S. Food and Drug Administration (FDA) as safe for use in food production and has been registered under the designation GRAS indicated in section 21CFR182.899 [364]. According to the current scientific consensus, the use of ZnONPs poses no significant threat to public health [364].

According to the results reported in the literature, ZnONPs have been the most studied Zn particles against fungal species described as mycotoxin producers. ZnONPs obtained by chemical synthesis, biological synthesis, and commercial formulations have undergone rigorous testing to ensure their effectiveness and safety. These reports detail in vitro assays utilizing a diverse array of NPs, encompassing a range of sizes, shapes, and concentrations (see Table 6 for details).

Pariona et al. [365] tested different ZnO particles with three characteristic shapes and synthesized by colloidal and hydrothermal techniques. They were tested against *F. oxysporum* f. sp. lycopersici and *F. solani*. The particles tested included spheroidal particles (ZnONPs) with an average diameter of 18 ± 4 nm that were arranged in agglomerates. The other particles tested were platelet particles (ZnOPls) with an average diameter of around 246 ± 40 nm and an average thickness of 48 ± 6 nm. The third type of particle tested was elongated rod particles (ZnORds) with an average diameter of 786 ± 142 nm and an average length of 9330 ± 1500 nm. The results indicated that the antifungal activity depends on both the types of ZnO particles and the specific toxigenic fungal species involved.

The order of effectiveness of the three particles was ZnOPls > ZnONPs > ZnORds (Table 6). *F. solani* was more sensitive to the treatments than *F. oxysporum*. Furthermore, the growth morphology, color, texture, and density of the fungi were observed for each treatment. For instance, the color of the mycelium of *F. oxysporum* is determined by the levels of the three ZnO particles. This phenomenon can be attributed to the active defense mechanism against the high ROS generated by the ZnO treatments [365].

This defense mechanism has also been observed in *F. oxysporum* treated with different concentrations of copper nanoparticles obtained by green synthesis [334]. Furthermore, a change in the texture of the mycelium was observed in the two fungal species treated with high concentrations of zinc oxide (ZnO) (750 and 1000 ppm). The generated ROS stimulate this effect on the texture of the mycelium by interacting with fungal biomolecules and promoting their distortion. Changes in texture have also been documented for *F. solani* and *F. oxysporum* treated with CuNPs. The effectiveness of high concentrations of ZnONPs (47.2 nm diameter) obtained by chemical synthesis against toxigenic fungi has also been studied [335]. The effects of exposure to 5000 ppm ZnONPs were examined in two strains of *Alternaria alternata* and one strain of *F. verticillioides* [368]. The study found that exposure to the chemical resulted in growth inhibition halos measuring 30.63, 36.28, and 34.77 mm, respectively.

The antifungal properties of ZnO particles obtained through biological synthesis have also been thoroughly examined. According to Jayaseelan et al. [373], ZnONPs synthesized using *Aeromonas hydrophila* demonstrated significant antifungal activity against relevant mycotoxin-producing species, such as *A. flavus* and *A. niger*. Additionally, Rajiv et al. validated the antifungal activity of ZnONPs and found that it is size-dependent. The highest inhibition was reported against *A. flavus* and *A. niger* at 25 ppm. Furthermore, the antifungal activity of ZnONPs against five fungal plant pathogens, including *A. alternata*, *A. niger*, *F. oxysporum*, and *P. expansum*, was confirmed [375]. The lowest minimum inhibitory concentration, 16 ppm, was reported against *A. niger*. Additionally, ZnONPs synthesized using *Serratia nematodiphila* exhibited activity against *Alternaria* sp. [379].

The antifungal activity of commercial ZnO particles has been tested against various species of toxigenic fungi, including *P. expansum* [384,386], *A. alternata* [384,385], *F. oxysporum* [347,386,387], and *A. solani* [347] at different concentrations using various methods. The results summarized in Table 6 show that the inhibition of fungal growth depends on the characteristics of the NP, concentration, and fungal species. The highest percentages of inhibition were obtained with the ZnOPNs used in the [347] trials, where 1000 ppm of NPs produced 91.13% and 98.69% inhibition of *F. oxysporum* and *A. solani* growth, respectively, and in the [386] trials, where 12 ppm of NPs produced 77.5% and 100% inhibition of *F. oxysporum* and *P. expansum* growth, respectively.

In addition to its capacity to regulate the proliferation of toxigenic fungi, the primary application of ZnONPs pertains to the suppression of mycotoxin synthesis. However, this aspect has received scant scholarly attention (Table 7). Savi et al. [366] investigated the inhibitory effect of ZnONPs at a 100 mM concentration on DON, AFB_1_, and CIT production in cultures of *F. graminearum*, *A. flavus*, and *P. citrinum*, respectively. The percentages of inhibition of the biosynthesis of these mycotoxins were 100% for DON and over 60% and 5% for AFs and CIT, respectively. Hassan et al. [370] found that the growth of *A. flavus* and AF production were inhibited by the addition of 8 ppm of ZnONPs. These inhibitory results were also observed for both *A. ochraceus* and OTA and *A. niger* and FB_2_ production when the concentration of ZnONPs was increased to 10 ppm. Tests with commercial ZnOPNs at a concentration of 12 ppm have demonstrated inhibition of fusaric acid production by *F. oxysporum* and patulin production by *P. expansum* by 99.5% and 92.26%, respectively. The findings indicated that the presence of ZnONPs within a food matrix may impede the proliferation of deleterious fungi and concomitantly curtail the synthesis of mycotoxins by toxigenic fungi.

### 5.4. Other Metal Oxide Nanoparticles

Research on the effect of metal oxide nanoparticles other than CuONPs and ZnONPs on toxigenic fungi and mycotoxin production is very scarce. Some studies have been conducted on the effectiveness of Fe_2_O_3_NPs [347,388] or MgONPs [369] in combating certain species of toxigenic fungi.

Iron (Fe) is a prevalent element in the human body, where it is found in various prosthetic groups, such as iron-sulfur clusters and heme, a complex of iron and protoporphyrin, or coordinated with oxygen in iron-containing enzymes. Fe is involved in different biological processes, including energy production, O_2_ transport, deoxyribonucleotide production, and replication and repair of the DNA. The regulatory framework under consideration is predicated on the premise that intestinal Fe absorption is of pivotal significance. This process is subject to the concerted influence of numerous proteins, including, but not limited to, divalent metal transporter 1, ferroportin, transferrin, and transferrin receptors. Ferroxidases, including duodenal cytochrome B, ceruloplasmin, and hephaestin, are coordinately regulated by hepcidin to ensure efficient acquisition and utilization of iron. Acute hypoferremia can affect other cells and tissues owing to the decreased synthesis of iron-containing enzymes, which are critical for cell function. Conversely, the chemical reactivity of iron contributes to its potential toxicity. Therefore, it is imperative to maintain precise balance in Fe metabolism to ensure optimal human well-being, necessitating regulation at multiple levels [389,390].

Magnesium (Mg) has been determined to be the fourth most abundant metallic element within the human body. As demonstrated in numerous studies, Mg has been shown to serve as a cofactor for over 300 enzymes, thus modulating a variety of essential physiological processes. These processes include, but are not limited to, muscle contraction, neuromuscular conduction, glycemic control, myocardial contraction, and blood pressure regulation. In addition, Mg plays a pivotal role in various physiological processes, including energy production, active transmembrane transport for other ions, the synthesis of nuclear materials, and bone development. This element has been demonstrated to play an instrumental role in the therapeutic and preventive management of a wide range of medical conditions, including but not limited to diabetes, osteoporosis, bronchial asthma, migraine, and cardiovascular illnesses [391].

Tests have been conducted using the agar diffusion method with Fe_2_O_3_NPs (10–30 nm) against *P. chrysogenum*, *A. alternata*, and *A. niger*. The results showed that 500 ppm of NPs could induce growth inhibition halos of 28.67 ± 1.53 mm, 21.33 ± 3.83 mm, and 26.33 ± 1.15 mm, respectively [388]. However, Vera-Reyes et al. [347] tested commercial Fe_2_O_3_NPs (30 nm in diameter) against *F. oxysporum* and *A. solani* at concentrations ranging from 100 to 1000 ppm. The efficacy of these NPs in reducing colony growth was negligible or minimal in comparison to ZnONPs or CuONPs, which, at 1000 ppm, resulted in a colony growth reduction of 99% and 95%, respectively. Similarly, a decline in *A. alternata* and *F. oxysporum* spore germination of 66.28 to 12.96% has been documented through the utilization of MgONPs (~50 ± 10 nm) [369]. Given the critical role of Fe and Mg in sustaining optimal organismal function, further investigation of these NPs against toxigenic fungi on a broad scale is imperative. Such research is crucial to comprehensively assess their efficacy and determine their potential to counteract toxigenic fungi and mycotoxins in food systems.

**Figure 11:**
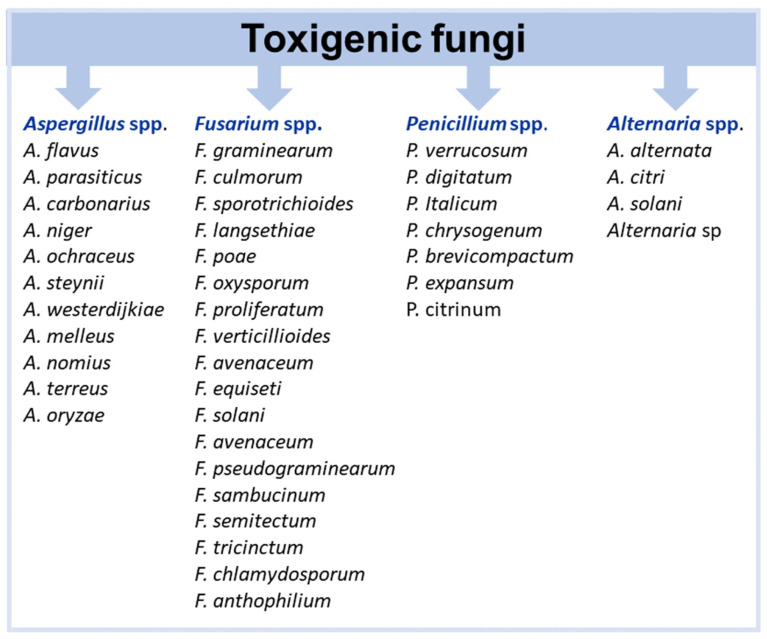
Main toxigenic fungal species assayed in cultures treated with AgNPs, CuNPs, CuONPs, and ZnONPs.

**Figure 12:**
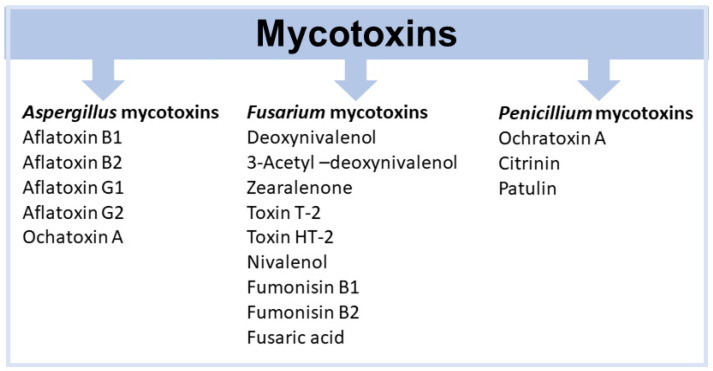
Main mycotoxins studied in cultures of toxigenic fungi treated with MNPs or MONPs.

In summary, as outlined in Figure 11, this review paper presents a comprehensive list of the predominant species of toxigenic fungi against which various NP types, including AgNPs, CuNPs, CuONPs, CuONPs, ZnONPs, Fe_2_O_3_NPs, and MgONPs, have been tested. As illustrated in Figure 12, certain NPs have been observed to inhibit the production of various mycotoxins, either completely or partially.

The extant research on the effect of MNPs and MONPs against the described mycotoxin-producing species and their consequences on the mycotoxin production by these fungi indicates that they can be excellent tools in the management of these risks in food. However, further research is required before their potential application in practice can be determined.

## 6. Engineered Metal and Metal Oxide NP as Antifungal Additives in Food

The issue of food sustainability and safety is a significant challenge faced by developing countries due to the expanding human population. The application of nanotechnology offers a straightforward, viable, and dependable solution to these challenges. Advances in nanotechnology have the potential to provide a variety of solutions to issues related to food, its storage, and shelf life, thus offering sustainable economic, commercial, and health benefits. Despite the demonstrated potential of MNPs and MONPs in a variety of applications within the food industry, significant limitations and knowledge gaps persist. In this review, we demonstrate that MNPs (AgNPs, CuNPs) and MONPs (CuONPs, ZnONPs, Fe_2_O_3_NPs, Fe_3_O_4_NPs, MgONPs) possess remarkable antifungal properties and can contribute to the prevention of contamination and the development of toxigenic fungi and mycotoxin production. These properties may offer significant benefits in food processing environments and in food itself. However, the implementation of these systems in the food industry is subject to specific regulatory frameworks and requires rigorous safety assessments before integration. Within the European Union, the utilization of nanomaterials in food is subject to a series of regulatory frameworks. According to Regulation (EU) 10/2011, the utilization of nanoform substances in food contact materials is permissible under the condition that they have been granted approval by the European Food Safety Authority (EFSA) following a comprehensive case-by-case evaluation. According to Regulation (EU) 1169/2011, all ingredients present in the form of engineered nanomaterials must be explicitly indicated in the list of ingredients, accompanied by the term “nano” in brackets. Regulation (EU) 2015/2283 [164] stipulates that any food containing or consisting of engineered nanomaterials should be classified as a novel food and undergo a rigorous evaluation before its placement on the market. Furthermore, there are concerns regarding the safety and toxicity of MNPs, particularly concerning their direct introduction into food or their potential migration into food from packaging, paints, and coatings [392,393]. Consequently, it is imperative to conduct thorough toxicological studies and adhere to current regulations before implementation in the food industry.

According to an analysis of the relevant literature, the most active area of development for engineered MNPs and MONPs, as promising nanoadditives, is in the fabrication and improvement of food packaging, paints, and coatings with antifungal properties used in the food industry [394]. This may be due to the public’s concerns regarding “nanofoods,” stemming from the uncertainties surrounding the safety of nanomaterials. Consumers are more willing to embrace nanomaterials in “out-of-food” uses than those where NPs are applied to food directly [395]. The use of MNPs or MONPs for food packaging is a rapidly growing area. Ag, Cu, CuO, and ZnO are examples of such NPs. These materials are chemically stable and characterized by an elevated aspect ratio. This review demonstrates that these NPs exhibit remarkable antifungal properties against toxigenic fungi, resulting in their growth inhibition. Consequently, they are exceptionally useful for creating innovative active materials in the biomedical and food technology sectors. It is important to note that some of the metallic constituents found in NPs are vital minerals for human physiology [348,349,389,390]. Current market demands are no longer met by traditional packaging methods. This has created a demand for more advanced and innovative approaches to food packaging. The development of novel food packaging systems has been driven by evolving market demands, including consumer preferences for “health-conscious” and high-quality food items, as well as the imperative to reduce the adverse environmental footprint of food packaging. MNPs (e.g., AgNPs, CuNPs) and MONPs (e.g., CuONPs, ZnONPs, Fe_2_O_3_NPs, Fe_3_O_4_NPs, MgONPs) are being actively explored in the food industry, especially in the manufacture of bioactive packaging [396]. Please refer to the works of Zhang et al. [397], Gopinath et al. [398], Herrera-Rivera et al. [399], Joshi et al. [400], Sun et al. [364], and Adeyemi and Fawole [401]. Smart packaging with antifungal capabilities could control the growth of toxigenic fungi, prevent the accumulation of mycotoxins, prolong the shelf life of food, and improve food safety.

## 7. Conclusions and Future Perspectives

The growing concern for food safety has prompted research into innovative strategies to mitigate food contamination by toxigenic fungi and mycotoxins. In recent decades, MNPs such as silver (Ag), copper (Cu), copper oxide (CuO), zinc oxide (ZnO), and iron oxide (Fe_2_O_3_, Fe_3_O_4_) have proven significant potential in the prevention and control of toxigenic fungi and the reduction of mycotoxins in various matrices. In this context, this review indicates that these MNPs have emerged as a promising tool due to their exceptional antifungal properties. These properties are derived from their high surface-to-volume ratio, which allows for greater interaction with fungal cells, and their ability to generate ROS that affect fungal cell integrity and disrupt essential metabolic processes.

In the near future, significant advances in the design of engineered NPs capable of selectively targeting specific toxigenic species and strains of *Aspergillus*, *Fusarium*, *Penicillium*, *Alternaria*, and *Claviceps* are expected to improve the efficiency and specificity of these NPs. The surface functionalization of NPs with specific ligands has the potential to enhance their interaction with key fungal structures while minimizing adverse effects on humans, animals, plants, and beneficial microorganisms.

Another promising area that is already experiencing growth is the incorporation of MNPs into active food packaging systems. These materials offer antifungal protection and can also act as sensors that detect the presence of fungi or mycotoxins, alerting to contamination in real time. This application is especially relevant to products such as cereals, nuts, coffee, spices, and dairy products, which are prone to spoilage.

It is imperative to prioritize the mitigation of NP toxicity and the enhancement of food safety. Notwithstanding their advantages, the utilization of MNPs gives rise to concerns regarding their toxicity to humans, animals, and the environment in general. Consequently, the design and development of biodegradable or biocompatible coated NPs that maintain their antifungal efficacy without generating significant risks to human health is crucial for future research endeavors. Toxicological evaluation at the cellular and systemic levels, as well as bioaccumulation and metabolization studies, will be essential.

Of particular relevance will be the development of hybrid technologies and synergies. The development of hybrid technologies, combining NPs with other natural antifungal compounds (e.g., essential oils or phenolic extracts), could enhance synergistic effects, allowing the use of lower concentrations and thus reducing the associated risks. Additionally, green nanotechnology, which utilizes biogenic synthesis of NPs with plant or microbial extracts, offers a sustainable alternative that could facilitate regulatory and societal acceptance.

Additionally, it will be imperative to deliberate on the implementation of NPs in agricultural and post-harvest systems. Beyond the realm of food processing, MNPs have the potential to play a crucial role in fungal and mycotoxin control during agricultural production and post-harvest storage. The development of effective formulations and their application on surfaces and coatings for grain, antifungal sprays, or seed treatments could prevent fungal contamination from the outset, reducing economic losses and enhancing food safety and security on a global scale.

## Figures and Tables

**Table 6:** Antifungal effect of zinc oxide nanoparticles against toxigenic fungi.

Nanoparticle Properties			Antifungal Properties			
Synthesis Method	Size (nm)	Shape	Fungal species	Methodology	Growth reduction (%)/Treatment	Ref.
Chemical	18 ± 4 246 ± 40 × 48 ± 6 786 ± 142 × 9330 ± 1500	Spherical Platelet Elongated rod	*F. oxysporum* f. sp. *lycopersici*, *F. solani*	Medium: Potato Dextrose Agar (PDA). Inoculum: From a spore suspension (1 × 10^6^ spores/mL). ZnONP concentration: 100, 250, 500, 750, and 1000 ppm. Incubation: 29 °C, 6 days. Fungal growth record: Colony diameter.	0–55 ± 3.1%/100–1000 ppm, 0–65 ± 1.8%/100–1000 ppm, 0–31 ± 2.1%/100–1000 ppm, Depending on the NP size and shape	[365]
Chemical	30	Spherical	*F. graminearum*, *P. citrinum*, *A. flavus*	Medium: PDA. Inoculum: Agar plugs (6 mm) from a fungal culture. ZnONP concentration: 10, 25, 50, and 100 mM. Incubation: 25 °C, 8 days. Fungal growth record: Colony diameter and mycelium weight.	∼50%/100 mM	[366]
Chemical	70 ± 15	Spherical	*P. expansum*	Medium: PDA. Inoculum: Agar plugs (14 mm) from a fungal culture. ZnONP concentration: 3, 6, and 12 mM. Incubation: 25 °C, 12 days. Fungal growth record: Colony diameter.	91%/12 mM	[367]
Chemical	47.2	Irregular	*A. alternata*, *F. verticillioides*	Medium: Malt Extract Agar (MEA). Inoculum: From a spore suspension (1 × 10^6^ spores/mL). ZnONP concentration: 2–5000 ppm. Incubation: 25 °C, 12 days. Fungal growth record: Diameter inhibition zone.	22.73–36.28 mm/2–5000 ppm, 23.77–34.77 mm/2–5000 ppm, For each species, respectively	[368]
ChemicaL	~30 ± 10		*A. alternata*, *F. oxysporum*	Medium: Suspension of spores + NPs. Inoculum: From a spore suspension (—). ZnONP concentration: —. Incubation: 24 ± 2 °C, 24 h. Fungal growth record: Spore germination.	78.00–42.61%/—	[369]
Chemical	20	Spherical	*A. flavus*, *A. ochraceus*, *A. niger*	Medium: Yeast Extract Sucrose (YES). Inoculum: From a spore suspension (5 × 10^6^ spores/mL). ZnONP concentration: (0, 2, 4, 6, 8, and 10 ppm. Incubation: 22–25 ± 2 °C, 20 days. Fungal growth record. Mycelial damage.	100%/8 ppm, 100%/10 ppm, 100%/10 ppm, For each species, respectively	[370]
Biological (*Trichoderma harzianum*)	8–23	Spherical, rod, and hexagonal	*Fusarium* sp.	Medium: PDA. Inoculum: Disks 5 mm + inoculum (—). ZnONP concentration: 20, 40, and 100 ppm. Incubation: 35 °C, 5–7 days. Fungal growth record: Colony diameter.	100%/≥20 ppm	[371]
Biological (*Cinnamomum camphora* L. leaf)	13.92–21.13	Spherical	*A. alternata*	Medium: PDA and Potato Dextrose Broth (PDB). Inoculum: Agar plugs (6 mm) from a fungal culture. ZnONP concentration: 10, 20, 40, 80, and 160 ppm. Incubation: 25 °C, 8 days. Fungal growth record: Colony diameter.	21.59–68.50%/20–160 ppm	[372]
Biological (*Aeromonas hydrophila*)	57.72	Spherical, oval	*A. flavus*, *A. niger*	Medium: Mueller Hinton Agar (MHA). Inoculum: From a spore suspension (—) (well 8 mm). ZnONP concentration: 5, 10, 15, 20, and 25 ppm. Incubation: 37 °C, 24 h. Fungal growth record: Diameter inhibition zone and MIC (according to CLSI).	19 ± 1.0 mm/25 ppm, <19 ± 1.0 mm/25 ppm, For each species, respectively 100% (MIC)/2.9 ± 0.01 ppm, 100% (MIC)/2.0 ± 0.04 ppm, For each species, respectively	[373]
Biological (*Parthenium hysterophorus* L. leaf)	27 ± 5 84 ± 2	Spherical, Hexagonal	*A. niger*, *A. flavus*, *F. oxysporum*, *F. culmorum*	Medium: PDA. Inoculum: From a spore suspension (—). ZnONP concentration: 25 ppm (wells 5 mm). Incubation: Room temperature, 48 h. Fungal growth record: Diameter inhibition zone.	24–14 mm/50 ppm (size 27 ± 5 nm), 21–12 mm/50 ppm (size 84 ± 2 nm), Depending on the species	[374]
Biological (*Nyctanthes arbor-tristis* flower)	12–32	Spherical	*A. alternata*, *A. niger*, *F. oxysporum*, *P. expansum*	Medium: Liquid medium (—). Inoculum: From a spore suspension (1 × 10^5^ spores/mL). ZnONP concentration: 16–256 ppm. Incubation: 28 °C, 3 days. Fungal growth record: MIC.	100%/64 ppm, 100%/16 ppm, 100%/64 ppm, 100%/128 ppm, For each species, respectively	[375]
Biological (*Penicillium chrysogenum*)	9.0–35.0	Hexagonal	*F. solani*, *F. oxysporum*, *A. terreus*	Medium: PDA. Inoculum: From a spore suspension (—). ZnONP concentration: 10,000 ppm (discs 7 mm). Incubation: 30 °C, 5 days. Fungal growth record: Diameter inhibition zone.	12.33 ± 0.88/10,000 ppm, 11.83 ± 1.36/10,000 ppm, 14 ± 0.5 mm/10,000 ppm, For each species, respectively	[341]
Biological (*Daedalea Mushroom*)	18.53	Irregular	*A. niger*	Medium: PDA. Inoculum: Agar plugs (1.4 cm) from a fungal culture. ZnONP concentration: 100, 250, 750 ppm. Incubation: 26 °C, 6 days. Fungal growth record: Colony diameter.	22%/100 ppm, 88%/250 ppm	[376]
Biological (*Chlorella vulgaris*)	35	Hexagonal	*A. flavus*	Medium: MHA. Inoculum: From a spore suspension (2–5 × 10^5^ spores/mL). ZnONP concentration: 10,000 ppm (disks). Incubation: 35 °C, 2 days. Fungal growth record: Diameter inhibition.	13–14 mm/10,000 ppm	[377]
Biological (*Tinospora cordifolia*)	32	Hexagonal	*F. oxysporum*	Medium: PDA. Inoculum: From spore suspension (—). ZnONP concentration: 100 ppm (wells 6 mm). Incubation: 25 °C, 5–7 days. Fungal growth record: Diameter inhibition zone.	47–51 mm/100 ppm	[378]
Biological (*Serratia nematodiphila*)	15–30 (23)	Hexagonal	*Alternaria* sp.	Medium: PDA. Inoculum: Agar plugs (4 mm) from a fungal culture. ZnONP concentration: 50, 100, 150, 200, and 250 ppm. Incubation: (—), 5 days. Fungal growth record: Colony diameter (cd) and spore viability (sv).	21.67–85.93% (cd)/50–250 ppm, 18.18–92.22% (sv)/50–250 ppm	[379]
Biological (*Sargassum vulgare*)	50–150	Spherical	*A. niger*, *A. flavus*	Medium: Sabouraud Dextrose Agar (SDA). Inoculum: From a spore suspension (1 × 10^6^ spores/mL). ZnONP concentration: 1, 5, 10, 30, and 50 ppm (wells). Incubation: 22 °C, 3 days. Fungal growth record: Diameter inhibition zone, MIC, and MFC.	11–32 mm/1–50 ppm, 9–27 mm/1–50 ppm, For each species, respectively MIC and MFC/10 and 20 ppm, MIC and MFC/40 and 50 ppm, For each species, respectively	[380]
Biological (Lemon peels extract)	16.8	Rounded, Elongated, Spherical	*A. citri*	Medium: PDA. Inoculum: From a spore suspension (—). ZnONP concentration: 10–100 ppm. Incubation: 28 °C, 5 days. Fungal growth record: Diameter inhibition zone.	21.5–51.5 mm/10–100 ppm	[345]
Biological (*Melia azedarach* leaf)	30–40	Hexagonal	*F. oxysporum*	Medium: *Czapek-Dox* Broth (CZB) and *Czapek-Dox* agar (CZA). Inoculum: From a spore suspension (1 × 10^6^ spores/mL). ZnONP concentration: 250 ppm. Incubation: 25 ± 2 °C, 3 days. Fungal growth record: MIC and MFC.	MIC/93.33 ppm. MFC/208.3 ppm	[381]
Biological (*Pithecellobium* dulce peel)	11.5 ± 2	Spherical	*A. flavus*, *A. niger*	Medium: PDB. Inoculum: From a spore suspension (—). ZnONP concentration: 500 and 1000 ppm. Incubation: 37 °C, 3 days. Fungal growth record: Fungal biomass.	37.81–63.57%/500–1000 ppm. 40.21–43.04%/500–1000 ppm. For each species, respectively	[382]
Biological (*Saussurea lappa* plant root)	26 ± 1	Hexagonal	*A. niger*, *A. flavus*, *F. oxysporum*	Medium: —. Inoculum: —. ZnONP concentration: 50, 100, 170 ppm. Incubation: —. Fungal growth record: Diameter inhibition zone.	2.0–2.7 mm/50–170 ppm 1.7–2.7 mm/50–170 ppm 1.7–2.1 mm/50–170 ppm For each species, respectively	[383]
Commercial	<50	Spherical	*P. expansum*, *A. alternata*	Medium: PDA. Inoculum: From a spore suspension (1 × 10^5^ spores/mL). ZnONP concentration: 3, 6, 12, and 15 mM. Incubation: 25 °C, 30–112 h. Fungal growth record: Colony diameter.	50%/5.08 mM. 50%/5.49 mM. For each species, respectively	[384]
Commercial	20	Spherical	*F. oxysporum*, *A. solani*	Medium: PDA. Inoculum: Agar plugs (5 mm) from a fungal culture. ZnONP concentration: 100, 250, 500, 700, and 1000 ppm. Incubation: 25 ± 2 °C, 7–11 days. Fungal growth record: Colony diameter.	0.0–91.13%/100–1000 ppm, 29.67–98.69%/100–1000 ppm, For each species, respectively	[347]
Commercial	<100	—	*A. alternata* (10 isolates)	Medium: PDA. Inoculum: Agar plug (5 mm) from a fungal culture. ZnONP concentration: 10, 25, 50, 100, 250, 500, and 1000 ppm. Incubation: 25 °C, 70% RH, 4 days. Fungal growth record: Colony diameter. Medium: Tomato fruit Inoculum: Agar plugs (5 mm) from a fungal culture. ZnONP concentration: 1000 ppm. Incubation: 25 °C, 70% RH, 4 days. Fungal growth record: Lesion diameter.	50%/250–388 ppm (mean 303 ppm) 14.24–32.50%/1000 ppm	[385]
Commercial	70 ± 15	—	*F. oxysporum*, *P. expansum*	Medium: PDA. Inoculum: Agar plugs (1 cm) from a fungal culture. ZnONP concentration: 0, 2, 4, 6, 8, and 12 ppm. Incubation: 25 °C, 12 days. Fungal growth record: Colony diameter.	19.3–77.5%/2–12 ppm, 25.3–100%/2–12 ppm, For each species, respectively	[386]
Commercial	170–430	—	*F. oxysporum*	Medium: PDA. Inoculum: Agar plugs (0.5 mm) from a fungal culture. ZnONP concentration: 10^−3^ M and 5 × 10^−3^ M. Incubation: 25 °C (—). Fungal growth record: Colony diameter.	51.7%/5 × 10^−3^ M photoactivated	[387]

**Table 7:** Effect of zinc oxide nanoparticles on mycotoxin production by toxigenic fungi.

Nanoparticle Properties			Anti-Mycotoxin Properties			
Synthesis Method	Size (nm)	Shape	Fungal Species	Methodology	Mycotoxin/Reduction (%)/Treatment	Ref
Chemical	30	Spherical	*F. graminearum* (DON), *A. flavus* (AFB_1_), *P. citrinum* (CIT)	Medium: PDA. Inoculum: Agar plugs (6 mm) from a fungal culture. Concentration of ZnONPs: 100 mM. Incubation: 25–30 °C, moisture 80–90%, 20 days. Deoxynivalenol (DON), aflatoxin B_1_ (AFB_1_), and citrinin (CIT) analysis: TLC.	DON/100%/100 mM AFB_1_/∼60%/100 mM CIT/∼5%/100 mM	[366]
Chemical	20	Spherical	*A. flavus* (Aflatoxins AFs), *A. ochraceus* (OTA), *A. niger* (FB_2_)	Medium: Yeast Extract Sucrose (YES). Inoculum: From a spore suspension (5 × 10^6^ spores/mL). Concentration of ZnONPs: 0, 2, 4, 6, 8, and 10 ppm. Incubation: 22–25 ± 2 °C, 20 days. Aflatoxins (AFs), OTA and FB_2_ analysis: HPLC.	AFs/100%/8 ppm, OTA/100%/10 ppm, FB_2_/100%/10 ppm	[370]
Commercial	70 ± 15	—	*F. oxysporum*, *P. expansum*	Medium: PDA. Inoculum: Agar plugs (1 cm) from a fungal culture. ZnONP concentration: 0, 2, 4, 6, 8, and 12 ppm. Incubation: 25 °C, 12 days. Fusaric acid (FA) and patulin (PAT) analysis: HPLC.	FA/10.26–99.5%/2–12 ppm, PAT/13.3–92.26%/2–12 ppm	[386]

## Data Availability

No new data were created in the present review.

## References

[1] Hawksworth D.L., Lücking R. (2017). Fungal Diversity Revisited: 2.2 to 3.8 Million Species. Microbiol. Spectr..

[2] Blackwell M. (2011). The fungi: 1, 2, 3 ... 5.1 million species?. Am. J. Bot..

[3] Bongomin F., Gago S., Oladele R.O., Denning D.W. (2017). Global and multi-national prevalence of fungal diseases—Estimate precision. J. Fungi.

[4] Thambugala K.M., Daranagama D.A., Tennakoon D.S., Jayatunga D.P.W., Hongsanan S., Xie N. (2024). Humans vs. Fungi: An Overview of Fungal Pathogens against Humans. Pathogens.

[5] Bastos R.W., Rossato L., Goldman G.H., Santos D.A. (2021). Fungicide effects on human fungal pathogens: Cross-resistance to medical drugs and beyond. PLoS Pathog..

[6] Denning D.W. (2024). Global Incidence and Mortality of Severe Fungal Disease. Lancet Infect. Dis..

[7] Rokas A. (2022). Evolution of the human pathogenic lifestyle in fungi. Nat. Microbiol..

[8] Brown G.D., Denning D.W., Gow N.A.R., Levitz S.M., Netea M.G., White T.C. (2012). Hidden killers: Human fungal infections. Sci. Transl. Med..

[9] Fisher M.C., Henk D.A., Briggs C.J., Brownstein J.S., Madoff L.C., McCraw S.L. (2012). Emerging fungal threats to animal, plant and ecosystem health. Nature.

[10] Pangga I.B., Salvacion A.R., Cumagun C.J.R., Botana L.M., Sainz M.J. (2015). Climate Change and Plant Diseases Caused by Mycotoxigenic Fungi: Implications for Food Security. Climate Change and Mycotoxins.

[11] Pitt J.I., Miller D. (2017). A Concise History of Mycotoxin Research. J. Agric. Food Chem..

[12] Stoev S.D. (2024). Food security, underestimated hazard of joint mycotoxin exposure and management of the risk of mycotoxin contamination. Food Control.

[13] Bezerra da Rocha M.E., Oliveira Freire F.C., Feitosa Maia F.E., Florindo Guedes M.I., Rondina D. (2014). Mycotoxins and their effects on human and animal health. Food Control.

[14] Madalena M., Sobral C., Faria M.A., Cunha S.C., Ferreira I. (2018). Toxicological interactions between mycotoxins from ubiquitous fungi: Impact on hepatic and intestinal human epithelial cells. Chemosphere.

[15] Dellafiora L., Dall’Asta C., Galaverna G. (2018). Toxicodynamics of Mycotoxins in the Framework of Food Risk Assessment: An In Silico Perspective. Toxins.

[16] Freire L., Sant’Ana A.S. (2018). Modified mycotoxins: An updated review on their formation, detection, occurrence, and toxic effects. Food Chem. Toxicol..

[17] Berthiller F., Maragos C.M., Dall’Asta C., Dall’Asta C., Berthiller F. (2016). Introduction to masked mycotoxins. Masked Mycotoxins in Food: Formation, Occurrence and Toxicological Relevance.

[18] García-Esparza M.Á., Mateo E.M., Robles J.A., Capoferri M., Jiménez M., Soria J.M. (2025). Unveiling the Neurotoxic Effects of Ochratoxin A and Its Impact on Neuroinflammation. Toxins.

[19] Moretti A.T., Logrieco A.F., Susca A., Moretti A., Susca A. (2017). Mycotoxin: An Underhand Food Problem. Mycotoxigenic Fungi: Methods and Protocols.

[20] Luo S., Du H., Kebede H., Liu Y., Xing F. (2021). Contamination status of major mycotoxins in agricultural products and foodstuffs in Europe. Food Control.

[21] Pandey A.K., Samota M.K., Kumar A., Silva A.S., Dubey N.K. (2023). Fungal Mycotoxins in Food Commodities: Present Status and Future Concerns. Front. Sustain. Food Syst..

[22] Johns L.E., Bebber D.P., Gurr S.J., Brown N.A. (2022). Emerging health threat and cost of *Fusarium* mycotoxins in European wheat. Nat. Food.

[23] Eskola M., Kos G., Elliott C.T., Hajslova J., Mayar S., Krska R. (2020). Worldwide contamination of food-crops with mycotoxins: Validity of the widely cited ‘FAO estimate’ of 25%. Crit. Rev. Food Sci. Nutr..

[24] Ostry V., Malir F., Toman J., Grosse Y. (2017). Mycotoxins as human carcinogens—The IARC Monographs classification. Mycotoxin Res..

[25] Kamle M., Mahato D.K., Devi S., Lee K.E., Kang S.G., Kumar P. (2019). Fumonisins: Impact on agriculture, food, and human health and their management strategies. Toxins.

[26] Leslie J.F., Moretti A., Mesterházy Á., Ameye M., Audenaert K., Singh P.K., Richard-Forget F., Chulze S.N., Ponte E.M.D., Chala A. (2021). Key Global Actions for Mycotoxin Management in Wheat and Other Small Grains. Toxins.

[27] Fisher M.C., Hawkins N.J., Sanglard D., Gurr S.J. (2018). Worldwide emergence of resistance to antifungal drugs challenges human health and food security. Science.

[28] Kumar P., Mahato D.K., Kamle M., Mohanta T.K., Kang S.G. (2017). Aflatoxins: A Global Concern for Food Safety, Human Health and Their Management. Front. Microbiol..

[29] Sarmast E., Fallah A.A., Jafari T., Khaneghah A.M. (2021). Occurrence and fate of mycotoxins in cereals and cereal-based products: A narrative review of systematic reviews and meta-analyses studies. Curr. Opin. Food Sci..

[30] Yu J., Pedroso I.R. (2023). Mycotoxins in Cereal-Based Products and Their Impacts on the Health of Humans, Livestock Animals and Pets. Toxins.

[31] Kolawole O., Siri-Anusornsak W., Petchkongkaew A., Elliott C. (2024). A systematic review of global occurrence of emerging mycotoxins in crops and animal feeds, and their toxicity in livestock. Emerg. Contam..

[32] Khaneghah A.M., Fakhri Y., Gahruie H.H., Niakousari M., Sant’Ana A.S. (2019). Mycotoxins in cereal-based products during 24 years (1983–2017): A global systematic review. Trends Food Sci. Technol..

[33] Wang J., Sufar E.K., Bernhoft A., Seal C., Rempelos L., Hasanaliyeva G., Zhao B., Iversen P.O., Baranski M., Volakakis N. (2024). Mycotoxin Contamination in Organic and Conventional Cereal Grain and Products: A Systematic Literature Review and Meta-Analysis. Compr. Rev. Food Sci. Food Saf..

[34] Sá S.V.M., Monteiro C., Fernandes J.O., Pinto E., Faria M.A., Cunha S.C. (2023). Emerging mycotoxins in infant and children foods: A review. Crit. Rev. Food Sci. Nutr..

[35] Hassan S.E.-D., Salem S.S., Fouda A., Awad M.A., El-Gamal M.S., Abdo A. (2018). New Approach for Antimicrobial Activity and Bio-Control of Various Pathogens by Biosynthesized Copper Nanoparticles Using Endophytic Actinomycetes. J. Radiat. Res. Appl. Sci..

[36] Schabo D.C., Alvarenga V.O., Schaffner D.W., Magnani M. (2021). A worldwide systematic review, meta-analysis, and health risk assessment study of mycotoxins in beers. Compr. Rev. Food Sci. Food Saf..

[37] Narváez A., Rodríguez-Carrasco Y., Castaldo L., Izzo L., Graziani G., Ritieni A. (2020). Occurrence and Exposure Assessment of Mycotoxins in Ready-to-Eat Tree Nut Products through Ultra-High Performance Liquid Chromatography Coupled with High Resolution Q-Orbitrap Mass Spectrometry. Metabolites.

[38] González-Curbelo M.Á., Kabak B. (2023). Occurrence of mycotoxins in dried fruits worldwide, with a focus on aflatoxins and ochratoxin A: A review. Toxins.

[39] Azaiez I., Font G., Mañes J., Fernández-Franzón M. (2015). Survey of mycotoxins in dates and dried fruits from Tunisian and Spanish markets. Food Control.

[40] Abdallah M.F., Krska R., Sulyok M. (2018). Occurrence of ochratoxins, fumonisin B_2_, aflatoxins (B_1_ and B_2_), and other secondary fungal metabolites in dried date palm fruits from Egypt: A mini survey. J. Food Sci..

[41] Palumbo J.D., O’Keeffe T.L., Ho Y.S., Santillan C.J. (2015). Occurrence of Ochratoxin a Contamination and Detection of Ochratoxigenic *Aspergillus* Species in Retail Samples of Dried Fruits and Nuts. J. Food Prot..

[42] Rahimi E., Shakerian A. (2013). Ochratoxin A in Dried Figs, Raisins, Apricots, and Dates on Iranian Retail Market. Health.

[43] Iqbal S.Z., Mehmood Z., Asi M.R., Shahid M., Sehar M., Malik N. (2018). Co-occurrence of aflatoxins and ochratoxin A in nuts, dry fruits, and nutty products. J. Food Saf..

[44] Calderari T.O., Iamanaka B.T., Frisvad J.C., Pitt J.I., Sartori D., Pereira J.L., Fungaro M.H.P., Taniwaki M.H. (2013). The Biodiversity of *Aspergillus* Section *Flavi* in Brazil Nuts: From Rainforest to Consumer. Int. J. Food Microbiol..

[45] Russell R., Paterson M., Lima N., Taniwak M.H. (2014). Coffee, mycotoxins and climate change. Food Res. Int..

[46] Galarce-Bustos O., Alvarado M., Vega M., Aranda M. (2014). Occurrence of ochratoxin A in roasted and instant coffees in Chilean market. Food Control.

[47] Vieira T., Cunha S., Casal S., Preedy V.R. (2015). Mycotoxins in Coffee. Chapter 25. Coffee in Health and Disease Prevention.

[48] Bessaire T., Perrin I., Tarres A., Bebius A., Reding F., Theurillat V. (2019). Mycotoxins in green coffee: Occurrence and risk assessment. Food Control.

[49] Vecchio A., Mineo V., Planeta D. (2012). Ochratoxin A in Instant Coffee in Italy. Food Control.

[50] Al Attiya W., Ul Hassan Z., Al-Thani R., Jaoua S. (2021). Prevalence of toxigenic fungi and mycotoxins in Arabic coffee (*Coffea arabica*): Protective role of traditional coffee roasting, brewing and bacterial volatiles. PLoS ONE.

[51] Copetti M.V., Iamanaka B.T., Pitt J.I., Taniwaki M.H. (2014). Fungi and Mycotoxins in Cocoa: From Farm to Chocolate. Int. J. Food Microbiol..

[52] Pickova D., Ostry V., Malir J., Toman J., Malir F.A. (2020). Review on Mycotoxins and Microfungi in Spices in the Light of the Last Five Years. Toxins.

[53] Kabak B., Dobson A.D.W. (2017). Mycotoxins in spices and herbs—An update. Crit. Rev. Food Sci. Nutr..

[54] El Darra N., Gambacorta L., Solfrizzo M. (2019). Multimycotoxins Occurrence in Spices and Herbs Commercialized in Lebanon. Food Control.

[55] Cighir A., Curticăpean A., Mare A.D., Cighir T., Gabor M.R., Toma F., Man A. (2023). Fungal and Mycotoxin Contamination of Green Leaf Spices Commercialized in Romania: A Food Choice Perspective. Sustainability.

[56] Benkerroum N. (2016). Mycotoxins in dairy products: A review. Int. Dairy J..

[57] Flores-Flores M.E., Lizarraga E., López de Cerain A., González-Peñas E. (2015). Presence of mycotoxins in animal milk: A review. Food Control.

[58] Leite M., Freitas A., Barbosa J., Ramos F. (2023). Regulated and Emerging Mycotoxins in Bulk Raw Milk: What Is the Human Risk?. Toxins.

[59] Becker-Algeri T.A., Castagnaro D., de Bortoli K., de Souza C., Drunkler D.A., Badiale-Furlong E. (2016). Mycotoxins in bovine milk and dairy products: A review. J. Food Sci..

[60] Hocking A.D., Leong S.L., Kazi B.A., Emmett R.W., Scott E.S. (2007). Fungi and Mycotoxins in Vineyards and Grape Products. Int. J. Food Microbiol..

[61] Kollia E., Kanapitsas A., Markaki P. (2014). Occurrence of aflatoxin B1 and ochratoxin A in dried vine fruits from Greek market. Food Addit. Contam. Part B.

[62] Paterson R.R.M., Venâncio A., Lima N., Guilloux-Bénatier M., Rousseaux S. (2018). Predominant Mycotoxins, Mycotoxigenic Fungi and Climate Change Related to Wine. Food Res. Int..

[63] Mateo F., Tarazona A., Gavara R., Mateo E.M. (2025). Bioactive Films with Essential Oils and Machine Learning for Controlling *Aspergillus niger* Growth and Fumonisin B_2_ Production In Vitro. Int. J. Food Microbiol..

[64] Rodrigues P., Silva D., Costa P., Abrunhosa L., Venâncio A., Teixeira A. (2019). Mycobiota and mycotoxins in Portuguese pork, goat and sheep dry-cured hams. Mycotoxin Res..

[65] Merla C., Andreoli G., Garino C., Vicari N., Tosi G., Guglielminetti M.L., Moretti A., Biancardi A., Arlorio M., Fabbi M. (2018). Monitoring of Ochratoxin A and Ochratoxin-Producing Fungi in Traditional Salami Manufactured in Northern Italy. Mycotoxin Res..

[66] Abd El-Tawab A.A., El-Diasty E.M., Khater D.F., Al-baaly Y.M. (2020). Mycological identification of some fungi isolated from meat products and spices with molecular identification of some *Penicillium isolates*. Adv. Anim. Vet. Sci..

[67] Toman J., Pickova D., Rejman L., Ostry V., Malir F. (2024). Investigation of ochratoxin A in air-dry-cured hams. Meat Sci..

[68] Lešić T., Zadravec M., Zdolec N., Vulić A., Perković I., Škrivanko M., Kudumija N., Jakopović Ž., Pleadin J. (2021). Mycobiota and Mycotoxin Contamination of Traditional and Industrial Dry-Fermented Sausage Kulen. Toxins.

[69] Alkuwari A., Hassan Z.U., Zeidan R., Al-Thani R., Jaoua S. (2022). Occurrence of mycotoxins and toxigenic fungi in cereals and application of yeast volatiles for their biological control. Toxins.

[70] Hassan Z.U., Al-Thani R.F., Migheli Q., Jaoua S. (2018). Detection of Toxigenic Mycobiota and Mycotoxins in Cereal Feed Market. Food Control.

[71] Susca A., Villani A., Moretti A., Stea G., Logrieco A. (2020). Identification of toxigenic fungal species associated with maize ear rot: Calmodulin as a single informative gene. Int. J. Food Microbiol..

[72] (2023). Commission Regulation (EU) 2023/915 of 25 April 2023 on maximum levels for certain contaminants in food and repealing Regulation (EC) No 1881/2006. Off. J. Eur. Union.

[73] (2024). Commission Regulation (EU) 2024/1022 of 8 April 2024 amending Regulation (EU) 2023/915 as regards maximum levels of deoxynivalenol in food. Off. J. Eur. Union.

[74] (2024). Commission Regulation (EU) 2024/1038 of 9 April 2024 amending Regulation (EU) 2023/915 as regards maximum levels of T-2 and HT-2 toxins in food. Off. J. Eur. Union.

[75] Gruber-Dorninger C., Novak B., Nagl V., Berthiller F. (2017). Emerging Mycotoxins: Beyond Traditionally Determined Food Contaminants. J. Agric. Food Chem..

[76] Perrone G., Ferrara M., Medina A., Pascale M., Magan N. (2020). Toxigenic Fungi and Mycotoxins in a Climate Change Scenario: Ecology, Genomics, Distribution, Prediction and Prevention of the Risk. Microorganisms.

[77] Moretti A., Pascale M., Logrieco A.F. (2019). Mycotoxin Risks under a Climate Change Scenario in Europe. Trends Food Sci. Technol..

[78] Casu A., Leggieri M.C., Toscano P., Battilani P. (2024). Changing Climate, Shifting Mycotoxins: A Comprehensive Review of Climate Change Impact on Mycotoxin Contamination. Compr. Rev. Food Sci. Food Saf..

[79] Battilani P., Toscano P., Van Der Fels-Klerx H.J., Moretti A., Leggieri M.C., Brera C., Rortais A., Goumperis T., Robinson T. (2016). Aflatoxin B1 contamination in maize in Europe increases due to climate change. Sci. Rep..

[80] Lv C., Jin J., Wang P., Dai X., Liu Y., Zheng M., Xing F. (2019). Interaction of water activity and temperature on the growth, gene expression, and aflatoxin production by *Aspergillus flavus* on paddy and polished rice. Food Chem..

[81] Al-Zaban M.I. (2023). Impacts of temperature and water activity interactions on growth, aflatoxin B1 production and expression of major biosynthetic genes of AFB_1_ in *Aspergillus flavus* isolates. Microorganisms.

[82] Kifer D., Jakšić D., Šegvić Klarić M. (2020). Assessing the effect of mycotoxin combinations: Which mathematical model is (the most) appropriate?. Toxins.

[83] Smith M.C., Madec S., Coton E., Hymery N. (2016). Natural Co-occurrence of Mycotoxins in Foods and Feeds and their in vitro Combined Toxicological Effects. Toxins.

[84] Peijers G.J.A., Speijers M.H.M. (2004). Combined Toxic Effects of Mycotoxins. Toxicol. Lett..

[85] Venkatesh N., Keller N.P. (2019). Mycotoxins in Conversation with Bacteria and Fungi. Front. Microbiol..

[86] Assunção R., Silva M.J., Alvito P. (2016). Challenges in risk assessment of multiple mycotoxins in food. World Mycotoxin J..

[87] Nji Q.N., Babalola O.O., Ekwomadu T.I., Nleya N., Mwanza M. (2022). Six Main Contributing Factors to High Levels of Mycotoxin Contamination in African Foods. Toxins.

[88] Hamad G.M., Mehany T., Simal-Gandara J., Abou-Alella S., Esua O.J., Abdel-Wahhab M.A., Hafez E.E. (2023). A Review of Recent Innovative Strategies for Controlling Mycotoxins in Foods. Food Control.

[89] Kabak B. (2009). The fate of mycotoxins during thermal food processing. J. Sci. Food Agric..

[90] Inglis A., Parnell A.C., Subramani N., Doohan F.M. (2024). Machine Learning Applied to the Detection of Mycotoxin in Food: A Systematic Review. Toxins.

[91] Tarazona A., Mateo E.M., Gómez J.V., Romera D., Mateo F. (2021). Potential use of machine learning methods in assessment of *Fusarium culmorum* and *Fusarium proliferatum* growth and mycotoxin production in treatments with antifungal agents. Fungal Biol..

[92] Tarazona A., Mateo E.M., Gómez J.V., Gavara R., Jiménez M., Mateo F. (2021). Machine learning approach for predicting *Fusarium culmorum* and *F. proliferatum* growth and mycotoxin production in treatments with ethylene-vinyl alcohol copolymer films containing pure components of essential oils. Int. J. Food Microbiol..

[93] Mateo E.M., Gómez J.V., Tarazona A., García-Esparza M.A., Mateo F. (2021). Comparative Analysis of Machine Learning Methods to Predict Growth of *F. sporotrichioides* and Production of T-2 and HT-2 Toxins in Treatments with Ethylene-Vinyl Alcohol Films Containing Pure Components of Essential Oils. Toxins.

[94] Mateo E.M., Tarazona A., Jiménez M., Mateo F. (2022). Lactic Acid Bacteria as Potential Agents for Biocontrol of Aflatoxigenic and Ochratoxigenic Fungi. Toxins.

[95] Mateo E.M., Tarazona A., Aznar R., Mateo F. (2023). Exploring the Impact of Lactic Acid Bacteria on the Biocontrol of Toxigenic *Fusarium* spp. and Their Main Mycotoxins. Int. J. Food Microbiol..

[96] Mateo F., Gadea R., Mateo E.M., Jiménez M. (2011). Multilayer Perceptron Neural Networks and Radial-Basis Function Networks as Tools to Forecast Accumulation of Deoxynivalenol in Barley Seeds Contaminated with *Fusarium culmorum*. Food Control.

[97] Wang X., Liu C., van der Fels-Klerx H.J. (2022). Regional Prediction of Multi-Mycotoxin Contamination of Wheat in Europe Using Machine Learning. Food Res. Int..

[98] Aggarwal A., Mishra A., Tabassum N., Kim Y.-M., Khan F. (2024). Detection of mycotoxin contamination in foods using artificial intelligence: A review. Foods.

[99] Intergovernmental Panel on Climate Change (IPCC) Climate Change 2023: Sixth Assessment Report (AR6). The Synthesis Report Is Based on the Content of the Three Working Groups Assessment Reports: WGI–The Physical Science Basis, WGII—Impacts, Adaptation and Vulnerability, WGIII—Mitigation of Climate Change. Geneva, Switzerland. https://www.ipcc.ch/report/sixth-assessment-report-cycle.

[100] Waheed A., Haxim Y., Islam W., Ahmad M., Muhammad M., Alqahtani F.M., Hashem M., Salih H., Zhang D. (2023). Climate Change Reshaping Plant-Fungal Interaction. Environ. Res..

[101] Lahlali R., Taoussi M., Laasli S.E., Gachara G., Ezzouggari R., Belabess Z., Aberkani K., Assouguem A., Meddich A., El Jarroudi M. (2024). Effects of Climate Change on Plant Pathogens and Host-Pathogen Interactions. Crop Environ..

[102] Liu Y., Yamdeu J.H.G., Gong Y.Y., Orfila C. (2020). A review of postharvest approaches to reduce fungal and mycotoxin contamination of foods. Compr. Rev. Food Sci. Food Saf..

[103] Mateo F., Mateo E.M., Tarazona A., García-Esparza M.Á., Soria J.M., Jiménez M. (2025). New Strategies and Artificial Intelligence Methods for the Mitigation of Toxigenic Fungi and Mycotoxins in Foods. Toxins.

[104] Brauer V.S., Rezende C.P., Pessoni A.M., De Paula R.G., Rangappa K.S., Nayaka S.C., Gupta V.K., Almeida F. (2019). Antifungal Agents in Agriculture: Friends and Foes of Public Health. Biomolecules.

[105] Marín P., de Ory A., Cruz A., Magan N., González-Jaén M.T. (2013). Potential effects of environmental conditions on the efficiency of the antifungal tebuconazole controlling *Fusarium verticillioides* and *Fusarium proliferatum* growth rate and fumonisin biosynthesis. Int. J. Food Microbiol..

[106] Mateo E.M., Valle-Algarra F.M., Mateo R., Jiménez M., Magan N. (2011). Effect of Fenpropimorph, Prochloraz and Tebuconazole on Growth and Production of T-2 and HT-2 Toxins by *Fusarium langsethiae* in Oat-Based Medium. Int. J. Food Microbiol..

[107] Vitiello A., Ferrara F., Boccellino M., Ponzo A., Cimmino C., Comberiati E., Zovi A., Clemente S., Sabbatucci M. (2023). Antifungal Drug Resistance: An Emergent Health Threat. Biomedicines.

[108] Cui X., Wang L., Lü Y., Yue C. (2022). Development and Research Progress of Anti-Drug Resistant Fungal Drugs. J. Infect. Public Health.

[109] Hahn M. (2014). The Rising Threat of Fungicide Resistance in Plant Pathogenic Fungi: *Botrytis* as a Case Study. J. Chem. Biol..

[110] Ribas A.D.R.E., Spolti P., Del Ponte E.M., Donato K.Z., Schrekker H., Fuentefria A.M. (2016). Is the emergence of fungal resistance to medical triazoles related to their use in the agroecosystems?. Braz. J. Microbiol..

[111] Robbins N., Caplan T., Cowen L.E. (2017). Molecular evolution of antifungal drug resistance. Annu. Rev. Microbiol..

[112] European Food Safety Authority (EFSA), European Centre for Disease Prevention and Control (ECDC), European Chemicals Agency (ECHA), European Environment Agency (EEA), European Medicines Agency (EMA), European Commission’s Joint Research Centre (JRC) (2025). Impact of the use of azole fungicides, other than as human medicines, on the development of azole-resistant *Aspergillus* spp.. EFSA J..

[113] Revie N.M., Iyer K.R., Robbins N., Cowen L.E. (2018). Antifungal drug resistance: Evolution, mechanisms and impact. Curr. Opin. Microbiol..

[114] Hokken M.W., Zwaan B.J., Melchers W.J., Verweij P.E. (2019). Facilitators of Adaptation and Antifungal Resistance Mechanisms in Clinically Relevant Fungi. Fungal Genet. Biol..

[115] Habschied K., Krstanović V., Zdunić Z., Babić J., Mastanjević K., Šarić G.K. (2021). Mycotoxins Biocontrol Methods for Healthier Crops and Stored Products. J. Fungi.

[116] Spanic V., Zdunić Z., Drezner G., Sarkanj B. (2019). The Pressure of Fusarium Disease and Its Relation with Mycotoxins in the Wheat Grain and Malt. Toxins.

[117] Gomes A.S.d.L.P.B., Weber S.H., Luciano F.B. (2024). Resistance of Transgenic Maize Cultivars to Mycotoxin Production—Systematic Review and Meta-Analysis. Toxins.

[118] Huang T., Li X., Maier M., O’Brien-Simpson N.M., Heath D.E., O’Connor A.J. (2023). Using Inorganic Nanoparticles to Fight Fungal Infections in the Antimicrobial Resistant Era. Acta Biomater..

[119] Peters R., Brandhoff P., Weigel S., Marvin H., Bouwmeester H., Aschberger K., Rauscher H., Amenta V., Arena M., Moniz F.B. (2014). Inventory of Nanotechnology Applications in the Agricultural, Feed and Food Sector. EFSA Support. Publ..

[120] Chen J., Guo Y., Zhang X., Liu J., Gong P., Su Z., Fan L., Li G. (2023). Emerging Nanoparticles in Food: Sources, Application, and Safety. J. Agric. Food Chem..

[121] Singh S., Chaurasia P.K., Bharat S.L. (2022). Functional roles of essential oils as an effective alternative of synthetic food preservatives: A review. Bharat J. Food Process Preserv..

[122] Atanda S.A., Shaibu R.O., Agunbiade F.O. (2025). Nanoparticles in agriculture: Balancing food security and environmental sustainability. Discov. Agric..

[123] Dangi K., Verma A.K. (2021). Efficient and Eco-friendly Smart Nano-pesticides: Emerging Prospects for Agriculture. Mater. Today Proc..

[124] Kopittke P.M., Lombi E., Wang P., Schjoerring J.K., Husted S. (2019). Nanomaterials as fertilizers for improving plant mineral nutrition and environmental outcomes. Environ. Sci..

[125] Santás-Miguel V., Arias-Estévez M., Rodríguez-Seijo A., Arenas-Lago D. (2023). Use of metal nanoparticles in agriculture. A review on the effects on plant germination. Environ. Pollut..

[126] Mansoor S., Zahoor I., Baba T.R., Padder S.A., Bhat Z.A., Koul A.M., Jiang L. (2021). Fabrication of silver nanoparticles against fungal pathogens. Front. Nanotechnol..

[127] Kaningini A.G., Nelwamondo A.M., Azizi S., Maaza M., Mohale K.C. (2022). Metal nanoparticles in agriculture: A review of possible use. Coatings.

[128] Jaskulski D., Jaskulska I., Majewska J., Radziemska M., Bilgin A., Brtnicky M. (2022). Silver nanoparticles (AgNPs) in urea solution in laboratory tests and field experiments with crops and vegetables. Materials.

[129] Zahra Z., Habib Z., Chung S., Badshah M.A. (2020). Exposure Route of TiO2 NPs from Industrial Applications to Wastewater Treatment and Their Impacts on the Agro-Environment. Nanomaterials.

[130] Dey S., Ghosh N., Nath S., Gopal G., Paul S., Mukherjee A., Paul S., Kundu R. (2024). Application of multi-metallic nanoparticles in agriculture: The more, the better?. Biocatal. Agric. Biotechnol..

[131] Singh R.P., Handa R., Manchanda G. (2021). Nanoparticles in sustainable agriculture: An emerging opportunity. J. Control. Release.

[132] Mgadi K., Ndaba B., Roopnarain A., Rama H., Adeleke R. (2024). Nanoparticle Applications in Agriculture: Overview and Response of Plant-Associated Microorganisms. Front. Microbiol..

[133] Su C., Chen A., Liang W., Xie W., Xu X., Zhan X., Zhang W., Peng C. (2024). Copper-based nanomaterials: Opportunities for sustainable agriculture. Sci. Total Environ..

[134] Cruz-Luna A.R., Cruz-Martínez H., Vásquez-López A., Medina D.I. (2021). Metal Nanoparticles as Novel Antifungal Agents for Sustainable Agriculture: Current Advances and Future Directions. J. Fungi.

[135] Cruz-Luna A.R., Vásquez-López A., Rojas-Chávez H., Valdés-Madrigal M.A., Cruz-Martínez H., Medina D.I. (2023). Engineered Metal Oxide Nanoparticles as Fungicides for Plant Disease Control. Plants.

[136] Kutawa A.B., Ahmad K., Ali A., Hussein M.Z., Abdul Wahab M.A., Adamu A., Ismaila A.A., Gunasena M.T., Rahman M.Z., Hossain M.I. (2021). Trends in Nanotechnology and Its Potentialities to Control Plant Pathogenic Fungi: A Review. Biology.

[137] Nguyen N.N., Nguyen N.T., Nguyen P.T., Phan Q.N., Le T.L., Do H.D.K. (2024). Current and Emerging Nanotechnology for Sustainable Development of Agriculture: Implementation Design Strategy and Application. Heliyon.

[138] Baker S., Volova T., Prudnikova S.V., Satish S., Prasad M.N.N. (2017). Nanoagroparticles emerging trends and future prospect in modern agriculture system. Environ. Toxicol. Pharmacol..

[139] Adisa I.O., Pullagurala V.L.R., Peralta-Videa J.R., Dimkpa C.O., Elmer W.H., Gardea-Torresdey J.L., White J.C. (2019). Recent advances in nano-enabled fertilizers and pesticides: A critical review of mechanisms of action. Environ. Sci. Nano.

[140] Agrimonti C., Lauro M., Visioli G. (2021). Smart agriculture for food quality: Facing climate change in the 21st century. Crit. Rev. Food Sci. Nutr..

[141] Castro-Mayorga J.L., Cabrera-Villamizar L., Balcucho-Escalante J., Fabra M.J., López-Rubio A., Rajendran S., Mukherjee A., Nguyen T.A., Godugu C., Shukla R.K. (2020). Applications of Nanotechnology in Agri-Food Productions. Nanotoxicity.

[142] Mishra S., Keswani C., Abhilash P.C., Fraceto L.F., Singh H.B. (2017). Integrated Approach of Agri-nanotechnology: Challenges and Future Trends. Front. Plant Sci..

[143] Tanwar A., Role S. (2019). Role and Effects of Nanotechnology Used in Pesticides and Agriculture Field. AIP Conf. Proc..

[144] Siddique S., Chow J.C. (2020). Gold nanoparticles for drug delivery and cancer therapy. Appl. Sci..

[145] Puttasiddaiah R., Basavegowda N., Lakshmanagowda N.K., Raghavendra V.B., Sagar N., Sridhar K., Dikkala P.K., Bhaswant M., Baek K.-H., Sharma M. (2025). Emerging Nanoparticle-Based Diagnostics and Therapeutics for Cancer: Innovations and Challenges. Pharmaceutics.

[146] Islam F., Shohag S., Uddin M.J., Islam M.R., Nafady M.H., Akter A., Mitra S., Roy A., Emran T.B., Cavalu S. (2022). Exploring the journey of zinc oxide nanoparticles (ZnO-NPs) toward biomedical applications. Materials.

[147] Zhu C., Ji Z., Ma J., Ding Z., Shen J., Wang Q. (2021). Recent Advances of Nanotechnology-Facilitated Bacteria-Based Drug and Gene Delivery Systems for Cancer Treatment. Pharmaceutics.

[148] Yetisgin A.A., Cetinel S., Zuvin M., Kosar A., Kutlu O. (2020). Therapeutic Nanoparticles and Their Targeted Delivery Applications. Molecules.

[149] Hofmann-Amtenbrink M., Grainger D.W., Hofmann H. (2015). Nanoparticles in Medicine: Current Challenges Facing Inorganic Nanoparticle Toxicity Assessments and Standardizations. Nanomedicine.

[150] Lam E., Luong J.H. (2014). Carbon Materials as Catalyst Supports and Catalysts in the Transformation of Biomass to Fuels and Chemicals. ACS Catal..

[151] Rassaei L., Marken F., Sillanpää M., Amiri M., Cirtiu C.M., Sillanpää M. (2011). Nanoparticles in electrochemical sensors for environmental monitoring. TrAC Trends Anal. Chem..

[152] Khan F., Shahid A., Zhu H., Wang N., Javed M.R., Ahmad N., Xu J., Alam M.A., Mehmood M.A. (2022). Prospects of algae-based green synthesis of nanoparticles for environmental applications. Chemosphere.

[153] Rasheed T., Bilal M., Li C., Nabeel F., Khalid M., Iqbal H.M.N. (2018). Catalytic Potential of Bio-Synthesized Silver Nanoparticles Using *Convolvulus arvensis* Extract for the Degradation of Environmental Pollutants. J. Photochem. Photobiol. B Biol..

[154] Xiong Y., Li W., Wen Q., Xu D., Ren J., Lin Q. (2022). Aptamer-Engineered Nanomaterials to Aid in Mycotoxin Determination. Food Control.

[155] Rai P.K., Kumar V., Lee S.S., Raza N., Kim K.-H., Ok Y.S., Tsang D.C.W. (2018). Nanoparticle-Plant Interaction: Implications in Energy, Environment, and Agriculture. Environ. Int..

[156] Altammar K.A. (2023). A review on nanoparticles: Characteristics, synthesis, applications, and challenges. Front. Microbiol..

[157] Wasilewska A., Bielicka M., Klekotka U., Kalska-Szostko B. (2023). Nanoparticle Applications in Food—A Review. Food Funct..

[158] Horky P., Skalickova S., Baholet D., Skladanka J. (2018). Nanoparticles as a Solution for Eliminating the Risk of Mycotoxins. Nanomaterials.

[159] Boholm M., Arvidsson R. (2016). A definition framework for the terms nanomaterial and nanoparticle. Nanoethics.

[160] Zain M., Yasmeen H., Yadav S.S., Amir S., Bilal M., Shahid A., Khurshid M., Denizli A., Nguyen T.A., Rajan M., Alam M.F., Rahman K. (2022). Applications of Nanotechnology in Biological Systems and Medicine. Micro and Nano Technologies, Nanotechnology for Hematology, Blood Transfusion, and Artificial Blood.

[161] Mitchell M.J., Billingsley M.M., Haley R.M., Wechsler M.E., Peppas N.M., Langer R. (2021). Engineering Precision Nanoparticles for Drug Delivery. Nat. Rev. Drug Discov..

[162] (2022). Commission Recommendation of 10 June 2022 on the definition of nanomaterial (Text with EEA relevance) (2022/C 229/01) (C/2022/3689). Off. J. Eur. Union.

[163] (2011). Commission Recommendation of 18 October 2011 on the definition of nanomaterial (Text with EEA relevance) (2011/696/EU). Off. J. Eur. Union.

[164] (2015). Commission Regulation (EU) 2015/2283 of the European Parliament and of the Council of 25 November 2015 on novel foods, amending Regulation (EU) No 1169/2011 and repealing Regulation (EC) No 258/97 and Commission Regulation (EC) No 1852/2001. Off. J. Eur. Union.

[165] Joudeh N., Linke D. (2022). Nanoparticle classification, physicochemical properties, characterization, and applications: A comprehensive review for biologists. J. Nanobiotechnol..

[166] Erdoğan N., Akkın S., Bilensoy E. (2019). Nanocapsules for Drug Delivery: An Updated Review of the Last Decade. Recent Pat. Drug Deliv. Formul..

[167] Ozuna-Valencia K.H., Moreno-Vásquez M.J., Graciano-Verdugo A.Z., Rodríguez-Félix F., Robles-García M.Á., Barreras-Urbina C.G., Quintero-Reyes I.E., Cornejo-Ramírez Y.I., Tapia-Hernández J.A. (2024). The Application of Organic and Inorganic Nanoparticles Incorporated in Edible Coatings and Their Effect on the Physicochemical and Microbiological Properties of Seafood. Processes.

[168] Dhaka A., Mali S.C., Sharma S., Trivedi R.A. (2023). Review on Biological Synthesis of Silver Nanoparticles and Their Potential Applications. Results Chem..

[169] Nami S., Aghebati-Maleki A., Aghebati-Maleki L. (2021). Current Applications and Prospects of Nanoparticles for Antifungal Drug Delivery. EXCLI J..

[170] Ealia S.A., Saravanakumar M. (2017). A Review on the Classification, Characterization, Synthesis of Nanoparticles and Their Application. IOP Conf. Ser. Mater. Sci. Eng..

[171] Rajput V.D., Singh A., Minkina T., Rawat S., Mandzhieva S., Sushkova S., Shuvaeva V., Nazarenko O., Rajput P., Komariah (2021). Nano-Enabled Products: Challenges and Opportunities for Sustainable Agriculture. Plants.

[172] Abd-Elsalam K.A., Hashim A.F., Alghuthaymi M.A., Said-Galiev E., Grumezescu A.M. (2017). Nanobiotechnological strategies for toxigenic fungi and mycotoxin control. Nanotechnology in the Agri-Food Industry. Food Preservation.

[173] Kheiri A., Moosawi Jorf S.A., Malihipour A., Saremi H., Nikkhah M. (2017). Synthesis and characterization of chitosan nanoparticles and their effect on *Fusarium* head blight and oxidative activity in wheat. Int. J. Biol. Macromol..

[174] Hassanein M.M.M., Abdel-Razek A.G., Al-Amrousi E.F., Badr A.N. (2023). Application of lime peel oil composite nanoemulsion to prevent toxigenic fungi in nuts. Heliyon.

[175] Almawash S. (2023). Solid lipid nanoparticles, an effective carrier for classical antifungal drugs. Saudi Pharm. J..

[176] Vogel T., Kohlmann S., Abboud Z., Thusek S., Fella F., Teßmar J., Sekimizu K., Miyashita A., Beilhack A., Groll J. (2024). Beyond the Charge: Interplay of Nanogels’ Functional Group and Zeta-Potential for Antifungal Drug Delivery to Human Pathogenic Fungus *Aspergillus fumigatus*. Macromol. Biosci..

[177] Mosallam S., Albash R., Abdelbari M.A. (2022). Advanced Vesicular Systems for Antifungal Drug Delivery. AAPS PharmSciTech.

[178] Soe H.M.S.H., Maw P.D., Loftsson T., Jansook P. (2022). A Current Overview of Cyclodextrin-Based Nanocarriers for Enhanced Antifungal Delivery. Pharmaceuticals.

[179] Dariusz T., Młynarczyk D.T., Długaszewska J., Kałużna-Młynarczyk A., Gosliński T. (2021). Dendrimers against fungi—A state of the art review. J. Control. Release.

[180] Wei H., Mao J., Sun D., Zhang Q., Cheng L., Yang X., Li P. (2023). Strategies to control mycotoxins and toxigenic fungi contamination by nano-semiconductor in food and agro-food: A review. Crit. Rev. Food Sci. Nutr..

[181] Hu X., Li H., Yang J., Wen X., Wang S., Pan M. (2023). Nanoscale Materials Applying for the Detection of Mycotoxins in Foods. Foods.

[182] Thirugnanasambandan T., Gopinath S.C.B. (2023). Nanomaterials in Food Industry for the Protection from Mycotoxins: An Update. Biotech.

[183] Mali R., Patil J. (2023). Nanoparticles: A novel antifungal drug delivery system. Mater. Proc..

[184] Irshad M.A., Hussain A., Nasim I., Nawaz R., Azeem S., Al-Mutairi A.A., Al-Hussain S.A., Zaki M.E.A. (2024). Exploring the antifungal activities of green nanoparticles for sustainable agriculture: A research update. Chem. Biol. Technol. Agric..

[185] Chen J., Wu L., Lu M., Lu S., Li Z., Ding W. (2020). Comparative Study on the Fungicidal Activity of Metallic MgO Nanoparticles and Macroscale MgO Against Soilborne Fungal Phytopathogens. Front. Microbiol..

[186] Jian Y., Chen X., Ahmed T., Shang Q., Zhang S., Ma Z., Yin Y. (2021). Toxicity and action mechanisms of silver nanoparticles against the mycotoxin-producing fungus *Fusarium graminearum*. J. Adv. Res..

[187] Kandi V., Kandi S. (2015). Antimicrobial properties of nanomolecules: Potential candidates as antibiotics in the era of multi-drug resistance. Epidemiol. Health.

[188] Jamkhande P.G., Ghule N.W., Bamer A.H., Kalaskar M.G. (2019). Metal nanoparticles synthesis: An overview on methods of preparation, advantages and disadvantages, and applications. J. Drug Deliv. Sci. Technol..

[189] De la Rosa-García S.C., Martínez-Torres P., Gómez-Cornelio S., Corral-Aguado M.A., Quintana P., Gómez-Ortíz N.M. (2018). Antifungal Activity of ZnO and MgO Nanomaterials and Their Mixtures Against *Colletotrichum gloeosporioides* Strains from Tropical Fruit. J. Nanomater..

[190] Arciniegas-Grijalba P.A., Patiño-Portela M.C., Mosquera-Sánchez L.P., Guerrero-Vargas J.A., Rodríguez-Páez J.E. (2017). ZnO nanoparticles (ZnO-NPs) and their antifungal activity against coffee fungus *Erythricium salmonicolor*. Appl. Nanosci..

[191] Slavin Y.N., Bach H. (2022). Mechanisms of Antifungal Properties of Metal Nanoparticles. Nanomaterials.

[192] Abd-Elsalam K.A., El-Naggar M.A., Ghannouchi A., Bouqellah N.A., Rai M., Abd-Elsalam K.A. (2020). Nanomaterials and ozonation: Safe strategies for mycotoxin management. Nanomycotoxicology.

[193] Thipe V.C., Batista J.G.S., Lugão A.B., Abd-Elsalam K.A. (2022). Copper Nanomaterials for Eliminating the Risk of Mycotoxins. Nanobiotechnology for Plant Protection: Copper Nanostructures: Next-Generation of Agrochemicals for Sustainable Agroecosystems.

[194] Bakshi M., Kumar A., Abd-Elsalam K.A. (2022). Applications of copper nanoparticles in plant protection and pollution sensing: Toward promoting sustainable agriculture. Nanobiotechnology for Plant Protection, Copper Nanostructures: Next-Generation of Agrochemicals for Sustainable Agroecosystems.

[195] Dimitrijevic M., Karabasil N., Boskovic M., Teodorovic V., Vasilev D., Djordjevic V., Kilibarda N., Čobanović N. (2015). Safety Aspects of Nanotechnology Applications in Food Packaging. Procedia Food Sci..

[196] Abid N., Khan A.M., Shujait S., Chaudhary K., Ikram M., Imran M., Haider J., Khan M., Khan Q., Maqbool M. (2022). Synthesis of nanomaterials using various top-down and bottom-up approaches, influencing factors, advantages, and disadvantages: A review. Adv. Colloid Interface Sci..

[197] Yadav P.T., Yadav R.M., Singh D.P. (2012). Mechanical Milling: A Top Down Approach for the Synthesis of Nanomaterials and Nanocomposites. Nanosci. Nanotechnol..

[198] Otis G., Ejgenberg M., Mastai Y. (2021). Solvent-Free Mechanochemical Synthesis of ZnO Nanoparticles by High-Energy Ball Milling of ?-Zn(OH)_2_ Crystals. Nanomaterials.

[199] Venkatesh R., Karthi N., Kawin N., Prakash T., Kannan C., Karthigairajan M., Bobe K. (2022). Synthesis and adsorbent performance of modified biochar with Ag/MgO nanocomposites for heat storage application. Adsorpt. Sci. Technol..

[200] Kim M., Osone S., Kim T., Higashi H., Seto T. (2017). Synthesis of Nanoparticles by Laser Ablation: A Review. KONA Powder Partic. J..

[201] Nikolov A.S., Stankova N.E., Karashanova D.B., Nedyalkov N.N., Pavlov E.L., Koev K.T., Najdenski H., Kussovski V., Avramov L.A., Ristoscu C. (2021). Synergistic effect in a two-phase laser procedure for production of silver nanoparticles colloids applicable in ophthalmology. Opt. Laser Technol..

[202] Chugh H., Sood D., Chandra I., Tomar V., Dhawan G., Chandra R. (2018). Role of Gold and Silver Nanoparticles in Cancer Nano-Medicine. Artif. Cells Nanomed. Biotechnol..

[203] Fares A., Mahdy A., Ahmed G. (2024). Unraveling the mysteries of silver nanoparticles: Synthesis, characterization, antimicrobial effects and uptake translocation in plant-a review. Planta.

[204] Nguyen M.T., Deng L., Yonezawa T. (2022). Control of nanoparticles synthesized via vacuum sputter deposition onto liquids: A review. Soft Matter.

[205] Wender H., Migowski P., Feil A.F., Teixeira S.R., Dupont J. (2013). Sputtering deposition of nanoparticles onto liquid substrates: Recent advances and future trends. Coord. Chem. Rev..

[206] Sinha R., Lavrijsen R., Verheijen M.A., Zoethout E., Genuit H., van de Sanden M.C.M., Bieberle-Hütter A. (2019). Electrochemistry of Sputtered Hematite Photoanodes: A Comparison of Metallic DC versus Reactive RF Sputtering. ACS Omega.

[207] Sergievskaya A., Chauvin A., Konstantinidis S. (2022). Sputtering onto liquids: A critical review. Beilstein J. Nanotechnol..

[208] Sergievskaya A., Absil R., Chauvin A., Yusenko K.V., Veselý J., Godfroid T., Konstantinidis S. (2023). Sputtering onto liquids: How does the liquid viscosity affect the formation of nanoparticles and metal films?. Phys. Chem. Chem. Phys..

[209] Leng J., Wang Z., Wang J., Wu H.-H., Yan G., Li X., Guo H., Liu Y., Zhang Q., Guo Z. (2019). Advances in Nanostructures Fabricated via Spray Pyrolysis and Their Applications in Energy Storage and Conversion. Chem. Soc. Rev..

[210] Majerič P., Rudolf R. (2020). Advances in Ultrasonic Spray Pyrolysis Processing of Noble Metal Nanoparticles-Review. Materials.

[211] Debecker D.P., le Bras S., Boissière C., Chaumonnot A., Sánchez C. (2018). Aerosol processing: A wind of innovation in the field of advanced heterogeneous catalysts. Chem. Soc. Rev..

[212] Ko Y.N., Park S.B., Jung K.Y., Kang Y.C. (2013). One-Pot Facile Synthesis of Ant-Cave-Structured Metal Oxide–Carbon Microballs by Continuous Process for Use as Anode Materials in Li-Ion Batteries. Nano Lett..

[213] Workie A.B., Ningsih H.S., Shih S.-J. (2023). A Comprehensive Review on the Spray Pyrolysis Technique: Historical Context, Operational Factors, Classifications, and Product Applications. J. Anal. Appl. Pyrolysis.

[214] Ghaffarian H.R., Saiedi M., Sayyadnejad M.A., Rashidi A.M. (2011). Synthesis of ZnO nanoparticles by spray pyrolysis method. Iran. J. Chem. Chem. Eng. (Int. Engl. Ed.).

[215] Allag N., Bouafia A., Chemsa B., Ben Mya O., Chala A., Siad C., Alam M.W. (2024). Effect of precursors on structural, optical and surface properties of ZnO thin film prepared by spray pyrolysis method: Efficient removal of Cu (II) from wastewater. Transit. Met. Chem..

[216] Sudha M., Madhan A.B., Geethac E., Satheeskumard R. (2024). Synthesis of zinc oxide thin films by spray pyrolysis technique. J. Ovonic Res..

[217] Ozcelik B.K., Ergun C. (2014). Synthesis of ZnO nanoparticles by an aerosol process. Ceram. Int..

[218] Tanhaei A., Mohammadi M., Hamishehkar H., Hamblin M.R. (2021). Electrospraying as a novel method of particle engineering for drug delivery vehicles. J. Control. Release.

[219] Kim M.J., Song J.Y., Hwang S.H., Park D.Y., Park S.M. (2022). Electrospray mode discrimination with current signal using deep convolutional neural network and class activation map. Sci. Rep..

[220] Patel P.R., Haemmerich D. (2024). Review on Electrospray Nanoparticles for Drug Delivery: Exploring Applications. Polym. Adv. Technol..

[221] Burlec A.F., Corciova A., Boev M., Batir-Marin D., Mircea C., Cioanca O., Danila G., Danila M., Bucur A.F., Hancianu M. (2023). Current Overview of Metal Nanoparticles’ Synthesis, Characterization, and Biomedical Applications, with a Focus on Silver and Gold Nanoparticles. Pharmaceuticals.

[222] Chandrakala V., Aruna V., Angajala G. (2022). Review on metal nanoparticles as nanocarriers: Current challenges and perspectives in drug delivery systems. Emergent Mater..

[223] Förster H., Wolfrum C., Peukert W. (2012). Experimental study of metal nanoparticle synthesis by an arc evaporation/condensation process. J. Nanopart. Res..

[224] Markov A.N., Kapinos A.A., Petukhov A.N., Dokin E.S., Emelyanov A.V., Abarbanel N.V., Zarubin D.M., Golovacheva A.A., Suvorov S.S., Barysheva A.V. (2024). Synthesis of Zinc Nanoparticles by the Gas Condensation Method in a Non-Contact Crucible and Their Physical-Chemical Characterization. Nanomaterials.

[225] Raffi M., Rumaiz A.K., Hasan M.M., Shah S.I. (2007). Studies of the growth parameters for silver nanoparticle synthesis by inert gas condensation. J. Mater. Res..

[226] Bokov D., Jalil A.T., Chupradit S., Suksatan W., Ansari M.J., Shewael I.H., Valiev G.H., Kianfar E. (2021). Nanomaterial by Sol-Gel Method: Synthesis and Application. Adv. Mater. Sci. Eng..

[227] Parashar M., Shukla V.K., Singh R. (2020). Metal oxides nanoparticles via sol–gel method: A review on synthesis, characterization and applications. J. Mater. Sci. Mater. Electron..

[228] Chandekar K.V., Shkir M., Khan A., AlFaify S., Hussain C.M., Patankar K.K. (2022). Novel magnetic materials preparation, characterizations and their applications. Woodhead Publishing Series in Electronic and Optical Materials, Fundamentals and Industrial Applications of Magnetic Nanoparticles.

[229] Manawi Y.M., Ihsanullah, Samara A., Al-Ansari T., Atieh M.A. (2018). A Review of Carbon Nanomaterials’ Synthesis via the Chemical Vapor Deposition (CVD) Method. Materials.

[230] Piszczek P., Radtke A., Seehra M.S., Bristow A.D. (2018). Silver Nanoparticles Fabricated Using Chemical Vapor Deposition and Atomic Layer Deposition Techniques: Properties, Applications and Perspectives: Review. Noble and Precious Metals—Properties, Nanoscale Effects and Applications.

[231] Vikulova E.S., Dorovskikh S.I., Basova T.V., Zheravin A.A., Morozova N.B. (2024). Silver CVD and ALD Precursors: Synthesis, Properties, and Application in Deposition Processes. Molecules.

[232] Golrokhi Z., Chalker S., Sutcliffe C.J., Potter R.J. (2016). Self-Limiting Atomic Layer Deposition of Conformal Nanostructured Silver Films. Appl. Surf. Sci..

[233] Singaravelan R., Bangaru Sudarsan Alwar S. (2015). Electrochemical synthesis, characterisation and phytogenic properties of silver nanoparticles. Appl. Nanosci..

[234] Nasretdinova G.R., Fazleeva R.R., Mukhitova R.K., Nizameev I.R., Kadirov M.K., Ziganshina A.Y., Yanilkin V.V. (2015). Electrochemical synthesis of silver nanoparticles in solution. Electrochem. Commun..

[235] Anand V., Harshavardhan, Srivastava V.C. (2015). Synthesis and Characterization of Copper Nanoparticles by Electrochemical Method: Effect of pH. J. Nano Res..

[236] Nur S.U., Anung P., Enny L., Endang S., Hotman L., Triani W., Siska F. (2018). Critical parameters of silver nanoparticles (AgNPs) synthesized by sodium borohydride reduction. Res. J. Chem. Environ..

[237] Nzekwe I.T., Agubata C.O., Umeyor C.E., Okoye I.E., Ogwueleka C.B. (2017). Synthesis of Silver Nanoparticles by Sodium Borohydride Reduction Method: Optimization of Conditions for High Anti-staphylococcal Activity. J. Pharm. Res. Int..

[238] Gómez J.V., Tarazona A., Mateo F., Jiménez M., Mateo E.M. (2019). Potential impact of engineered silver nanoparticles in the control of aflatoxins, ochratoxin A and the main aflatoxigenic and ochratoxigenic species affecting foods. Food Control.

[239] Siddiqui T., Zia M.K., Muaz M., Ahsan H., Khan F.H. (2023). Synthesis and Characterization of Silver Nanoparticles (AgNPs) using Chemico-physical Methods. Ind. J. Chem. Anal..

[240] Nocerino V., Miranda B., Dardano P., Sanità G., Esposito E., De Stefano L. (2024). Protocol for synthesis of spherical silver nanoparticles with stable optical properties and characterization by transmission electron microscopy. STAR Protoc..

[241] Mateo E.M., Jiménez M. (2022). Silver Nanoparticle-Based Therapy: Can It Be Useful to Combat Multi-Drug Resistant Bacteria?. Antibiotics.

[242] Kirubakaran D., Abdul Wahid J.B., Karmegam N., Jeevika R., Sellapillai L., Rajkumar M., SenthilKumar K.J. (2025). A Comprehensive Review on the Green Synthesis of Nanoparticles: Advancements in Biomedical and Environmental Applications. Biomed. Mater. Devices.

[243] Ahmed S., Ahmad M., Swami B.L., Ikram S. (2016). A review on plants extract mediated synthesis of silver nanoparticles for antimicrobial applications: A green expertise. J. Adv. Res..

[244] Alharbi N.S., Alsubhi N.S., Felimban A.I. (2022). Green Synthesis of Silver Nanoparticles Using Medicinal Plants: Characterization and Application. J. Radiat. Res. Appl. Sci..

[245] Patil P.V., Nerlekar N.A., Kuldeep A.R., Patil P.P., Dandge P.B., Dongale T.D., Dandge P.B., Rashinkar G.S. (2024). Terminalia bellirica (Gaertn.) Roxb. Extract-Mediated Green Synthesis of Magnesium Oxide Nanoparticles for Multifunctional Applications. Plant Nano Biol..

[246] Kaur M., Gautam A., Guleria P., Singh K., Kumar V. (2022). Green synthesis of metal nanoparticles and their environmental applications. Curr. Opin. Environ. Sci. Health.

[247] Hussain I., Singh N.B., Singh A., Singh H., Singh S.C. (2016). Green synthesis of nanoparticles and its potential application. Biotechnol. Lett..

[248] Saxena R., Kotnala S., Bhatt S.C., Uniyal M., Rawat B.S., Negi P., Riyal M.K. (2025). A Review on Green Synthesis of Nanoparticles Toward Sustainable Environment. Sustain. Chem. Clim. Action.

[249] Ying S., Guan Z., Ofoegbu P.C., Clubb P., Rico C., He F., Hong J. (2022). Green Synthesis of Nanoparticles: Current Developments and Limitations. Environ. Technol. Innov..

[250] Alghuthaymi M.A., Rajkuberan C., Rajiv P., Kalia A., Bhardwaj K., Bhardwaj P., Abd-Elsalam K.A., Valis M., Kuca K. (2021). Nanohybrid Antifungals for Control of Plant Diseases: Current Status and Future Perspectives. J. Fungi.

[251] Vázquez-Muñoz R., Avalos-Borja M., Castro-Longoria E. (2014). Ultrastructural Analysis of *Candida albicans* When Exposed to Silver Nanoparticles. PLoS ONE.

[252] Selvaraj M., Pandurangan P., Ramasami N., Rajendran S.B., Sangilimuthu S.N., Perumal P. (2014). Highly potential antifungal activity of quantum-sized silver nanoparticles against *Candida albicans*. Appl. Biochem. Biotechnol..

[253] Athie-García M.S., Piñón-Castillo H.A., Muñoz-Castellanos L.N., Ulloa-Ogaz A.L., Martínez-Varela P.I., Quintero-Ramos A., Duran R., Murillo-Ramirez J.G., Orrantia-Borunda E. (2018). Cell wall damage and oxidative stress in *Candida albicans* ATCC 10231 and *Aspergillus niger* caused by palladium nanoparticles. Toxicol. In Vitro.

[254] Hwang I., Lee J., Hwang J.H., Kim K.J., Lee D.G. (2012). Silver Nanoparticles Induce Apoptotic Cell Death in *Candida albicans* through the Increase of Hydroxyl Radicals: Silver Nanoparticles Induce Apoptotic Cell Death. FEBS J..

[255] Abdal Dayem A., Hossain M.K., Lee S.B., Kim K., Saha S.K., Yang G.M., Choi H.Y., Cho S.G. (2017). The role of reactive oxygen species (ROS) in the Biological Activities of Metallic Nanoparticles. Int. J. Mol. Sci..

[256] Carmo P.H.F.d., Garcia M.T., Figueiredo-Godoi L.M.A., Lage A.C.P., Silva N.S.d., Junqueira J.C. (2023). Metal Nanoparticles to Combat *Candida albicans* Infections: An Update. Microorganisms.

[257] Lei H., Liu F., Jia M., Ni H., Han Y., Chen J., Wang H., Gu H., Chen Y., Lin Y. (2024). An Overview of the Direct Interaction of Synthesized Silver Nanostructures and Enzymes. Int. J. Biol. Macromol..

[258] Ribeiro A.I., Dias A.M., Zille A. (2022). Synergistic effects between metal nanoparticles and commercial antimicrobial agents: A review. ACS Appl. Nano Mater..

[259] Souza J.A.S., Alves M.M., Barbosa D.B., Lopes M.M., Pinto E., Figueiral M.H., Delbem A.C.B., Mira N.P. (2020). Study of the activity of *Punica granatum*-mediated silver nanoparticles against *Candida albicans* and *Candida glabrata*, alone or in combination with azoles or polyenes. Med. Mycol..

[260] Siddiqi K.S., Husen A., Rao R.A.K. (2018). A review on biosynthesis of silver nanoparticles and their biocidal properties. J. Nanobiotechnol..

[261] Kim T.-H., Kim M., Park H.-S., Shin U.S., Gong M.-S., Kim H.-W. (2012). Size-dependent cellular toxicity of silver nanoparticles. J. Biomed. Mater. Res. Part A.

[262] Kittler S., Greulich C., Diendorf J., Koller M., Epple M. (2010). Toxicity of silver nanoparticles increases during storage because of slow dissolution under release of silver ions. Chem. Mater..

[263] Meher A., Tandi A., Moharana S., Chakroborty S., Mohapatra S.S., Mondal A., Dey S., Chandra P. (2024). Silver nanoparticle for biomedical applications: A review. Hybrid Adv..

[264] Kong I.C., Ko K.-S., Koh D.-C. (2020). Evaluation of the effects of particle sizes of silver nanoparticles on various biological systems. Int. J. Mol. Sci..

[265] Gibała A., Zeliszewska P., Gosiewski T., Krawczyk A., Duraczyńska D., Szaleniec J., Szaleniec M., Oćwieja M. (2021). Antibacterial and Antifungal Properties of Silver Nanoparticles—Effect of a Surface-Stabilizing Agent. Biomolecules.

[266] Manosalva N., Tortella G., Cristina Diez M., Schalchli H., Seabra A.B., Durán N., Rubilar O. (2019). Green synthesis of silver nanoparticles: Effect of synthesis reaction parameters on antimicrobial activity. World J. Microbiol. Biotechnol..

[267] Oćwieja M., Barbasz A. (2020). Sodium Hexametaphosphate–Induced Enhancement of Silver Nanoparticle Toxicity towards Leukemia Cells. J. Nanopart. Res..

[268] Tomak A., Yilancioglu B., Winkler D., Karakus C.O. (2022). Protein corona formation on silver nanoparticles under different conditions. Colloids Surf. A Physicochem. Eng. Aspects.

[269] Fröhlich E. (2012). The role of surface charge in cellular uptake and cytotoxicity of medical nanoparticles. Int. J. Nanomed..

[270] Matras E., Gorczyca A., Przemieniecki S.W., Oćwieja M. (2022). Surface properties-dependent antifungal activity of silver nanoparticles. Sci. Rep..

[271] Rai M., Kon K., Ingle A., Duran N., Galdiero S., Galdiero M. (2014). Broad-Spectrum Bioactivities of Silver Nanoparticles: The Emerging Trends and Future Prospects. Appl. Microbiol. Biotechnol..

[272] Rodrigues A.S., Batista J.G.S., Rodrigues M.Á.V., Thipe V.C., Minarini L.A.R., Lopes P.S., Lugão A.B. (2024). Advances in silver nanoparticles: A comprehensive review on their potential as antimicrobial agents and their mechanisms of action elucidated by proteomics. Front. Microbiol..

[273] Madkhali O.A. (2023). A Comprehensive Review on Potential Applications of Metallic Nanoparticles as Antifungal Therapies to Combat Human Fungal Diseases. Saudi Pharm. J..

[274] Dell’Annunziata F., Mosidze E., Folliero V., Lamparelli E.P., Lopardo V., Pagliano P., Della Porta G., Galdiero M., Bakuridze A.D., Franci G. (2024). Eco-friendly synthesis of silver nanoparticles from peel and juice C. limon and their antiviral efficacy against HSV-1 and SARS-CoV-2. Virus Res..

[275] Tarazona A., Gómez J.V., Mateo E.M., Jiménez M., Mateo F. (2019). Antifungal effect of engineered silver nanoparticles on phytopathogenic and toxigenic *Fusarium* spp. and their impact on mycotoxin accumulation. Int. J. Food Microbiol..

[276] Sedaghati E., Molaei S., Molaei M., Doraki N. (2018). An evaluation of antifungal and antitoxigenicity effects of Ag/Zn and Ag nanoparticles on *Aspergillus parasiticus* growth and aflatoxin production. PHJ.

[277] Al-Othman M.R., Abd El-Aziz A.R.M., Mahmoud M.A., Eifan S.A., El-Shikh M.S., Majrashi M. (2014). Application of silver nanoparticles as antifungal and antiaflatoxin B_1_ produced by *Aspergillus flavus*. Dig. J. Nanomater. Biostruct..

[278] El-Naggar M.A., Alrajhi A.M., Fouda M.M., Abdelkareem E.M., Thabit T.M., Bouqellah N.A. (2018). Effect of Silver Nanoparticles on Toxigenic *Fusarium* spp. and Deoxynivalenol Secretion in Some Grains. J. AOAC Int..

[279] Ibrahim E., Zhang M., Zhang Y., Hossain A., Qiu W., Chen Y., Wang Y., Wu W., Sun G., Li B. (2020). Green-Synthesization of Silver Nanoparticles Using Endophytic Bacteria Isolated from Garlic and Its Antifungal Activity Against Wheat *Fusarium* Head Blight Pathogen *Fusarium graminearum*. Nanomaterials.

[280] El-Desouky T.A., Ammar H.A.M. (2016). Honey Mediated Silver Nanoparticles and Their Inhibitory Effect on Aflatoxins and Ochratoxin A. J. Appl. Pharm. Sci..

[281] Ammar H.A.M., El-Desouky T.A. (2016). Green synthesis of nanosilver particles by *Aspergillus terreus* HA1N and *Penicillium expansum* HA2N and its antifungal activity against mycotoxigenic fungi. J. Appl. Microbiol..

[282] Mousavi S.A.A., Pourtalebi S. (2015). Inhibitory Effects of Silver Nanoparticles on Growth and Aflatoxin B1 Production by *Aspergillus parasiticus*. Iran. J. Med. Sci..

[283] Kotzybik K., Gräf V., Kugler L., Stoll D.A., Greiner R., Geisen R., Schmidt-Heydt M. (2016). Influence of different nanomaterials on growth and mycotoxin production of *Penicillium verrucosum*. PLoS ONE.

[284] Khalil N.M., Abd El-Ghany M.N., Rodríguez-Couto S. (2019). Antifungal and anti-mycotoxin efficacy of biogenic silver nanoparticles produced by *Fusarium chlamydosporum* and *Penicillium chrysogenum* at non-cytotoxic doses. Chemosphere.

[285] Pérez-de León A., Plasencia J., Vázquez-Durán A., Méndez-Albores A. (2020). Comparison of the In Vitro Antifungal and Anti-Fumonigenic Activities of Copper and Silver Nanoparticles Against *Fusarium verticillioides*. J. Clust. Sci..

[286] Jo Y.-K., Cromwell W., Jeong H.-K., Thorkelson J., Roh J.-H., Shin D.-B. (2015). Use of silver nanoparticles for managing *Gibberella fujikuroi* on rice seedlings. Crop Prot..

[287] Baigorria C.G., Cerioni L., Debes M.A., Ledesma A.E., Alastuey P., Tirado M., Volentini S.I., Rapisarda V.A. (2024). Antifungal action of metallic nanoparticles against fungicide-resistant pathogens causing main postharvest lemon diseases. J. Fungi.

[288] Bocate K.P., Reis G.F., de Souza P.C., Oliveira Junior A.G., Durán N., Nakazato G., Furlaneto M.C., de Almeida R.S., Panagio L.A. (2019). Antifungal activity of silver nanoparticles and simvastatin against toxigenic species of *Aspergillus*. Int. J. Food Microbiol..

[289] Al-Otibi F., Perveen K., Al-Saif N.A., Alharbi R.I., Bokhari N.A., Albasher G., Al-Otaibi R.M., Al-Mosa M.A. (2021). Biosynthesis of silver nanoparticles using *Malva parviflora* and their antifungal activity. Saudi J. Biol. Sci..

[290] Madbouly A.K., Abdel-Aziz M.S., Abdel-Wahhab M.A. (2017). Biosynthesis of nanosilver using *Chaetomium globosum* and its application to control *Fusarium* wilt of tomato in the greenhouse. IET Nanobiotechnol..

[291] Qian Y., Yu H., He D., Yang H., Wang W., Wan X., Wang L. (2013). Biosynthesis of Silver Nanoparticles by the Endophytic Fungus *Epicoccum nigrum* and Their Activity against Pathogenic Fungi. Bioprocess Biosyst. Eng..

[292] Valsalam S., Agastian P., Arasu M.V., Al-Dhabi N.A., Ghilan A.-K.M., Kaviyarasu K., Ravindran B., Chang S.W., Arokiyaraj S. (2018). Rapid Biosynthesis and Characterization of Silver Nanoparticles from the Leaf Extract of *Tropaeolum majus* L. and Its Enhanced In-Vitro Antibacterial, Antifungal, Antioxidant and Anticancer Properties. J. Photochem. Photobiol. B Biol..

[293] Amrinder K., Jaspal K., Anu K., Narinder S. (2016). Effect of media composition on extent of antimycotic activity of silver nanoparticles against plant pathogenic fungus *Fusarium moniliforme*. Plant Dis. Res..

[294] Macías Sánchez K.L., González Martínez H.D.R., Carrera Cerritos R., Martínez Espinosa J.C. (2023). In vitro evaluation of the antifungal effect of AgNPs on *Fusarium oxysporum* f. sp. *lycopersici*. Nanomaterials.

[295] Jafari A., Pourakbar L., Farhadi K., Mohamadgolizad L., Goosta Y. (2015). Biological synthesis of silver nanoparticles and evaluation of antibacterial and antifungal properties of silver and copper nanoparticles. Turk. J. Biol..

[296] Xue B., He D., Gao S., Wang D., Yokoyama K., Wang L. (2016). Biosynthesis of Silver Nanoparticles by the Fungus *Arthroderma fulvum* and Its Antifungal Activity Against Genera of *Candida*, *Aspergillus* and *Fusarium*. Int. J. Nanomed..

[297] Bahrami-Teimoori B., Nikparast Y., Hojatianfar M., Akhlaghi M., Ghorbani R., Pourianfar H.R. (2017). Characterisation and antifungal activity of silver nanoparticles biologically synthesised by *Amaranthus retroflexus* leaf extract. J. Exp. Nanosci..

[298] Nguyen D.H., Lee J.S., Park K.D., Ching Y.C., Nguyen X.T., Phan V.G., Thi T.T.H. (2020). Green Silver Nanoparticles Formed by *Phyllanthus urinaria*, *Pouzolzia zeylanica*, and *Scoparia dulcis* Leaf Extracts and the Antifungal Activity. Nanomaterials.

[299] Abdel-Hadi A.M., Awad M.F., Abo-Dahab N.F., ElKady M.F. (2014). Extracellular synthesis of silver nanoparticles by *Aspergillus terreus*: Biosynthesis, characterization, and biological activity. Biosci. Biotechnol. Res. Asia.

[300] Elamawi R.M., Al-Harbi R.E., Hendi A.A. (2018). Biosynthesis and Characterization of Silver Nanoparticles Using *Trichoderma longibrachiatum* and Their Effect on Phytopathogenic Fungi. Egypt J. Biol. Pest Control.

[301] Nguyen D.H., Vo T.N.N., Nguyen N.T., Ching Y.C., Thi T.T.H. (2020). Comparison of Biogenic Silver Nanoparticles Formed by *Momordica charantia* and *Psidium guajava* Leaf Extract and Antifungal Evaluation. PLoS ONE.

[302] Fernández J.G., Fernández-Baldo M.A., Berni E., Camí G., Durán N., Raba J., Sanz M.I. (2016). Production of silver nanoparticles using yeasts and evaluation of their antifungal activity against phytopathogenic fungi. Process Biochem..

[303] Win T.T., Khan S., Fu P. (2020). Fungus (*Alternaria* sp.) Mediated Silver Nanoparticles Synthesis, Characterization, and Screening of Antifungal Activity against some Phytopathogens. J. Nanotechnol..

[304] Yassin M.A., Elgorban A.M., El-Samawaty A.E.-R.M., Almunqedhi B.M. (2021). Biosynthesis of Silver Nanoparticles Using *Penicillium verrucosum* and Analysis of Their Antifungal Activity. Saudi J. Biol. Sci..

[305] Malik M., Wani W.A., Bhat M.A., Siddiqui M.A., Alamri S., Alrumman S.A. (2024). Fungal-Mediated Synthesis of Silver Nanoparticles: A Novel Strategy for Plant Disease Management. Toxins.

[306] Khan A.U., Khan M., Khan M.M. (2019). Antifungal and antibacterial assay by silver nanoparticles synthesized from aqueous leaf extract of *Trigonella foenum-graecum*. BioNanoScience.

[307] Dawoud T.M., Yassin M.A., El-Samawaty A.R.M., Elgorban A.M. (2021). Silver Nanoparticles Synthesized by *Nigrospora oryzae* Showed Antifungal Activity. Saudi J. Biol. Sci..

[308] Gautam N., Salaria N., Thakur K., Bhardwaj A., Awasthi A., Kumar V. (2020). Green Silver Nanoparticles for Phytopathogen Control. Proc. Natl. Acad. Sci. USA India Sect. B Biol. Sci..

[309] Tabassum R.Z., Mehmood A., Khalid A.U.R., Ahmad K.S., Khan M.A.R., Amjad M.S., Raffi M., Khan G.-e.-L., Mustafa A. (2024). Green synthesis of silver nanoparticles for antifungal activity against tomato fusarium wilt caused by *Fusarium oxysporum*. Biocatal. Agric. Biotechnol..

[310] Mendoza N.V., Yánez P., Magdama F., Pacheco R., Vielma J., Vanegas M.E., Bogdanchikova N., Pestryakov A., Chong P. (2025). Inhibition of *Fusarium oxysporum* Growth in Banana by Silver Nanoparticles: In Vitro and In Vivo Assays. PLoS ONE.

[311] Kim S.W., Jung J.H., Lamsal K., Kim Y.S., Min J.S., Lee Y.S. (2012). Antifungal effects of silver nanoparticles (AgNPs) against various plant pathogenic fungi. Mycobiology.

[312] Aleksandrowicz-Trzcińska M., Szaniawski A., Olchowik J., Drozdowski S. (2018). Effects of copper and silver nanoparticles on growth of selected species of pathogenic and wood-decay fungi in vitro. For. Chron..

[313] Asghar M.A., Zahir E., Shahid S.M., Khan M.N., Iqbal J., Walker G. (2018). Iron, copper and silver nanoparticles: Green synthesis using green and black tea leaves extracts and evaluation of antibacterial, antifungal and aflatoxin B1 adsorption activity. LWT.

[314] Asghar M.A., Zahir E., Asghar M.A., Iqbal J., Rehman A.A. (2020). Facile, one-pot biosynthesis and characterization of iron, copper and silver nanoparticles using *Syzygium cumini* leaf extract: As an effective antimicrobial and aflatoxin B_1_ adsorption agents. PLoS ONE.

[315] Dananjaya S.H.S., Erandani W.K.C.U., Kim C.-H., Nikapitiya C., Lee J., De Zoysa M. (2017). Comparative study on antifungal activities of chitosan nanoparticles and chitosan silver nanocomposites against *Fusarium oxysporum* species complex. Int. J. Biol. Macromol..

[316] Yassin M.A., El-Samawaty A.E.M.A., Dawoud T.M., Abd-Elkader O.H., Al Maary K.S., Hatamleh A.A., Elgorban A.M. (2017). Characterization and Anti-*Aspergillus flavus* Impact of Nanoparticles Synthesized by *Penicillium citrinum*. Saudi J. Biol. Sci..

[317] Tang H., Xu M., Luo J., Zhao L., Ye G., Shi F., Lv C., Chen H., Wang Y., Li Y. (2019). Liver toxicity assessments in rats following sub-chronic oral exposure to copper nanoparticles. Environ. Sci. Eur..

[318] He X., Deng H., Hwang H.M. (2019). The Current Application of Nanotechnology in Food and Agriculture. J. Food Drug Anal..

[319] Khamis Y., Hashim A.F., Margarita R., Alghuthaymi M.A., Abd-Elsalam K.A. (2017). Fungicidal efficacy of chemically-produced copper nanoparticles against *Penicillium digitatum* and *Fusarium solani* on citrus fruit. Philipp. Agric. Sci..

[320] Nath A., Molnár M.A., Albert K., Das A., Bánvölgyi S., Márki E., Vatai G. (2019). Agrochemicals from Nanomaterials—Synthesis, Mechanisms of Biochemical Activities and Applications. Comprehensive Analytical Chemistry.

[321] Sidhu A., Barmota H., Bala A. (2017). Antifungal evaluation studies of copper sulfide nano-aquaformulations and its impact on seed quality of rice (*Oryzae sativa*). Appl. Nanosci..

[322] Wang P., Yuan Y., Xu K., Zhong H., Yang Y., Jin S., Yang K., Qi X. (2021). Biological applications of copper-containing materials. Bioact. Mater..

[323] Konappa N., Krishnamurthy S., Arakere U.C., Chowdappa S., Akbarbasha R., Ramachandrappa N.S., Jogaiah S., Singh H.B., Fraceto L.F., de Lima R. (2021). Nanofertilizers and Nanopesticides: Recent Trends, Future Prospects in Agriculture. Woodhead Publishing Series in Food Science, Technology and Nutrition.

[324] Tegenaw A., Tolaymat T., Al-Abed S., El Badawy A., Luxton T., Sorial G., Genaidy A. (2015). Characterization and Potential Environmental Implications of Select Cu-Based Fungicides and Bactericides Employed in U.S. Markets. Environ. Sci. Technol..

[325] López-Lima D., Mtz-Enriquez A.I., Carrión G., Basurto-Cereceda S., Pariona N. (2020). The bifunctional role of copper nanoparticles in tomato: Effective treatment for *Fusarium* wilt and plant growth promoter. Sci. Hortic..

[326] Kanhed P., Birla S., Gaikwad S., Gade A., Seabra A.B., Rubilar O., Durán N., Rai M. (2014). In vitro antifungal efficacy of copper nanoparticles against selected crop pathogenic fungi. Mater. Lett..

[327] Ingle A.P., Rai M. (2017). Copper Nanoflowers as Effective Antifungal Agents for Plant Pathogenic Fungi. IET Nanobiotechnol..

[328] Hermida-Montero L., Pariona N., Mtz-Enriquez A.I., Carrión G., Paraguay-Delgado F., Rosas-Saito G. (2019). Aqueous-Phase Synthesis of Nanoparticles of Copper/Copper Oxides and Their Antifungal Effect Against *Fusarium oxysporum*. J. Hazard. Mater..

[329] Van Viet P., Nguyen H.T., Cao T.M., Van Hieu L., Pham V. (2016). *Fusarium* Antifungal Activities of Copper Nanoparticles Synthesized by a Chemical Reduction Method. J. Nanomater..

[330] Seku K., Reddy G.B., Pejjai B., Kotu G.M., Narasimha G. (2018). Hydrothermal Synthesis of Copper Nanoparticles, Characterization and Their Biological Applications. Int. J. Nano Dimens..

[331] Ghasemian E., Naghoni A., Tabaraie B., Tabaraie T. (2012). In Vitro Susceptibility of Filamentous Fungi to Copper Nanoparticles Assessed by Rapid XTT Colorimetry and Agar Dilution Method. J. Mycol. Méd..

[332] Pham N.-D., Duong M.-M., Le M.-V., Hoang H.A., Pham L.-K. (2019). Preparation and Characterization of Antifungal Colloidal Copper Nanoparticles and Their Antifungal Activity against *Fusarium oxysporum* and *Phytophthora capsici*. C. R. Chim..

[333] Bramhanwade K., Shende S., Bonde S., Gade A., Rai M. (2016). Fungicidal activity of Cu nanoparticles against *Fusarium* causing crop diseases. Environ. Chem. Lett..

[334] Pariona N., Mtz-Enriquez A.I., Sánchez-Rangel D., Carrión G., Paraguay-Delgado F., Rosas-Saito G. (2019). Green-Synthesized Copper Nanoparticles as a Potential Antifungal against Plant Pathogens. RSC Adv..

[335] Maqsood S., Qadir S., Hussain A., Asghar A., Saleem R., Zaheer S., Nayyar N. (2020). Antifungal properties of copper nanoparticles against *Aspergillus niger*. Sch. Int. J. Biochem..

[336] Shende S., Ingle A.P., Gade A., Rai M. (2015). Green Synthesis of Copper Nanoparticles by *Citrus medica* Linn. (Idilimbu) Juice and Its Antimicrobial Activity. World J. Microbiol. Biotechnol..

[337] Hasanin M., Al Abboud M.A., Alawlaqi M.M., Abdelghany T.M., Hashem A.H. (2022). Ecofriendly Synthesis of Biosynthesized Copper Nanoparticles with Starch-Based Nanocomposite: Antimicrobial, Antioxidant, and Anticancer Activities. Biol. Trace Elem. Res..

[338] Mali S.C., Dhaka A., Githala C.K., Trivedi R. (2020). Green synthesis of copper nanoparticles using *Celastrus paniculatus* Willd. leaf extract and their photocatalytic and antifungal properties. Biotechnol. Rep..

[339] Malandrakis A.A., Kavroulakis N., Chrysikopoulos C.V. (2019). Use of Copper, Silver and Zinc Nanoparticles against Foliar and Soil-Borne Plant Pathogens. Sci. Total Environ..

[340] Priya M., Venkatesan R., Deepa S., Babu S., Kavitha S., Sekar M., Chen W.C., Chen J.H. (2023). Green synthesis, characterization, antibacterial, and antifungal activity of copper oxide nanoparticles derived from Morinda citrifolia leaf extract. Sci. Rep..

[341] Mohamed A.A., Abu-Elghait M., Ahmed N.E., Salem S.S. (2021). Eco-Friendly Mycogenic Synthesis of ZnO and CuO Nanoparticles for In Vitro Antibacterial, Antibiofilm, and Antifungal Applications. Biol. Trace Elem. Res..

[342] Devipriya D., Roopan S.M. (2017). *Cissus quadrangularis* Mediated Ecofriendly Synthesis of Copper Oxide Nanoparticles and Its Antifungal Studies Against *Aspergillus niger* and *Aspergillus flavus*. Mater. Sci. Eng. C.

[343] Shammout M.W., Awwad A.M. (2021). A novel route for the synthesis of copper oxide nanoparticles using Bougainvillea plant flowers extract and antifungal activity evaluation. Chem. Int..

[344] Vanathi P., Rajiv P., Sivaraj R. (2016). Synthesis and Characterization of Eichhornia-Mediated Copper Oxide Nanoparticles and Assessing Their Antifungal Activity Against Plant Pathogens. Bull. Mater. Sci..

[345] Sardar M., Ahmed W., Al Ayoubi S., Nisa S., Bibi Y., Sabir M., Khan M.M., Ahmed W., Qayyum A. (2022). Fungicidal synergistic effect of biogenically synthesized zinc oxide and copper oxide nanoparticles against *Alternaria citri* causing citrus black rot disease. Saudi J. Biol. Sci..

[346] El-Batal A.I., El-Sayyad G.S., Mosallam F.M., Fathy R.M. (2020). *Penicillium chrysogenum*-Mediated Mycogenic Synthesis of Copper Oxide Nanoparticles Using Gamma Rays for In Vitro Antimicrobial Activity Against Some Plant Pathogens. J. Clust. Sci..

[347] Vera-Reyes I., Esparza-Arredondo I.J.E., Lira-Saldivar R.H., Granados-Echegoyen C.A., Alvarez-Roman R., Vásquez-López A., Díaz-Barriga Castro E. (2019). In Vitro Antimicrobial Effect of Metallic Nanoparticles on Phytopathogenic Strains of Crop Plants. J. Phytopathol..

[348] Cuajungco M.P., Ramirez M.S., Tolmasky M.E. (2021). Zinc: Multidimensional Effects on Living Organisms. Biomedicines.

[349] Jha S., Rani R., Singh S. (2023). Biogenic zinc oxide nanoparticles and their biomedical applications: A review. J. Inorg. Organomet. Polym. Mater..

[350] Lin J., Bjørk P.K., Kolte M.V., Poulsen E., Dedic E., Drace T., Andersen S.U., Nadzieja M., Liu H., Castillo-Michel H. (2024). Zinc mediates control of nitrogen fixation via transcription factor filamentation. Nature.

[351] Raha S., Ahmaruzzaman M. (2022). ZnO Nanostructured Materials and Their Potential Applications: Progress, Challenges and Perspectives. Nanoscale Adv..

[352] Gomaa E.Z. (2022). Microbial Mediated Synthesis of Zinc Oxide Nanoparticles, Characterization and Multifaceted Applications. J. Inorg. Organomet. Polym..

[353] Siddiqi K.S., ur Rahman A., Tajuddin A., Husen A., Al-Warthan A., Al-Muhtaseb A.H. (2018). Properties of Zinc Oxide Nanoparticles and Their Activity Against Microbes. Nanoscale Res. Lett..

[354] Mirzaei H., Darroudi M. (2017). Synthesis and Characterization of Zinc Oxide Nanoparticles: A Review. Ceram. Int..

[355] Rehman A., Khan S., Sun F., Peng Z., Feng K., Wang N., Jia Y., Pan Z., He S., Wang L. (2024). Exploring the nano-wonders: Unveiling the role of nanoparticles in enhancing salinity and drought tolerance in plants. Front. Plant Sci..

[356] Smaoui S., Chérif I., Ben Hlima H., Khan M.U., Rebezov M., Thiruvengadam M., Sarkar T., Shariati M.A., Lorenzo J.M. (2023). Zinc Oxide Nanoparticles in Meat Packaging: A Systematic Review of Recent Literature. Food Packag. Shelf Life.

[357] Gökmen G.G., Mirsafi F.S., Leissner T., Akan T., Mishra Y.K., Kışla D. (2024). Zinc Oxide Nanomaterials: Safeguarding Food Quality and Sustainability. Compr. Rev. Food Sci. Food Saf..

[358] Ferri M., Papchenko K., Degli Esposti M., Tondi G., De Angelis M.G., Morselli D., Fabbri P. (2022). Emerging Trends for ZnO Nanoparticles and Their Applications in Food Packaging. ACS Food Sci. Technol..

[359] Dey S., Mohanty D.L., Mohanty D., Divya N., Bakshi V., Mohanty A., Rath D., Das S., Mondal A., Roy S. (2024). A Critical Review on Zinc Oxide Nanoparticles: Synthesis, Properties and Biomedical Applications. Int. Pharm..

[360] Hussain I., Malik F., Shah S., Al-Kahtani M.A., Almaghasla M.I., Al-Dosary M., Al-Shehri M., Al-Rashdi A.S., Shah T., Al-Kahtani J. (2022). Efficacy of Biogenic Zinc Oxide Nanoparticles in Treating Wastewater for Sustainable Wheat Cultivation. Agronomy.

[361] Hussain R.T., Hossain M.S., Shariffuddin J.H. (2024). Green Synthesis and Photocatalytic Insights: A Review of Zinc Oxide Nanoparticles in Wastewater Treatment. Mater. Today Sustain..

[362] Dimapilis E.A.S., Hsu C.S., Mendoza R.M.O., Lu M.C. (2018). Zinc Oxide Nanoparticles for Water Disinfection. Sustain. Environ. Res..

[363] Nawaz A., Farhan A., Maqbool F., Ahmad H., Qayyum W., Ghazy E., Rahdar A., Díez-Pascual A.M., Fathi-Karkan S. (2024). Zinc Oxide Nanoparticles: Pathways to Micropollutant Adsorption, Dye Removal, and Antibacterial Actions—A Study of Mechanisms, Challenges, and Future Prospects. J. Mol. Struct..

[364] Sun Q., Li J., Le T. (2018). Zinc Oxide Nanoparticle as a Novel Class of Antifungal Agents: Current Advances and Future Perspectives. J. Agric. Food Chem..

[365] Pariona N., Paraguay-Delgado F., Basurto-Cereceda S., Morales-Mendoza J.E., Hermida-Montero L.A., Mtz-Enriquez A.I. (2020). Shape-Dependent Antifungal Activity of ZnO Particles against Phytopathogenic Fungi. Appl. Nanosci..

[366] Savi G.D., Bortoluzzi A.J., Scussel V.M. (2013). Antifungal properties of Zinc-compounds against toxigenic fungi and mycotoxin. Int. J. Food Sci. Technol..

[367] He L., Liu Y., Mustapha A., Lin M. (2011). Antifungal activity of zinc oxide nanoparticles against *Botrytis cinerea* and *Penicillium expansum*. Microbiol. Res..

[368] Akpomie K.G., Ghosh S., Gryzenhout M., Conradie J. (2021). One-pot synthesis of zinc oxide nanoparticles via chemical precipitation for bromophenol blue adsorption and the antifungal activity against filamentous fungi. Sci. Rep..

[369] Wani A.H., Shah M.A. (2012). A Unique and Profound Effect of MgO and ZnO Nanoparticles on Some Plant Pathogenic Fungi. J. Appl. Pharm. Sci..

[370] Hassan A.A., Howayda M.E., Mahmoud H.H. (2013). Effect of Zinc Oxide Nanoparticles on the Growth of Mycotoxigenic Mould. J. Stud. Chem. Process Technol..

[371] Zaki S.A., Ouf S.A., Albarakaty F.M., Habeb M.M., Aly A.A., Abd-Elsalam K.A. (2021). *Trichoderma harzianum*-Mediated ZnO Nanoparticles: A Green Tool for Controlling Soil-Borne Pathogens in Cotton. J. Fungi.

[372] Zhu W., Hu C., Ren Y., Lu Y., Song Y., Ji Y., He J. (2021). Green Synthesis of Zinc Oxide Nanoparticles Using *Cinnamomum camphora* (L.) Presl Leaf Extracts and Its Antifungal Activity. J. Environ. Chem. Eng..

[373] Jayaseelan C., Rahuman A.A., Kirthi A.V., Marimuthu S., Santhoshkumar T., Bagavan A., Gaurav K., Karthik L., Rao K.V.B. (2012). Novel microbial route to synthesize ZnO nanoparticles using *Aeromonas hydrophila* and their activity against pathogenic bacteria and fungi. Spectrochim. Acta Part A Mol. Biomol. Spectrosc..

[374] Rajiv P., Rajeshwari S., Venckatesh R. (2013). Bio-Fabrication of Zinc Oxide Nanoparticles Using Leaf Extract of *Parthenium hysterophorus* L. and Its Size-Dependent Antifungal Activity against Plant Fungal Pathogens. Spectrochim. Acta Part A Mol. Biomol. Spectrosc..

[375] Jamdagni P., Khatri P., Rana J.S. (2018). Green synthesis of zinc oxide nanoparticles using flower extract of Nyctanthes arbor-tristis and their antifungal activity. J. King Saud Univ. Sci..

[376] Kamal A., Saba M., Kamal A., Batool M., Asif M., Al-Mohaimeed A.M., Al Farraj D.A., Habib D., Ahmad S. (2023). Bioinspired green synthesis of bimetallic iron and zinc oxide nanoparticles using mushroom extract and use against *Aspergillus niger*; the most devastating fungi of the green world. Catalysts.

[377] Alhazmi N.M., Sharaf E.M. (2023). Fungicidal activity of zinc oxide nanoparticles against azole-resistant *Aspergillus flavus* isolated from yellow and white maize. Molecules.

[378] Sharma R., Sharma R., Singh R.R., Kumari A. (2023). Evaluation of biogenic zinc oxide nanoparticles from Tinospora cordifolia stem extract for photocatalytic, anti-microbial, and antifungal activities. Mater. Chem. Phys..

[379] Jain D., Shivani, Bhojiya A.A., Singh H., Daima H.K., Singh M., Mohanty S.R., Stephen B.J., Singh A. (2020). Microbial fabrication of zinc oxide nanoparticles and evaluation of their antimicrobial and photocatalytic properties. Front. Chem..

[380] Karkhane M., Lashgarian H.E., Mirzaei S.Z., Ghaffarizadeh A., Sepahvand A., Marzban A. (2020). Antifungal, antioxidant and photocatalytic activities of zinc nanoparticles synthesized by *Sargassum vulgare* extract. Biocatal. Agric. Biotechnol..

[381] Lakshmeesha T.R., Murali M., Ansari M.A., Udayashankar A.C., Alzohairy M.A., Almatroudi A., Niranjana S.R. (2020). Biofabrication of Zinc Oxide Nanoparticles from *Melia azedarach* and Its Potential in Controlling Soybean Seed-Borne Phytopathogenic Fungi. Saudi J. Biol. Sci..

[382] Madhumitha G., Fowsiya J., Gupta N., Kumar A., Singh M. (2019). Green synthesis, characterization, and antifungal and photocatalytic activity of *Pithecellobium dulce* peel–mediated ZnO nanoparticles. J. Phys. Chem. Solids.

[383] Kolahalam L.A., Prasad K.R.S., Krishna P.M., Supraja N. (2021). *Saussurea lappa* plant rhizome extract-based zinc oxide nanoparticles: Synthesis, characterization and its antibacterial, antifungal activities and cytotoxic studies against Chinese hamster ovary (CHO) cell lines. Heliyon.

[384] Sardella D., Gatt R., Valdramidis V.P. (2017). Physiological effects and mode of action of ZnO nanoparticles against postharvest fungal contaminants. Food Res. Int..

[385] Malandrakis A.A., Kavroulakis N., Chrysikopoulos C.V. (2022). Zinc nanoparticles: Mode of action and efficacy against boscalid-resistant *Alternaria alternata* isolates. Sci. Total Environ..

[386] Yehia R.S., Ahmed O.F. (2013). In Vitro Study of the Antifungal Efficacy of Zinc Oxide Nanoparticles Against *Fusarium oxysporum* and *Penicillium expansum*. Afr. J. Microbiol. Res..

[387] Zudyte B., Luksiene Z. (2021). Visible Light-Activated ZnO Nanoparticles for Microbial Control of Wheat Crop. J. Photochem. Photobiol. B Biol..

[388] Koka J.A., Wani A.H., Bhat M.Y. (2019). Evaluation of antifungal activity of magnesium oxide (MgO) and iron oxide (FeO) nanoparticles on rot causing fungi. J. Drug Deliv. Ther..

[389] Silvestri L., Pettinato M., Furiosi V., Bavuso Volpe L., Nai A., Pagani A. (2023). Managing the dual nature of iron to preserve health. Int. J. Mol. Sci..

[390] Rolić T., Yazdani M., Mandić S., Distante S. (2024). Iron metabolism, calcium, magnesium and trace elements: A review. A review. Biol. Trace Elem. Res..

[391] Al Alawi A.M., Majoni S.W., Falhammar H. (2018). Magnesium and Human Health: Perspectives and Research Directions. Int. J. Endocrinol..

[392] Ahari H., Lahijani L.K. (2021). Migration of Silver and Copper Nanoparticles from Food Coating. Coatings.

[393] Störmer A., Bott J., Kemmer D., Franz R. (2017). Critical review of the migration potential of nanoparticles in food contact plastics. Trends Food Sci. Technol..

[394] Ahari H., Jafari A., Ozdal T., Moradi S., Bahari H.R., Wu Q., Eş I., Khaneghah A.M. (2025). Recent innovations in metal-based nanoparticles for food packaging: A focus on safety and environmental impact. Appl. Food Res..

[395] Rothen-Rutishauser B., Bogdanovich M., Harter R., Milosevic A., Petri-Fink A. (2021). Use of nanoparticles in food industry: Current legislation, health risk discussions and public perception with a focus on Switzerland. Toxicol. Environ. Chem..

[396] Rao M.V.M., Mohammad N., Banerjee S., Khanna P.K. (2024). Synthesis and Food Packaging Application of Silver Nanoparticles: A Review. Hybrid Adv..

[397] Zhang W., Roy S., Rhim J.W. (2023). Copper-Based Nanoparticles for Biopolymer-Based Functional Films in Food Packaging Applications. Compr. Rev. Food Sci. Food Saf..

[398] Gopinath K., Sathishkumar G., Xu L. (2024). An Overview of the Copper Oxide Nanofillers Integrated in Food Packaging Systems. Coatings.

[399] Herrera-Rivera M.D.R., Torres-Arellanes S.P., Cortés-Martínez C.I., Navarro-Ibarra D.C., Hernández-Sánchez L., Solis-Pomar F., Pérez-Tijerina E., Román-Doval R. (2024). Nanotechnology in Food Packaging Materials: Role and Application of Nanoparticles. RSC Adv..

[400] Joshi N.C., Negi P.B., Gururani P.A. (2024). Review on Metal/Metal Oxide Nanoparticles in Food Processing and Packaging. Food Sci. Biotechnol..

[401] Adeyemi J.O., Fawole O.A. (2023). Metal-Based Nanoparticles in Food Packaging and Coating Technologies: A Review. Biomolecules.

